# Impact of Nonsense-Mediated mRNA Decay on the Global Expression Profile of Budding Yeast

**DOI:** 10.1371/journal.pgen.0020203

**Published:** 2006-11-24

**Authors:** Qiaoning Guan, Wei Zheng, Shijie Tang, Xiaosong Liu, Robert A Zinkel, Kam-Wah Tsui, Brian S Yandell, Michael R Culbertson

**Affiliations:** 1 Laboratories of Genetics and Molecular Biology, University of Wisconsin, Madison, Wisconsin, United States of America; 2 Department of Statistics, University of Wisconsin, Madison, Wisconsin, United States of America; 3 Department of Physics, University of Wisconsin, Madison, Wisconsin, United States of America; 4 Department of Horticulture, University of Wisconsin, Madison, Wisconsin, United States of America; Princeton University, United States of America

## Abstract

Nonsense-mediated mRNA decay (NMD) is a eukaryotic mechanism of RNA surveillance that selectively eliminates aberrant transcripts coding for potentially deleterious proteins. NMD also functions in the normal repertoire of gene expression. In *Saccharomyces cerevisiae,* hundreds of endogenous RNA Polymerase II transcripts achieve steady-state levels that depend on NMD. For some, the decay rate is directly influenced by NMD (direct targets). For others, abundance is NMD-sensitive but without any effect on the decay rate (indirect targets). To distinguish between direct and indirect targets, total RNA from wild-type (Nmd^+^) and mutant (Nmd^−^) strains was probed with high-density arrays across a 1-h time window following transcription inhibition. Statistical models were developed to describe the kinetics of RNA decay. 45% ± 5% of RNAs targeted by NMD were predicted to be direct targets with altered decay rates in Nmd^−^ strains. Parallel experiments using conventional methods were conducted to empirically test predictions from the global experiment. The results show that the global assay reliably distinguished direct versus indirect targets. Different types of targets were investigated, including transcripts containing adjacent, disabled open reading frames, upstream open reading frames, and those prone to out-of-frame initiation of translation. Known targeting mechanisms fail to account for all of the direct targets of NMD, suggesting that additional targeting mechanisms remain to be elucidated. 30% of the protein-coding targets of NMD fell into two broadly defined functional themes: those affecting chromosome structure and behavior and those affecting cell surface dynamics. Overall, the results provide a preview for how expression profiles in multi-cellular eukaryotes might be impacted by NMD. Furthermore, the methods for analyzing decay rates on a global scale offer a blueprint for new ways to study mRNA decay pathways in any organism where cultured cell lines are available.

## Synopsis

Genes determine the structure of proteins through transcription and translation in which an RNA copy of the gene is made (mRNA) and then translated to make the protein. Cellular protein levels reflect the relative rates of mRNA synthesis and degradation, which are subject to multiple layers of controls. Mechanisms also exist to ensure the quality of each mRNA. One quality control mechanism called nonsense-mediated mRNA decay (NMD) triggers the rapid degradation of mRNAs containing coding errors that would otherwise lead to the production of non-functional or potentially deleterious proteins. NMD occurs in yeasts, plants, flies, worms, mice, and humans. In humans, NMD affects the etiology of genetic disorders by affecting the expression of genes that carry disease-causing mutations. Besides quality assurance, NMD plays another role in gene expression by controlling the abundance of hundreds of normal mRNAs that are devoid of coding errors. In this paper, the authors used DNA arrays to monitor the relative decay rates of all mRNAs in budding yeast and found a subset where decay rates were dependent on NMD. Many of the corresponding proteins perform related functional roles affecting both the structure and behavior of chromosomes and the structure and integrity of the cell surface.

## Introduction

In eukaryotes, a conserved RNA surveillance system that depends on nonsense-mediated mRNA decay (NMD) causes rapid degradation of transcripts that contain a premature termination codon [1]. RNA surveillance prevents the accumulation of potentially deleterious truncated proteins. Because NMD is linked to mRNA splicing in mammals, it has been suggested that the NMD pathway may have co-evolved with introns and may play an important role in determining the positions of introns in mammalian genes [2]. More importantly, NMD has a direct impact on the etiology of human genetic disorders, where about 25% of all mutations causing genetic disorders and inherited cancers are chain termination mutations that most likely trigger NMD [3].

In the yeast *Saccharomyces cerevisiae,* three *trans-*acting factors are required for NMD, including Upf1p, Upf2p, and Upf3p [4–7]. Orthologs of these genes have been found in all branches of the eukaryotes, including humans [1]. The Upf proteins and translation termination factors eRF1 *(SUP45)* and eRF3 *(SUP35)* form a surveillance complex leading to premature termination of translation, 5′ decapping, and poly(A)-independent degradation of the nonsense-containing transcript [8–12]. Genomic studies have shown that the surveillance complex also performs functions in the regulation of gene expression. NMD affects the accumulation of ~600 naturally occurring RNAs [13,14]. The impact of NMD on the global expression profile has been shown to have physiological consequences. Nmd^−^ strains have alterations in chromosome structure, including a reduction in the average number of hexameric repeats at telomeres, an impairment of gene silencing in silent regions of DNA near telomeres, and altered function of kinetochores. Nmd^−^ strains also grow at reduced rates on some non-fermentable carbon sources [15–18].

In *upf^−^* mutants defective for NMD, changes in the accumulation of NMD-sensitive transcripts are sometimes associated with a change in the rate of RNA decay. Transcripts that behave in this manner are referred to as direct targets of NMD. Two mechanisms for the selective, direct targeting of naturally occurring, error-free transcripts for accelerated decay have been described, including the presence of an upstream open reading frame (uORF) that triggers translation termination in the 5′ leader of the *CPA1* transcript [19] and out-of-frame premature translation termination following improper translation initiation of the *SPT10* transcript [20]. Other mechanisms must exist since uORFs and out-of-frame translation initiation are not responsible for the NMD-sensitive half-life of *PPR1* mRNA [21].

NMD-sensitive changes in accumulation are not always accompanied by altered decay rates. These transcripts are referred to as indirect targets of NMD. A model accounting for direct and indirect targets was proposed on the basis that the mRNAs coding for several transcription factors were shown to be sensitive to NMD. It was anticipated that changes in the abundance of the transcription factors might cause changes in the rates of transcription of downstream-regulated genes, which could indirectly affect the abundance of the downstream-regulated transcripts [14]. Examples supporting the model have been reported, including regulation of *URA3* and *HHF2* by transcriptional activators Ppr1p [6,21] and Spt10p [20], respectively. The *URA3* and *HFF2* mRNAs are indirect targets with NMD-insensitive decay rates, whereas the *PPR1* and *SPT10* mRNAs are direct targets with NMD-sensitive decay rates. Indirect targets might also arise by other mechanisms. For example, NMD-sensitive transcripts coding for chromatin remodeling factors might affect rates of local transcription through changes in chromatin structure [16].

No systematic genome-wide identification of the direct and indirect targets of NMD has been undertaken and only a small number of NMD-sensitive RNAs have been examined for changes in mRNA decay rates [16,17,20,22,23]. To extend knowledge about direct and indirect targeting, we developed a computational approach to estimate the relative proportions of direct and indirect targets among NMD-sensitive transcripts. The experimental design resembles a typical mRNA half-life experiment where transcription is blocked and the rate of disappearance of a single transcript is monitored by Northern hybridization, except that high-density arrays were used for hybridization so that all transcripts could be monitored simultaneously. By combining established and novel statistical methods, global predictions were made regarding the number of transcripts that are direct targets of NMD. A statistical parameter (*p*-value) was calculated for each NMD-sensitive RNA summarizing the likelihood of being a direct target. Representative protein-coding transcripts were analyzed by conventional methods to verify predicted changes in abundance and decay, to assess rates of false discoveries and misclassifications, to assess targeting mechanisms and their relative frequencies of use, and to begin describing the functional relationships among NMD-sensitive targets. Overall, the results provide a paradigm for dynamic analysis of RNA decay pathways and the regulatory networks that control them, in any organism (including humans) where the genome sequence and the means for inhibiting transcription of cells cultured in vitro are available.

## Results

### Global Estimation of Relative RNA Decay Rates in Nmd^+^ and Nmd^−^ Strains

A two-step approach was used to investigate the global impact of NMD on gene expression. First, RNAs that change in abundance in the absence of a functional NMD pathway were identified. Our studies focus on these RNAs because the biological impact of NMD is most likely mediated through corresponding changes in protein abundance. Some NMD-sensitive RNAs might be excluded using this approach since compensatory mechanisms could potentially restore normal RNA levels by counteracting NMD-mediated changes in RNA half-life. Such RNAs, if they exist, were ignored as being biologically irrelevant with respect to NMD because there would be no effect on the abundance of the protein products.

To find those RNAs expressed at different levels in Nmd^−^ *(upf1-Δ)* and Nmd^+^ *(UPF1)* strains (Table S1), image files of Affymetrix YG-S98 arrays hybridized to bulk cRNA probes from the two strains were pre-processed using robust multi-chip average [24] and then compared by SAM (significance analysis of microarrays) [25]. Using a 1.5-fold minimum threshold for the change in abundance and a 5% false discovery rate (FDR) as the statistical cutoff, 625 probe sets corresponding to 616 RNAs were identified. 607 probe sets (598 RNAs) were detected at elevated levels and 18 at reduced levels in the Nmd^−^ strain (Tables S2 and S3, respectively). The experiments described below focus on the 607 probe sets corresponding to the RNAs with elevated levels. The majority of these probe sets were also identified as targets of NMD in two previous studies [13,14,17].

To further characterize the NMD-sensitive RNAs, levels of accumulation were monitored using YG-S98 arrays at 16 time points across a 1-h window following inhibition of transcription with thiolutin. The kinetics of disappearance of RNAs synthesized before inhibition of transcription provide an estimate of the relative rates of decay. We asked which of the 607 probe sets corresponding to RNAs that were differentially expressed in Nmd^−^ and Nmd^+^ strains also had differential rates of decay, making it possible to distinguish direct from indirect targets.

Estimating decay rates on a global scale required development of bioinformatic methods, including selection of a kinetic model for decay to be used for data fitting and methods of normalization to allow direct comparison of data from each time interval following inhibition of transcription. Text S1 and Tables S4, S5, and S6 describe alternative decay models, the criteria for selecting a model for data fitting, and methods for normalization of array data. A non-first-order decay model was selected that is described by the equation *Y* = *c*_0_ + *β_0_* exp*(β_1_X) + ɛ,* where β_1_, the decay rate parameter, is inversely related to RNA half-life *(t*_1/2_ *= −*ln2 / *β_1_)_._* Graphs describing the kinetics of RNA decay were generated using this model. For each probe set, we fit the data using a grid search algorithm and estimated model parameters to test the null hypothesis that RNA decay rates are equal between Nmd^+^ and Nmd^−^ strains. Based on a likelihood ratio test statistic, *p*-values were assigned to each probe set.

**Figure 1:**
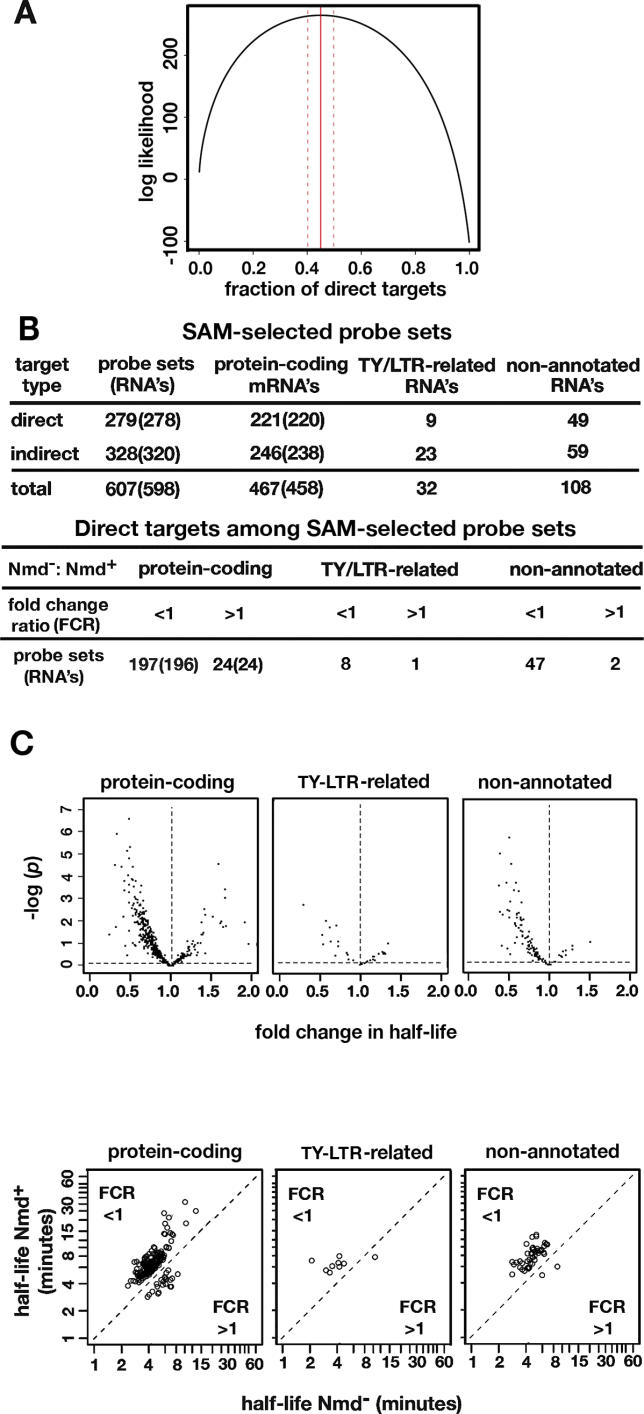
Classification of the NMD-Sensitive RNAs (A) Maximum likelihood estimate of the proportion of direct targets of NMD. (B) 607 probe sets identified by SAM as NMD targets that increase in abundance in the Nmd^−^ strain fell into three groups: protein-coding mRNAs, RNAs related to transposable TY elements and solo LTRs, and non-annotated RNAs corresponding to genomic sequences identified by SAGE tags. In each column the number of probe sets is shown. The number of protein-coding RNAs is shown in parentheses. Some RNAs are represented by more than one probe set. Direct targets have NMD-sensitive decay rates and indirect targets have NMD-insensitive decay rates. For each of the three groups, the predicted direct targets sub-divide into those with an FCR <1 or >1, determined by dividing the predicted fold change in half-life in the Nmd^−^ strain by the fold change in half-life in the Nmd^+^ strain. (C) The top panels show the relationship between FCR and the *p*-value. The bottom panels show the half-lives of predicted direct targets in the Nmd^+^ strain plotted against the half-lives in the Nmd^−^ strain.

In addition to this approach, a novel application of maximum likelihood was developed to predict the proportion of direct targets. We found that 45% ± 5% of the 607 SAM-selected probe sets are likely to be direct targets with altered decay rates (Figure 1). The prediction is based on the following reasoning. We defined the parameter *δ* equal to 1 if the transcript is a direct target and 0 if the transcript is an indirect target, and denote *f* ^(*1*)^ (*p*) and *f* ^(*0*)^ (*p*) to be the probability density function (pdf) of a *p*-value. For hypothesis testing, *f* ^(0)^ (*p*) ≡ 1 for 0 ≤ *p* ≤ 1. Since *f* ^(1)^ (*p*) is an unknown function, the pdf with linear interpolation of the *p*-values for 14 mRNAs shown to be direct targets by conventional half-life experiments (Table 1) was used to estimate *f* ^(1)^ (*p*), where the estimated pdf is denoted as fˆ^(1)^(*p*).

**Table 1:**
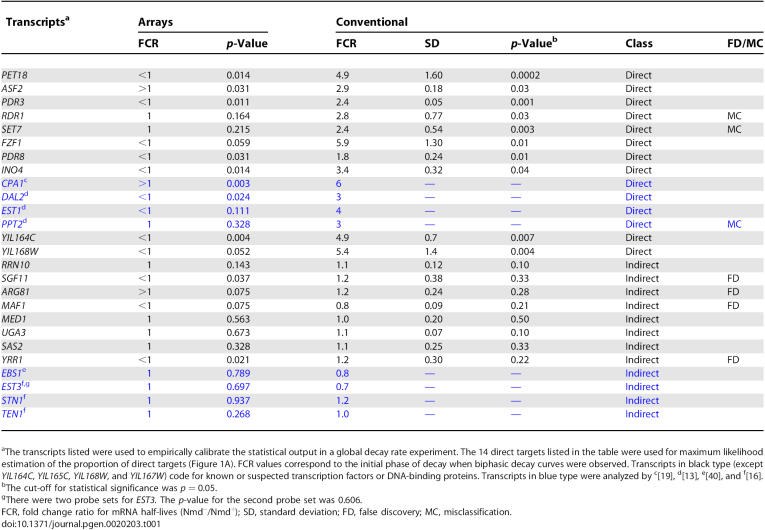
NMD-Sensitive Protein-Coding Transcripts

Assuming Pr(δ = 1) = η, the marginal pdf of *p* is 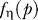 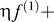 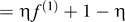 , and its plug-in estimation is 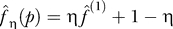 . Based on this, the estimated log-likelihood function of η is:

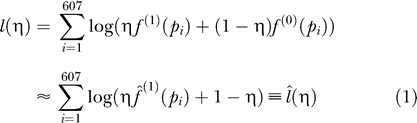



In Figure 1A, 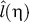 reaches its maximum when η = 0.450, which is the estimated proportion of direct targets. According to the asymptotic theory of maximum likelihood estimation, 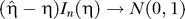 , where *n* is the sample size (in our case *n* = 607), and *I_n_*(η) is Fisher's information, then:

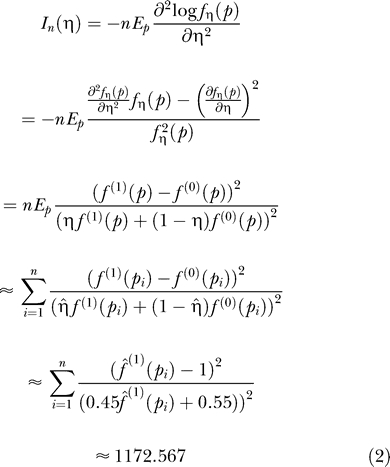



Since 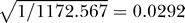 , then 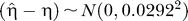 . Therefore, the 95% confidence interval of η is 0.450 ± 1.96 × 0.0292 ≈ (0.393,0.507). This means that 95% of the intervals derived by this method will contain the true proportion of direct targets. The reliability of this approach was verified by bootstrapping (Materials and Methods, Figure S1).

The 607 SAM-selected probe sets were divided into three groups: protein-coding RNAs, RNAs related to yeast transposable (TY) elements and the long terminal repeats (LTRs) found alone or in association with intact TY elements, and non-annotated RNAs corresponding to the locations of serial analysis of gene expression (SAGE) tags that are diagnostic of expressed sequences in the yeast genome (Figure 1B). RNAs from each sub-class were assigned to one of two categories: direct targets that have altered decay rates and indirect targets that have unaltered decay rates. The assignments were based on a comparison of the kinetics of RNA decay in Nmd^−^ and Nmd^+^ strains, where the reliability of each assignment was based on a *p*-value indicating the statistical likelihood of being a direct target.

### Comparison of Global and Conventional Half-Life Analyses

In the protein-coding mRNA group identified above, 18 RNAs were analyzed by conventional half-life experiments (Tables 1 and S7). The RNAs were part of a larger subset used to calibrate the statistical output in the global decay rate experiment (Materials and Methods and Text S1). For these and other RNAs mentioned below, the accession numbers for the Entrez Gene database are included at the end of the text and also summarized in Table S8. SAM-selected RNAs that accumulated to a higher level due to inactivation of NMD were expected to be more stable in the Nmd^−^ mutant strain than in the Nmd^+^ strain. Changes in decay rates were expressed as fold change ratios (FCRs) determined by dividing the half-life in the mutant strain by the half-life in the wild-type strain. The algorithm in the global decay rate experiment included statistical tools to determine whether the FCR was significantly different from 1 without regard to the direction of change. When the FCR was significantly <1 or >1, the RNA was classified as a direct target of NMD with an altered half-life.

**Figure 2:**
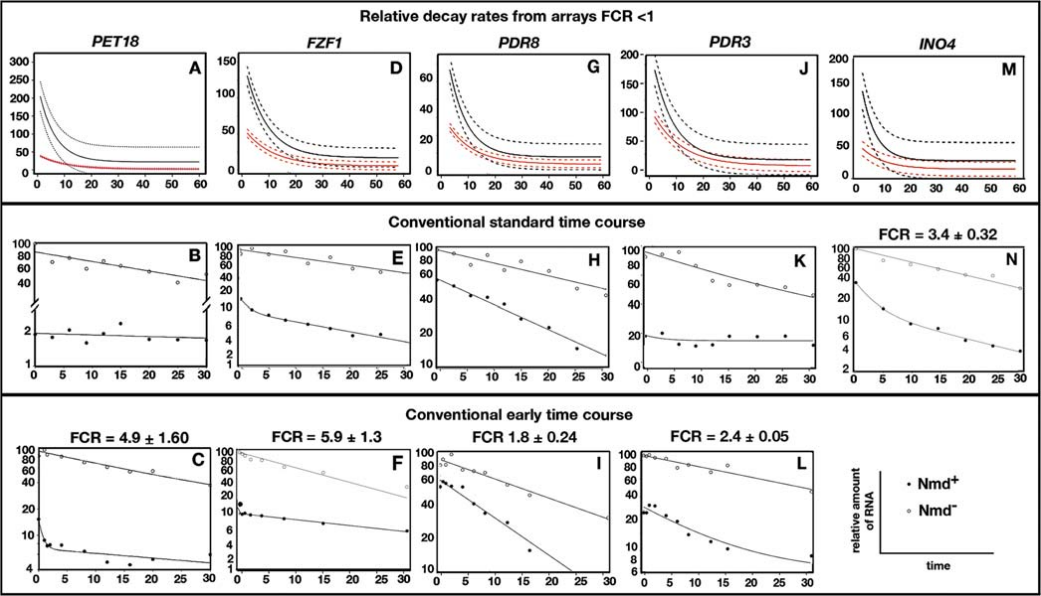
FCR for mRNA Half-Lives Representative protein-coding transcripts where FCR was <1 in a global decay rate experiment were re-analyzed in conventional half-life experiments. (A, D, G, J, and M) illustrate the relative kinetics of mRNA decay from array data (*n* = 3) using a curve-fitting algorithm (see Text S1) (red solid lines, Nmd^+^; black solid lines, Nmd^−^; dashed lines, 90% confidence intervals). (B, E, H, K, and N) show conventional half-life experiments using a standard time course. (C, F, I, and L) show conventional half-life experiments using clustered early time points. For each mRNA, decay curves are arranged vertically. RNA levels were monitored by Northern blotting after inhibition of transcription with thiolutin. The FCR and standard error are shown for each mRNA. In this and subsequent figures, the FCR values correspond to the initial phase of decay when biphasic decay curves were observed. The results are summarized in Table 1.

Direct targets of NMD that had higher accumulation levels were expected to have slower decay rates in Nmd^−^ strains with an FCR >1. However, 91% had FCRs <1 (Figure 1B and 1C). To gain an understanding of the underlying cause of FCRs that were reversed relative to expectation, five representative protein-coding transcripts where FCRs were <1 in the global decay rate experiment (top panels of Figure 2) were re-analyzed in conventional half-life experiments. The half-lives were assayed using a distribution of time points similar to that used in the global experiment (conventional standard time course, middle panels of Figure 2) and an alternate distribution including clustered points taken soon after inhibition of transcription (conventional early time course, bottom panels of Figure 2).

Using a standard time course, *PET18* mRNA had a 3-fold shorter half-life in the Nmd^−^ mutant compared to the Nmd^+^ strain (Figure 2B), which was consistent with FCR <1 in the global experiment. However, when an early time course was used, biphasic kinetics was observed with a rapid initial phase and a slower second phase (Figure 2C). The half-life in the initial decay phase was 3 min in the Nmd^+^ and 20 min in the Nmd^−^ strain. The FCR was 4.9 ± 1.6 (*n* = 3, *p* = 0.0002, Table 1). Using a cutoff for statistical significance of *p* = 0.05, the results indicate that *PET18* is a direct target with an initial decay rate that is NMD-sensitive. The anomalous FCR value generated by the curve-fitting algorithm in the global experiment was a consequence of the paucity of early time points needed to accurately measure the early phase of decay. Despite this, the algorithm correctly identified *PET18* as having an altered decay rate because it was designed to ignore the direction of change.

Similar results were obtained when *FZF1, PDR8, PDR3,* and *INO4* mRNAs were analyzed by conventional half-life experiments. In a representative early time-course experiment for *FZF1,* biphasic decay was observed with an initial half-life of 2 min in the Nmd^+^ strain and 12 min in the Nmd^−^ strain (Figure 2F). The FCR was 5.9 ± 1.3 (*n* = 4, *p* = 0.01). For *PDR8,* a single decay phase was observed with a half-life of 9 min in the Nmd^+^ strain and 18 min in the Nmd^−^ strain (Figure 2I). The FCR was 1.8 ± 0.24 (*n* = 3, *p* = 0.01). For *PDR3,* the half-life was 11 min in the Nmd^+^ strain and 27 min in the Nmd^−^ strain (Figure 2L). The FCR was 2.4 ± 0.05 (*n* = 3, *p* = 0.001). Biphasic decay was observed for *INO4* mRNA using a standard time course (Figure 2N). The half-life was 5 min in the Nmd^+^ strain and 18 min in the Nmd^−^ strain. The FCR was 3.4 ± 0.32 (*n* = 3, *p* = 0.04).

### False Discoveries, Misclassifications, and Confirmations

In the global decay rate experiment, the statistical FDR for transcripts predicted to be direct targets was set at 0.30 according to the Benjamini-Hochberg procedure [26]. Thus, incorrect assignments where predicted direct targets were actually indirect targets could occur up to a maximum frequency of 30%. Misclassifications of indirect targets as direct targets in the global experiment could also occur, but the frequency was reduced when a relatively high FDR was selected (see Table S9 for further details on FDR analysis).

**Figure 3:**
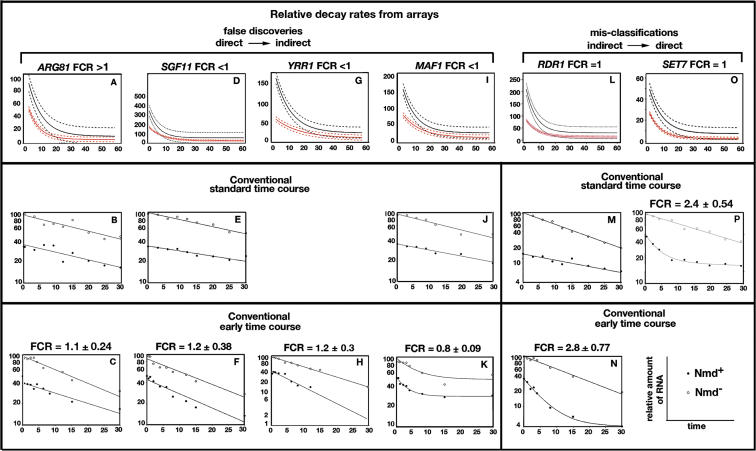
False Discoveries and Misclassifications FDRs and misclassifications were empirically tested by conventional half-life experiments. Representative RNA decay data for four false discoveries and two misclassifications (*n* = 3) are presented in the same format as described in Figure 2. The results are summarized in Table 1.

Four examples of false discoveries were identified (Figure 3). *ARG81* mRNA was predicted to be a direct target with an FCR >1 (Figure 3A), whereas *SGF11, YRR1,* and *MAF1* were predicted to be direct targets with FCRs <1 (Figure 3D, 3G, and 3I). In representative conventional experiments, the half-lives in Nmd^+^ and Nmd^−^ strains, respectively, were: *ARG81,* 20 and 15 min (Figure 3C) (FCR = 1.2 ± 0.24, *n* = 4, *p* = 0.28); *SGF11,* 13 and 16 min (Figure 3F) (FCR = 1.2 ± 0.38, *n* = 3, *p* = 0.33); *YRR1,* 7 and 10 min (Figure 3H) (FCR = 1.2 ± 0.3, *n* = 3, *p* = 0.22); and *MAF1,* 6 and 8 min (Figure 3K) (FCR = 0.8 ± 0.09, *n* = 3, *p* = 0.21). Using *p* = 0.05 as the cutoff, all four transcripts had FCRs that were statistically indistinguishable from a value of 1. Despite the global prediction of being direct targets, they were falsely discovered and were actually indirect targets.

Conventional half-life experiments revealed two misclassified transcripts, *RDR1* and *SET7* (Figure 3)*.* Both were predicted to be indirect targets (FCR = 1) in the global experiment (Figure 3L and 3O), but proved to be direct targets. In Nmd^+^ and Nmd^−^ strains, respectively, the *RDR1* half-life was 4 and 12 min (Figure 3N), and the *SET7* half-life was 7 and 22 min, respectively (Figure 3P). Another transcript, *PPT2,* was also considered misclassified based on previously published data [13]. The conventional FCRs were 2.8 ± 0.77 (*n* = 3, *p* = 0.03) for *RDR1* and 2.4 ± 0.54 (*n* = 4, *p* = 0.003) for *SET7.*

**Figure 4:**
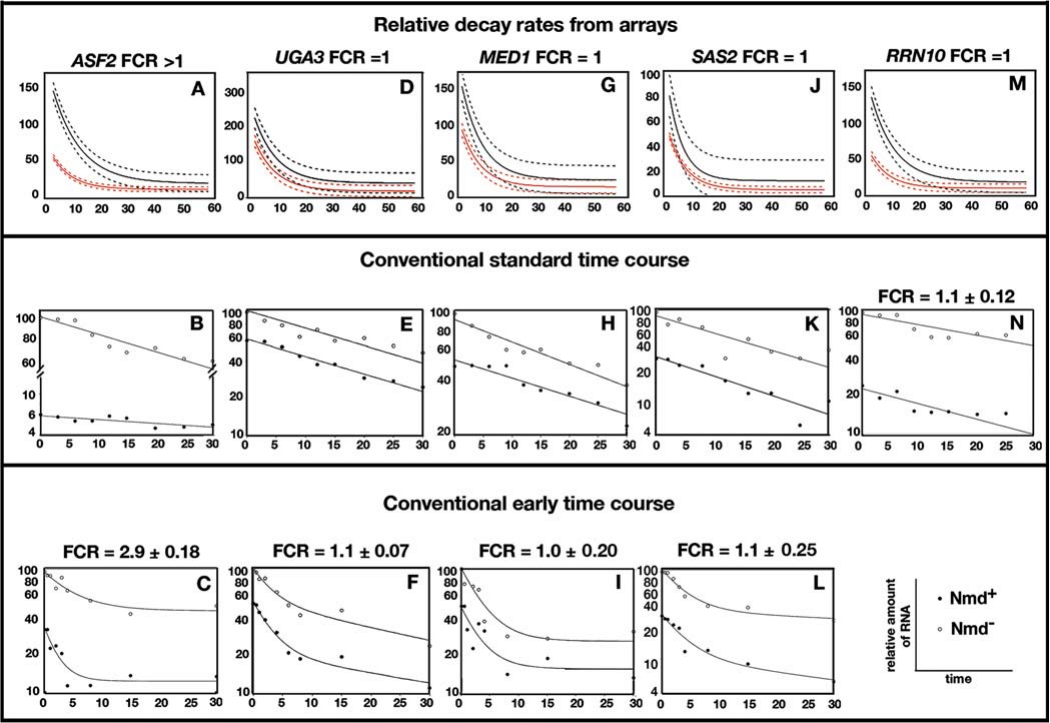
Confirmation of Global Predictions Representative RNA decay data for five transcripts are shown (*n* = 3) where global predictions were borne out by conventional half-life experiments. The format is identical to Figure 2. The results are summarized in Table 1.

Global predictions for five additional transcripts were confirmed by conventional half-life experiments (Figure 4); including the direct target *ASF2* and four indirect targets, *UGA3, MED1, SAS2,* and *RRN10.* In representative conventional half-life experiments, the half-lives in Nmd^+^ and Nmd^−^ strains, respectively, were: *ASF2,* 4 and 11 min (Figure 4C) (FCR = 2.9 ± 0.18, *n* = 3, *p* = 0.03); *UGA3,* 5 and 6 min (Figure 4F) (FCR = 1.1 ± 0.07, *n* = 3, *p* = 0.1); *MED1,* 3 and 4 min (Figure 4I) (FCR = 1.0 ± 0.2, *n* = 3, *p* = 0.5); *SAS2,* 6 and 8 min (Figure 4L) (FCR = 1.1 ± 0.25, *n* = 3, *p* = 0.33); and *RRN10,* 22 and 22 min (Figure 4N) (FCR = 1.1 ± 0.12, *n* = 3, *p* = 0.1). Using a cutoff of *p* = 0.05, *ASF2* was confirmed as a direct target and the others as indirect targets.

Rates of false discovery and misclassification are inversely related to each other. High FDRs correspond to low misclassification rates. Setting the FDR at the relatively high value of 30% accomplished the goal of maximizing the number of actual direct targets among predicted direct targets. Using this approach, the empirical data indicate that the actual FDR was 4/13 (31%) whereas the misclassification rate was 3/14 (21%). Since false discoveries and misclassifications occurred at similar frequencies, the predicted frequencies of direct and indirect targets relative in the global experiment are likely to be close to the actual frequencies.

Direct and indirect targets were also examined to see if members of the two groups could be distinguished by comparing the magnitudes of change in RNA abundance caused by the inactivation of NMD. When protein-coding transcripts were examined, the average FCR based on RNA abundance at *t* = 0 were 2.6 ± 1.2 for direct targets (221 probe sets, 220 transcripts) and 2.4 ± 0.9 for indirect targets (246 probe sets, 238 transcripts). The averages were statistically indistinguishable, indicating that the average magnitudes of changes in abundance were similar for direct and indirect targets.

### Changes in Decay Rates Are Unaffected by Thiolutin and Mediated through 5′ Degradation

**Figure 5:**
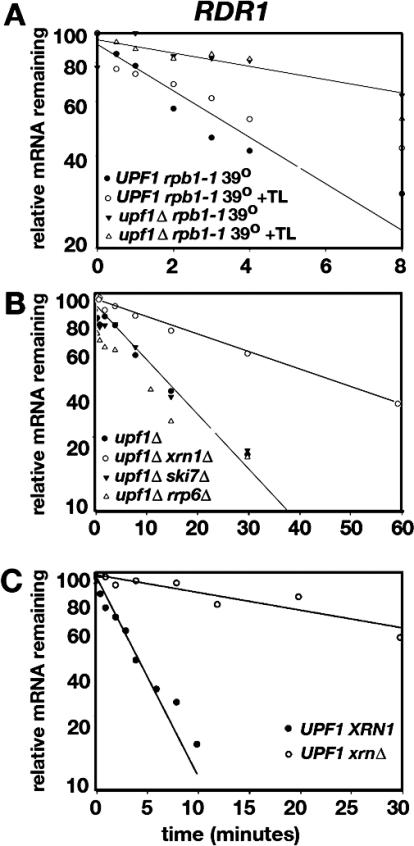
Degradation of Natural RNA Targets by NMD (A) Thiolutin has no effect on mRNA decay. *RDR1* mRNA decay was examined using two methods to inhibit transcription: addition of thiolutin and exposure of cells carrying *rpb1–1* (impaired RNAP II) to a restrictive growth temperature of 39 °C [70]. Transcription was inhibited in *UPF1* and *upf1Δ* cells by temperature-shift alone or by temperature-shift combined with the addition of thiolutin. A representative experiment (*n* = 3) is shown where RNA levels were determined by Northern blotting. (B) A representative experiment (*n* = 3) is shown where the kinetics of *RDR1* mRNA decay were monitored after addition of thiolutin in *upf1Δ* cells carrying mutations that block decay from the 5′ end *(xrn1Δ)* or the 3′ end *(ski7Δ* or *rrp6Δ).* (C) A representative experiment (*n* = 3) is shown where the kinetics of *RDR1* mRNA decay was monitored after addition of thiolutin in *UPF1* cells carrying *XRN1* or *xrn1Δ.*

Experiments were performed to test whether thiolutin, which acts as a as a metal chelating agent [27], directly affects RNA decay rates separately from effects on transcription (Figure 5). Since thiolutin was used in both global and conventional decay rate experiments to inhibit transcription, experiments were performed to test whether thiolutin induces accelerated decay. The half-life of *RDR1* mRNA, a direct target of NMD (Figure 3, Table 1), was assayed following a double shut-off of transcription achieved by adding thiolutin immediately after temperature shift in strains that carry *rpb1–1,* a temperature-sensitive allele of the large subunit of RNAPII (Figure 5A). If the effects of thiolutin are restricted to inhibition of transcription, then decay rates measured after double transcriptional shut-off should resemble decay rates after temperature shift alone.

The results of the double shut-off experiment showed that thiolutin inhibits transcription without independently affecting mRNA decay rates. In *UPF1* (Nmd^+^) strains, the half-life of *RDR1* mRNA was 5.2 ± 0.3 min in the presence of thiolutin and 4.4 ± 2.7 min in the absence of thiolutin. Using *p* = 0.05 as the cutoff, the difference was statistically insignificant (*n* = 3, *p* = 0.34). In *upf1Δ* strains, the half-life of *RDR1* mRNA was 14.5 ± 3.5 min in the presence of thiolutin and 12.9 ± 3.7 min in the absence of thiolutin. This difference was also statistically insignificant (*n* = 3, *p* = 0.45). When the same experiments were performed with *INO4* mRNA, similar results were obtained (unpublished data).

Nonsense transcripts are degraded primarily by a 5′ decay pathway requiring exoribonuclease Xrn1p [12]. An experiment was performed showing that natural targets of NMD are also degraded through the 5′ decay pathway. *RDR1* mRNA was examined in a double mutant strain carrying *upf1Δxrn*Δ defective for 5′ decay, a double mutant *upf1Δ ski7Δ* defective for cytoplasmic 3′ decay [28], and a double mutant *upf1Δ rrp6Δ* defective for nuclear 3′ decay [29]. In the experiment shown in Figure 5B, the *RDR1* mRNA half-life was 16 ± 3.1 min in the *upf1Δ* single mutant, 18 ± 3.3 min in the *upf1Δ ski7Δ* double mutant, and 15 ± 2.1 min in the *upf1Δ rrp6Δ* double mutant. Compared to the *upf1Δ* single mutant, the FCRs were 1.2 ± 0.4 (*n* = 3, *p* = 0.27) and 0.9 ± 0.1 (*n* = 3, *p* = 0.15). The results suggest that in the absence of NMD, the *RDR1* mRNA is not degraded by the 3′ to 5′ cytoplasmic pathway or the nuclear 3′ degradation system. However, the *RDR1* half-life in the *upf1Δ xrn1Δ* double mutant was 42 ± 1.3 min. Compared to the single mutant, the FCR was 2.7 ± 0.6 (*n* = 3, *p* = 0.02), indicating that *RDR1* mRNA is degraded by the 5′ decay pathway.

To test whether degradation of *RDR1* mRNA occurs via 5′ decay when the NMD pathway is functional, the abundance and half-life were compared in wild-type *UPF1 XRN1* and *UPF1 xrn1Δ* strains. The FCR for the relative abundance of *RDR1* mRNA at *t* = 0 was 8.9 ± 1.3 (*n* = 3, *p* = 0.00009). In the experiment shown in Figure 5C, the half-life was 3.3 ± 0.6 min in the *UPF1 XRN1* strain and 34 ± 4 min in the *UPF1 xrn1Δ* strain. The FCR for the half-life was 10.1 ± 2.2 (*n* = 3, *p* = 0.009). These results demonstrate that the degradation of *RDR1* mRNA by NMD depends on a functional 5′ decay pathway.

### Targeting of Disabled Open Reading Frames

Several types of disabled open reading frames, designated dORFs, have been described in the yeast genome [30,31]. dORFs consist of adjacent in-frame ORFs separated by a short gap or by a naturally occurring stop codon without a gap. The presence of internal stops and gaps could cause defective expression at the level of translation, and for this reason some dORFs could be considered candidates for unprocessed pseudogenes. Overlapping out-of-frame ORFs are also classified as dORFs because co-expression requires a ribosomal frameshift to achieve contiguous translation of both reading frames. Yeast retrotransposons (TY elements) are classified as dORFs consisting of adjacent out-of-frame ORFs. However, programmed frameshifting occurs at low frequency just upstream of the internal stop codon, allowing ribosomes to contiguously translate both reading frames [32,33].

dORFs were identified among NMD-sensitive RNAs, including *YOL163W*/*YOL162W* (separated by 12 bp), *YER039C-A*/*YER039C* (separated by 57 bp), *YIL164C*/*YIL165C* (separated by 9 bp), and *YIL168W*/*YIL167W* (separated only by a stop codon). Probe sets representing TY elements and the LTRs at the ends of the elements were also identified among NMD-sensitive RNAs (Figure 1), suggesting the possibility that internal stop codons in TY elements might trigger NMD.

**Figure 6:**
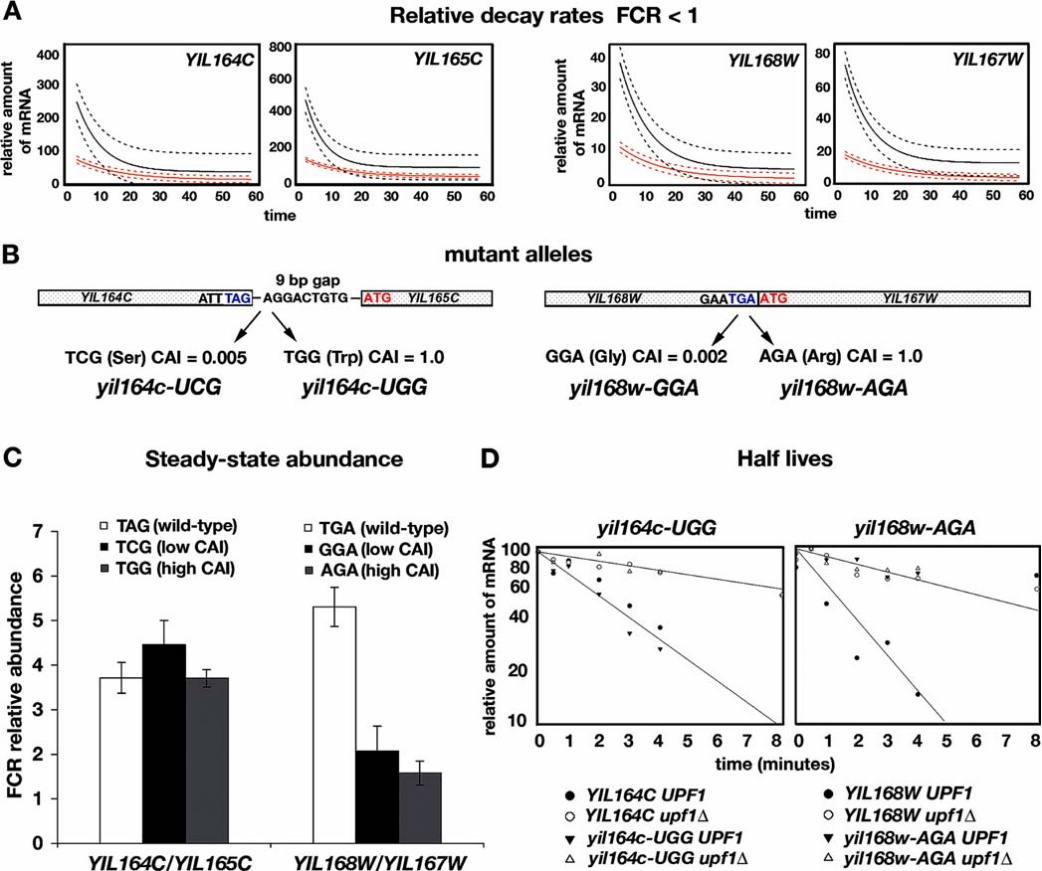
Targeting of dORFs (A) Relative kinetics of decay from array data for two NMD-sensitive dORFs. (B) Organization of the dORFs. The stop codons were changed to rare (low CAI) and commonly used (high CAI) sense codons [77]. (C) Effects of the mutations on RNA abundance expressed as the FCR (*n* = 3). (D) Comparison of half-lives of *yil164-UGG* and *yil168W-AGA* RNA. FCRs were calculated for *n* = 3.

A genetic approach was taken to explore the basis of NMD targeting of dORFs by creating mutations in *YIL164C*/*YIL165C* and *YIL168W*/*YIL167W* (Figure 6). Both were predicted to be direct targets of NMD in the global decay rate experiment (Figure 6A), and *YIL164C/YIL165C* was shown previously to be a direct target [13]. We tested the hypothesis that the stop codon between ORFs in each dORF pair triggers NMD similar to the way nonsense mutations trigger NMD. In each dORF, two different substitutions were created to change the natural stop codons to sense codons (Figure 6B). The mutants were then assayed to determine the FCRs for abundance and half-life in Nmd^−^ and Nmd^+^ strains.

A nearly 4-fold change in the abundance of *YIL164C* was observed in the absence of NMD (Figure 6C), but the magnitude of change was the same when the UAG stop codon at the end of *YIL164C* was changed to UCG or UGG. The FCRs (*n* = 3) for the abundance of *YIL164C, yil164c-UCG,* and *yil164c-UGG* were 3.7 ± 0.4, 4.5 ± 0.5, and 3.7 ± 0.2, respectively. In the experiment shown in Figure 6D, the half-lives of *YIL64C* and *yil64c-UGG* mRNAs were statistically the same in Nmd^+^ strains (3.1 ± 0.4 and 4.3 ± 1.4 min, respectively), whereas both mRNAs had significantly longer half-lives in Nmd^−^ strains (15.6 ± 2.9 and 15.9 ± 2.7 min, respectively). The FCRs for the *YIL164C* and *yil164c-UGG* RNA half-lives were 4.9 ± 0.7 (*n* = 3, *p* = 0.007) and 4.1 ± 1.6 (*n* = 3, *p* = 0.018), respectively. Using *p* = 0.05 as the cutoff, the results indicate that altering the stop codon to a sense codon failed to abolish NMD targeting. The results indicate that the internal stop codon could trigger NMD, but changing the stop codon to a sense codon is not sufficient by itself to abolish the effects of NMD. Another mechanism acting independently of the internal stop codon must be responsible for targeting, possibly a uORF in the 5′ leader (Table 2). Results described below indicate that uORFs can target mRNAs for NMD.

**Table 2:**
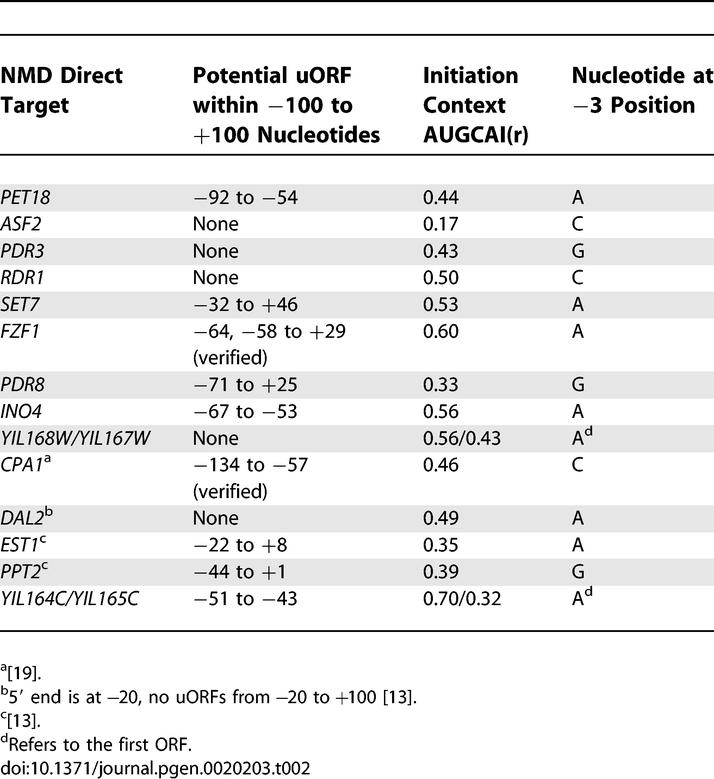
Evaluation of the Likelihood of Direct Targeting by Known Mechanisms

In contrast, changing the UGA stop codon at the end of *YIL168W* to a sense codon abolished targeting. In Nmd^−^ strains, the abundance of *YIL168W* mRNA increased by more than 5-fold (Figure 6C). The difference in abundance was substantially reduced when the stop codon was replaced with a sense codon. The FCRs at *n* = 3 for the abundance of *YIL168W, yil168w-GGA,* and *yil168w-AGA* were 5.3 ± 0.4, 2.1 ± 0.6, and 1.6 ± 0.3, respectively. The half-life of *YIL168W* was 1.1 ± 0.4 and 5.2 ± 0.5 min in Nmd^+^ and Nmd^−^ strains, respectively (Figure 6D). The half-life of *yil168w-AGA* was 5.5 ± 1.5 and 6.9 ± 0.4 min in Nmd^+^ and Nmd^−^ strains, respectively. The FCRs for the half-lives of *YIL168W* and *yil168w-AGA* RNAs were 5.0 ± 1.4 (*n* = 3, *p* = 0.004) and 1.3 ± 0.3 (*n* = 3, *p* = 0.15), respectively. Using *p* = 0.05 as the cutoff, the results indicate that altering the stop codon to a sense codon abolishes targeting. Therefore, the stop codon targets *YIL168W* for NMD.

Our results and the results of others [13] suggest that transcripts produced from TY elements are sensitive to NMD. However, evaluating the basis of the NMD-sensitivity is complicated for two reasons. Different classes of elements show significant sequence similarity causing the probe sets tiled on arrays to exhibit extensive cross-hybridization. Also, some of the TY-related probe sets anneal to LTRs, but the LTRs are found not only at the ends of intact TY elements but also as solo LTRs that lack an adjacent intact element.

To determine whether full-length TY transcripts containing adjacent out-of-frame dORFs are targeted by NMD due to the presence of an internal stop codon, steady-state Northern blots were analyzed using a probe that uniquely hybridizes to transcripts produced from the two known genomic copies of TY3 (see Materials and Methods). When TY3 RNA levels were compared in Nmd^−^ and Nmd^+^ strains, the FCR with respect to abundance was 1.1 ± 0.2 (*n* = 4, *p* = 0.4), indicating that the accumulation of TY3 mRNA is not affected by NMD. Transcripts produced from the two identical genomic copies of TY3 are therefore insensitive to NMD. Furthermore, the internal stop codon in TY3 transcripts fails to trigger NMD. Although it is not yet clear why TY-specific probe sets are heavily represented among NMD-sensitive targets, some possibilities are presented in the discussion based on the observation that most of the NMD-sensitive TY-related probe sets correspond to LTRs.

### Targeting through Translation of uORFs

**Figure 7:**
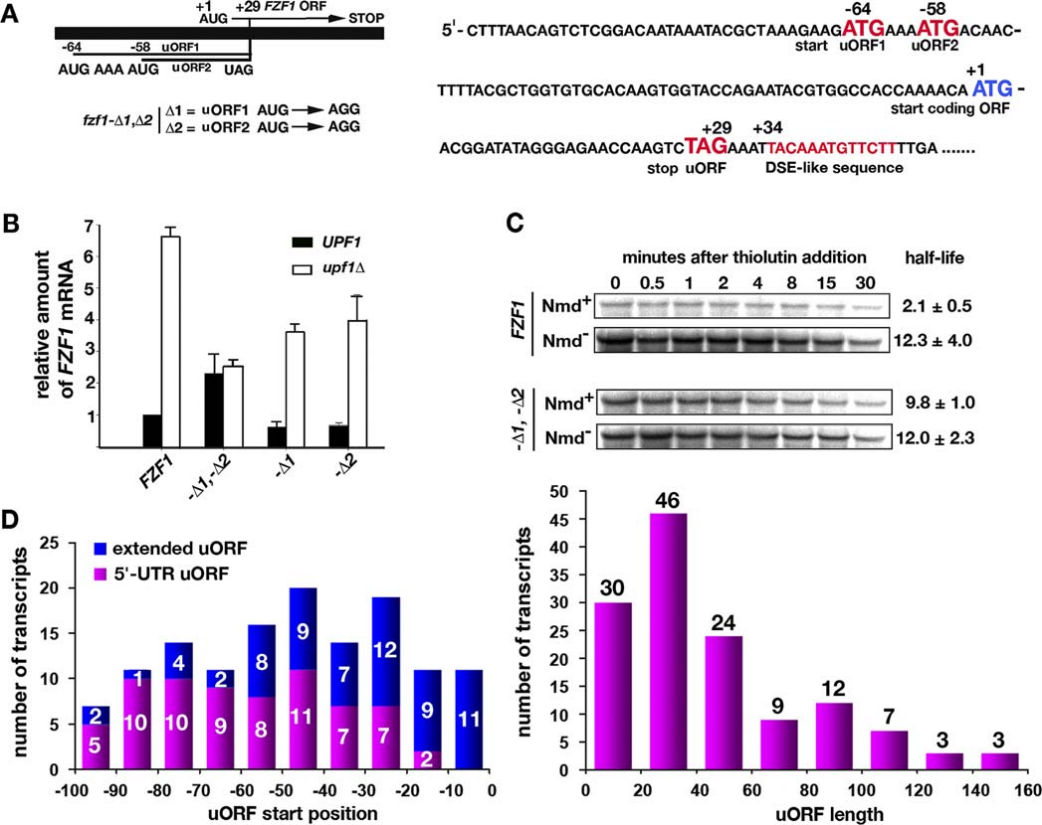
Targeting through uORFs (A) Two uORF start codons are present in the *FZF1* sequence. The uORF stop codon is located upstream of the start codon of the coding ORF in the −1 reading frame. The position of a sequence resembling a downstream element reported to be required for NMD [78,79] is shown. uORF start codons were changed to AGG sense codons. DSE, downstream element. (B) Steady-state RNA levels for *FZF1, fzf1-Δ1, fzf1-Δ2,* and *fzf1-Δ1,-Δ2* determined by Northern blotting. (C) Comparison of *FZF1* and *fzf1-Δ1,-Δ2* RNA half-lives in Nmd^+^ and Nmd^−^ strains. Half-lives, FCRs, and *p*-values were calculated for *n* = 5. (D) Potential uORFs with end points between −100 and +100 nucleotides of predicted direct targets as a function of start position and total length. Left: upper and lower numbers refer to uORFs that end downstream and upstream of the coding ORF start codon, respectively.

NMD-sensitive transcripts were examined with respect to uORFs that might potentially trigger NMD (Figure 7). The sequence upstream of *FZF1* indicates the presence of a uORF with two potential AUG start codons at −64 and −58 followed by an in-frame stop codon ending at position +29 (Figure 7A). To determine whether the *FZF1* uORF plays a role in targeting, the two ATG start codon sequences were changed to AGG singly or together to create the mutant alleles *fzf1-Δ1, fzf1-Δ2,* and *fzf1-Δ1,-Δ2.* The evidence described below indicates that *FZF1* mRNA is targeted for NMD by the uORF.

A 6.6 ± 0.3 (*n* = 3, *p* =.005) -fold change in the relative abundance of the wild-type *FZF1* mRNA was observed when Nmd^+^ and Nmd^−^ strains were compared (Figure 7B), confirming that *FZF1* mRNA is a target of NMD. Fold changes in the levels of the mRNAs produced from the *fzf1-Δ1* and *fzf1-Δ2* single mutant alleles (FCR = 5.9 ± 0.9, *n* = 3, *p* = 0.006; FCR = 5.9 ± 0.6, *n* = 3, *p* = 0.002, respectively) were similar to that found for *FZF1* mRNA, suggesting that these mRNAs were also targets of NMD. However, the fold change in abundance was abolished in the *fzf1-Δ1,-Δ2* double mutant (FCR = 1.1 ± 0.2, *n* = 3, *p* = 0.21), indicating that sensitivity to NMD might be dependent on uORF translation. An additional observation inherent in the data was that overall reductions in mRNA levels were evident in both single and double mutants in both Nmd^+^ and Nmd^−^ strains, suggesting that mutations in the DNA corresponding to the uORF start codons affected mRNA abundance by a second mechanism that was independent of NMD.

To further examine the sensitivity of the double mutant mRNA to NMD, the half-lives of *FZF1* and *fzf1-Δ1,-Δ2* were compared in Nmd^+^ and Nmd^−^ strains. In the representative set of experiments shown in Figure 7C, *FZF1* mRNA had a half-life of 2.1 ± 0.5 min in the Nmd^+^ strain and 12.3 ± 4.0 min in the Nmd^−^ strain. The FCR was 5.9 ± 1.3 (*n* = 3, *p* = 0.005). Using a *p* = 0.05 cutoff, the results confirm that *FZF1* mRNA is a direct target of NMD. The mRNA produced in the *fzf1-Δ1,-Δ2* double mutant had a half-life of 9.8 ± 1.0 min in the Nmd^+^ strain and 12.0 ± 2.3 min in the Nmd^−^ strain. The FCR was 1.2 ± 0.1 (*n* = 3, *p* = 0.121). Using a *p* = 0.05 cutoff, the results show that the NMD-dependent change in half-life observed for wild-type *FZF1* mRNA was abolished in the double mutant. This result supports the conclusion that translation of the uORF is required for the targeting of *FZF1* mRNA. This is the first demonstration of a uORF required for targeting that starts in the 5′ leader and terminates in the coding ORF.

To estimate the number of NMD-sensitive protein-coding mRNAs targeted by uORF translation, an algorithm was used to screen direct targets of NMD for uORFs that start and end between nucleotides −100 and +100 relative to the coding ORF AUG. Uncertainties were inherent in the screen because the locations of most transcription start sites are unknown, but those that have been mapped are typically within 100 nucleotides of the coding ORF AUG. The median length of known 5′-UTRs is 61 nucleotides [34], and >95% of 5′-UTRs are <100 nucleotides in length [35]. 135 of the 220 predicted direct targets of NMD contained a putative uORF (including *CPA1,* which has a leader longer than 100 nucleotides) (Figure 7D). 85 transcripts were devoid of uORFs. 70 transcripts had a uORF that was self-contained in the 5′-UTR (including *CPA1*), and 65 had a uORF that terminated translation out-of-frame in the coding ORF. Potential uORFs for the transcripts listed in Table 1 are described in Table 2. Potential uORFs identified among direct targets of NMD are listed in Table S10.

### Targeting through Out-of-Frame Initiation of Translation

*SPT10* mRNA is targeted for degradation by NMD because translating ribosomes frequently bypass the coding ORF AUG due to a suboptimal context, scan to a downstream AUG in an alternate reading frame, and initiate translation. Termination of translation at an out-of-frame stop codon triggers NMD [20]. Whether this mechanism, referred to as “leaky scanning,” is responsible for targeting other transcripts for NMD remains unverified.

**Figure 8:**
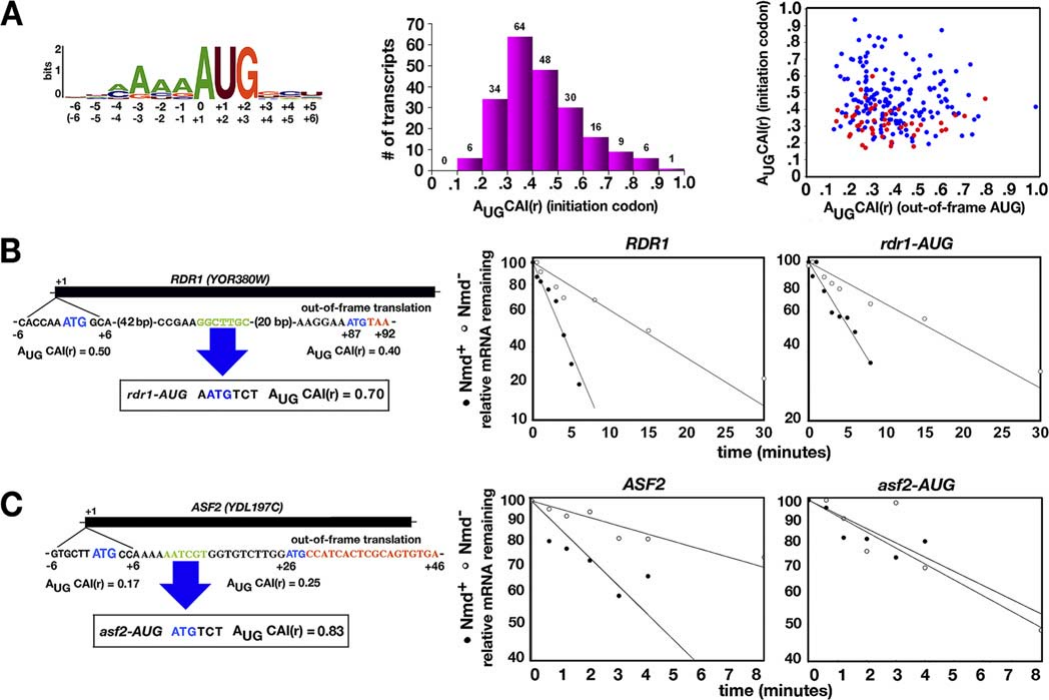
Targeting through Out-of-Frame Initiation of Translation (A) A_UG_CAI(r) scores were calculated to evaluate the influence of context on the efficiency of translation initiation. The bit score indicates relative sequence conservation at a given nucleotide position and the height of nucleotide symbols indicates the frequency of nucleotide use. Standard numbering (parentheses) differs from Web logo numbering. The bar chart shows the distribution of A_UG_CAI(r) scores for protein-coding transcripts that satisfy criteria for targeting by leaky scanning. The dot plot shows the distribution of candidate transcripts where the A_UG_CAI(r) score of the initiator AUG is plotted against the score for the downstream out-of-frame AUG. Red dots correspond to transcripts considered to be likely candidates for leaky scanning based solely on the presence of U or C at the −3 position. (B) Sequence changes in *RDR1* DNA and comparison of *RDR1* and *rdr1-AUG* transcript half-lives in Nmd^+^ and Nmd^−^ strains. (C) Sequence changes in *ASF2* DNA and comparison of *ASF2* and *asf2-AUG* transcript half-lives in Nmd^+^ and Nmd^−^ strains.

The context surrounding an AUG initiation codon is believed to influence the efficiency of translation initiation, especially the nucleotide at the −3 position [36,37]. The context relevant to initiation includes the −6 to +6 nucleotides based on the frequencies of nucleotide usage around the AUG start codons of a set of highly expressed genes. The consensus for optimal initiation is (A/U_−6_)A(A/C)AA(A/C)AUGUC(U/C_+6_) [38]. A codon adaptation index (A_UG_CAI) was previously developed to evaluate all possible context sequences based on frequency of nucleotide usage at each nucleotide position [39]. To test the generality of the leaky scanning model for NMD targeting, we revised the CAI to create an index A_UG_CAI(r) ranging from 0 to 1 based on the weighted contribution of each context position and the relative frequency of nucleotide use at each position (Materials and Methods and Table S11). The relative importance of each context nucleotide is summarized by a Web logo (Figure 8).

A_UG_CAI(r) scores were calculated for predicted direct targets of NMD (Figure 8A, bar graph and Table S12). To visualize the effects of the scoring method, A_UG_CAI(r) scores were compared with a simpler system to evaluate the initiation context based solely on the nucleotide at the −3 position, where U or C are considered bad context and A or G are considered better context [20] (Figure 8A, dot plot). Red dots represented by transcripts with a sub-optimal −3 context (U or C) are skewed on the y-axis towards low A_UG_CAI(r) scores, but are distributed across a broad range between 0.2 and 0.6. When the context of the second out-of-frame AUG was evaluated (x-axis), the transcripts were also skewed towards low A_UG_CAI(r) scores but with an even broader range between 0.1 and 0.8. The results suggest that an index based solely on nucleotides at −3 position is an inadequate predictor of the efficacy of translation initiation. The revised index is an improvement because it distinguishes initiation contexts with the same −3 nucleotides. The results also show that the A_UG_CAI(r) scores for the second out-of-frame AUG are not necessarily higher than the scores for the first AUG.

The A_UG_CAI(r) for *SPT10* initiation codon is 0.32. For *EBS1,* which is not targeted by leaky scanning [40], the A_UG_CAI(r) for the initiation codon is 0.44. We therefore anticipated that transcripts with scores ≥0.44 might be poor candidates for leaky scanning and those with scores ≤0.32 or lower might be good candidates. To further test whether A_UG_CAI(r) is a predictor of leaky scanning, *RDR1:* A_UG_CAI(r) = 0.50 and *ASF2:* A_UG_CAI(r) = 0.17. mRNAs were analyzed using a genetic approach. In each transcript, sequences in between the first and second AUG codons were altered to create a new in-frame AUG codon in a good context: A_UG_CAI(r) = 0.70 for *rdr1-AUG* and 0.83 for *asf2-AUG*. The mutant alleles were analyzed by comparing transcript abundance and half-lives in Nmd^+^ and Nmd^−^ strains (Figure 8B and 8C). If leaky scanning targets *RDR1* or *ASF2* mRNA for NMD, the new AUG codon should abolish the effects of NMD on RNA abundance and half-life.

The results show that leaky scanning is not a likely targeting mechanism for *RDR1* mRNA. When Nmd^+^ and Nmd^−^ strains were compared, the change in abundance was 1.8 ± 0.2 (*n* = 3, *p* = 0.001) for *RDR1* mRNA and 1.7 ± 0.4 (*n* = 3, *p* = 0.014) for *rdr1-AUG.* Using a *p* = 0.05 cutoff, the results show that mRNA abundance is not affected by the introduction of the new AUG codon. In a representative experiment (Figure 8B), the *RDR1* half-life was 3.9 ± 1.8 min in the Nmd^+^ strain and 12.6 ± 1.0 min in the Nmd^−^ strain. The FCR for half-life was 3.6 ± 1.6 (*n* = 3, *p* = 0.02), confirming prior results (Table 1) showing that *RDR1* mRNA is a direct target of NMD. The half-life of *rdr1-AUG* mRNA was 4.7 ± 0.8 min in the Nmd^+^ strain and 12.1 ± 3.6 min in the Nmd^−^ strain. The FCR for the *rdr1-AUG* half-life was 2.6 ± 0.4 (*n* = 3, *p* = 0.001). These results indicate that the introduction of a new AUG in a good context had no effect on mRNA half-life. The A_UG_CAI(r) score of 0.50 for the *RDR1* mRNA start codon is not low enough to trigger leaky scanning.

By contrast, leaky scanning is a likely targeting mechanism for *ASF2* (A_UG_CAI(r) = 0.17). When Nmd^+^ and Nmd^−^ strains were compared, the change in abundance was 1.8 ± 0.3-fold (*n* = 3, *p* = 0.003) for *ASF2* mRNA and 1.1 ± 0.2-fold (*n* = 3, *p* = 0.2) for *asf2-AUG* mRNA. Using a *p* = 0.05 cutoff, the results suggested that the introduction of the new AUG codon abolished the effect of NMD on *ASF2* abundance. In a representative experiment (Figure 8C), the *ASF2* half-life was 4.4 ± 0.3 min in the Nmd^+^ strain and 11.6 ± 4.8 min in the Nmd^−^ strain. The FCR for the half-life was 2.6 ± 0.9 (*n* = 3, *p* = 0.03). The half-life of *asf2-AUG* mRNA was 7.2 ± 1.7 min in the Nmd^+^ strain and 7.7 ± 2.7 min in the Nmd^−^ strain. The FCR for the half-life was 1.1 ± 0.03 (*n* = 3, *p* = 0.3). Since the abundance and the half-lives were statistically indistinguishable using a *p* = 0.05 cutoff, the results show that the sensitivity of *ASF2* mRNA to NMD was abolished in the *asf2-AUG* mutant. The results suggest that NMD-mediated degradation of *ASF2* mRNA is triggered by leaky scanning. Inefficient translation initiation at the *ASF2* mRNA start codon is reflected by the relatively low A_UG_CAI(r) score of 0.17.

## Discussion

This paper describes how to estimate relative rates of RNA decay on a global scale by monitoring RNA abundance on DNA arrays at time intervals following a block that prevents further transcription. Furthermore, methods were developed to estimate the proportion of direct targets with altered decay rates versus indirect targets with unaltered decay rates among 607 SAM-selected RNA targets of NMD that rose in abundance when the NMD pathway was inactivated. To estimate RNA decay rates on a global scale, alternative kinetic models describing rates of decay were tested. In order to be of practical use, data fitting had to be accomplished within the constraints imposed by limits on the number of time points used to monitor the disappearance of RNA following inhibition of transcription.

Our general experience from conventional decay rate experiments is that deviations from exponential first-order decay are commonplace. It was important that decay models recognize departures from first-order caused either by features of experimental design or biological origin. The models we considered, the methods for testing them, and the criteria for selecting an appropriate non-first-order decay model are described in detail in Text S1. In addition, the statistical methods used to control for experimental error by normalization of data to stable reference RNAs are described. Normalization procedures were needed to permit useful array-to-array, time point-to-time point, and strain-to-strain comparisons of the data.

After the normalization step, a non-first-order decay model was implemented using least squares estimations of the parameters including the decay rate (*β*_1_) for the 607 SAM-selected NMD targets. Extensive searching of *β*_1_ in a proper interval was performed. Notably, none of the *β*_1_ decay rate estimates for the 607 targets hit the boundary of the interval, implying adequacy of model fitting. To our knowledge, this estimation procedure is a novel contribution. A likelihood ratio test was used to compare *β*_1_ in Nmd^+^ and Nmd^−^ strains. This standard tool in regression setting was successfully applied in our data analysis. The results indicate that 47%–48% of the protein-coding mRNAs that are targeted by NMD are direct targets. We also estimated the proportion of direct targets using a novel application of maximum likelihood. At the 95% confidence level, maximum likelihood predicts a similar frequency of direct targets estimated to be 45% ± 5% of the SAM-selected NMD-sensitive targets.

In *S. cerevisiae,* RNA accumulation levels vary over a >1,000-fold range and decay rates vary from several minutes to several hours. Accumulation levels and decay rates do not always correlate with each other [41]. Although NMD-sensitive RNAs tend to be present at low abundance [14], there was no prior basis for assuming a similar skew toward rapid decay. Because of this, time points in the global decay rate experiment were distributed over 1 h to capture a broad range of rates with sufficient data points but at the expense of accurately describing rapid decay rates for short-lived RNAs. Our high-throughput study revealed that NMD-sensitive RNAs tend to be both non-abundant and short-lived. Many RNAs differentially disappeared in the Nmd^+^ strain in the first few minutes after inhibition of transcription, making them challenging to monitor on arrays.

Some RNAs known to respond to NMD were excluded by SAM on statistical grounds and were not analyzed, including *CTF13, SPT10, ADR1,* and *PPR1* [13,14,17]. These are all known to be among the least abundant mRNAs. The transcripts excluded in our study were also excluded in other global studies [13]. Although some NMD targets were not captured in the analyses, the results of all of the published global studies identify a large common subset of transcripts as being sensitive to NMD (Figure S2). More probe sets could have been captured using less stringent statistical parameters, but at the cost of low reliability. The 607 SAM-positive probe sets were deemed to constitute a sufficiently large fraction of NMD-sensitive targets to draw general conclusions.

The results from the global approach provided a starting point for conventional studies to learn more about how RNAs are targeted for NMD and about the relationship between direct and indirect targets. 18 transcripts were strategically selected for conventional studies to complement the high-throughput analysis of RNA decay rates. Using the global approach, the initial abundance at the time of thiolutin addition, intrinsic decay rates, differential decay rates, the distribution of time points, and methods of data fitting all influence the measurement of decay rates. Although these factors frequently converged to produce FCRs that were unexpectedly <1 in the global experiment when the results from Nmd^−^ and Nmd^+^ strains were compared, conventional analyses of decay rates consistently showed this to be an anomaly. Except for false discoveries, FCRs that were <1 in the global experiment were >1 in conventional experiments, indicating that the global approach accurately predicted direct targets within statistical limits.

### Targeting of dORFs by NMD

dORFs were uncovered as targets of NMD. Two dORFs shown to be direct targets of NMD were studied in an effort to understand the targeting mechanisms. Genetic analysis of *YIL164C/YIL165C,* consisting of in-frame ORFs separated by a 9-bp gap, revealed that translation termination at the *YIL164C* stop codon fails to trigger NMD. Leaky scanning is an unlikely targeting mechanism: A_UG_CAI(r) = 0.70. However, a putative uORF in the 5′-UTR could potentially be responsible for targeting (Table 2). The dORFs *YIL168W/YIL167W* are organized like *YIL164C/YIL165C* but without a gap. We found that the *YIL168W* stop codon targets the mRNA for NMD and is presumably recognized as a premature site for termination similar to premature termination events caused by nonsense mutations.

When a special class of dORFs were analyzed that are translated through programmed frameshifting, it was found that internal stop codons fail to trigger NMD. *EST3* mRNA *(YIL009C/YIL009C-A),* which codes for a telomerase regulator [42], was shown to be an indirect target of NMD [16], indicating that the *YIL009C* stop codon is not involved in targeting. *ABP140* mRNA *(YOR239W/YOR240W),* which codes for an actin-binding protein [43], was not identified as NMD-sensitive in three different genome-wide studies of NMD [13,14]. Most TY elements contain dORFs *(TYA/TYB)* that undergo contiguous translation by low-frequency programmed frameshift events [32,33]. Our results indicate that the TY3 transcript is insensitive to NMD, indicating that the *TY3A* stop codon is not involved in targeting. Overall, the results suggest that most stop codons associated with programmed frameshifting do not trigger NMD.

Most of the NMD-sensitive probe sets related to TY elements contain LTRs, including δ (11 probe sets), σ (1 probe set), and τ (12 probe sets). These LTRs are located as direct repeats at the ends of intact TY1/TY2, TY3, and TY4 elements, respectively. When intact elements recombine within LTR sequences, solo copies of the LTRs are left behind. Solo LTRs are found at 270 different locations in the genome. The LTR-related probe sets may detect NMD-sensitive RNAs that initiate at promoters in solo LTRs and extend into neighboring DNA. These transcripts most likely serve no functional purpose and are probably degraded due to the fortuitous presence of premature stop codons that trigger NMD. The degradation of these types of transcripts illustrates a role for NMD in RNA surveillance as opposed to its role in controlling the expression levels of transcripts that code for functional proteins.

### Targeting Mechanisms

NMD-sensitive protein-coding mRNAs were further examined to reveal what features of the RNAs cause direct targeting. Targeting by uORF termination and leaky scanning were examined in detail. Overall, the results suggest that these two mechanisms account for only a portion of direct targets.

*FZF1* mRNA is targeted for NMD by a translated uORF that starts in the 5′ leader and ends in the coding ORF. In order to abolish uORF translation and targeting for NMD, it was necessary to mutate two AUG start codons. Although the FCR for half-life in the double mutant fully accounted for the effect of NMD on the abundance of the wild-type mRNA, the single and double mutant mRNAs were generally reduced in abundance in both Nmd^+^ and Nmd^−^ strains. The 5′ end of *FZF1* mRNA is not known, but the AUG codons for the uORF could be near the 5′ end, and mutations at these sites might influence rates of transcription. This could potentially explain the NMD-independent effects on abundance that were observed. The only other verified uORF demonstrated to target a transcript for NMD is found in *CPA1* [19]. The *CPA1* uORF starts and ends in the 5′-UTR. These examples show that uORFs can target transcripts for NMD whether they are self-contained in the 5′-UTR or whether they extend into the ORF. The *CPA1* uORF is conserved in related species of yeast [35], but the *FZF1* uORF varies among related species. Three species contain one or the other of the two AUG uORF start codons in *FZF1* and five lack both AUG start codons. Thus, the mRNAs subject to uORF targeting show some evolutionary conservation but with some variation even among closely related species.

Among the 220 predicted direct targets of NMD, 135 transcripts contained a uORF potentially involved in targeting. After evaluating the median length, average distribution, and known positions of transcription start sites [34,35], the presence of translatable uORFs were ruled out for 16 transcripts, typically because transcription initiates downstream of the uORF start codon, leaving 119 candidates for uORF targeting. Among these, the uORFs in 63 transcripts were self-contained in the 5′ leader and the uORFs in 56 transcripts extended into and terminated in the coding ORF. In order for these uORFs to trigger NMD, they must be translated. 45 uORFs begin with translation start codons that reside in a poor context for initiation: A_UG_CAI(r) ≤ 0.32, leaving 74 uORFs with a likelihood of being translated. Overall, it appears highly unlikely that uORFs target more than 35% of direct targets for NMD.

Prior to this study, only one transcript, *SPT10,* has been demonstrated to use leaky scanning as a targeting mechanism [20]. This occurs when ribosomes fail to initiate and scan past the first AUG. NMD is triggered when translation initiation at a second out-of-frame AUG leads to premature termination at an out-of-frame stop codon. To estimate the frequency of use of leaky scanning as a targeting mechanism, a revised method was developed to evaluate context sequences surrounding AUG initiation codons that influence the efficiency of initiation. The codon adaptation index (A_UG_CAI) [39] was modified to account for the frequency of nucleotide usage at each context position and the weighted contribution of each nucleotide relative to the overall context. Candidate transcripts were ranked according to their A_UG_CAI(r) score. The best candidates for targeting by leaky scanning were presumed to be those transcripts with low A_UG_CAI(r) scores corresponding to a poor context for translation initiation.

We demonstrated that *ASF2* mRNA (A_UG_CAI(r) = 0.17) is targeted by leaky scanning. Although *RDR1* is a direct target, the index score is 0.50 and our results shows that leaky scanning is not the mechanism responsible for targeting. The finding that leaky scanning targets a second transcript increases the confidence that leaky scanning is a general mechanism dependent on the context surrounding AUG codons. Combined with previous results, *SPT10* mRNA with a score of 0.32 is targeted by leaky scanning [20], whereas the *EBS1* [40] and *RDR1* transcripts with scores of 0.44 and 0.50, respectively, are not targeted by leaky scanning.

The best candidates identified for leaky scanning were 53 transcripts with scores of ≤0.32 (Table S12). Combined with our analysis of uORFs, we estimate that approximately 100 transcripts out of the 220 direct targets are very likely to be targeted by one of these known mechanisms. 55 transcripts were identified as being very unlikely to be targeted by either mechanism. *RDR1* mRNA is a candidate for an alternative targeting mechanism since no uORFs were found and leaky scanning is an unlikely targeting mechanism. *PPR1* mRNA might also be targeted by an unknown mechanism [21]. It is possible that features of the 3′-UTR of some transcripts might play a role in targeting as a third mechanism based on the mutant allele *unc54(r293)* in *Caenorhabditis elegans,* which is deleted for 3′ UTR sequences and produces an NMD-sensitive transcript [44]. Further studies of the candidates for novel targeting mechanisms will be required to establish alternative mechanisms.

### Functional Relationships among NMD Targets

**Figure 9:**
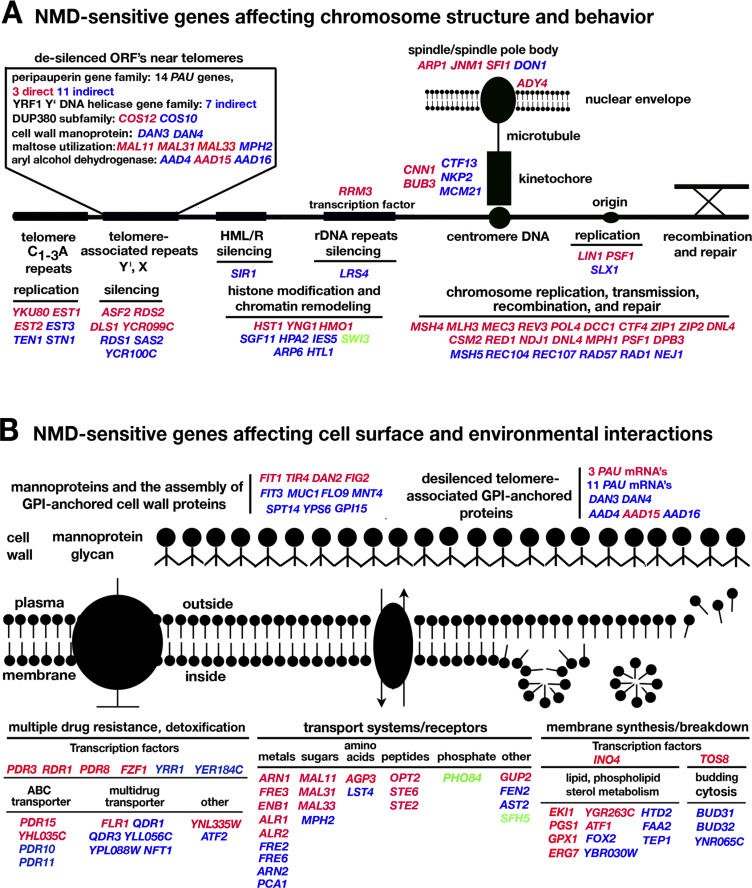
Networks of NMD-Sensitive Transcripts (A) Summary of NMD-sensitive genes coding for proteins that affect chromosome structure and behavior, including telomere replication and maintenance, chromatin silencing, replication, recombination, repair, components of the spindle apparatus such as the kinetochore and spindle pole body, and chromosome transmission. Among these, 32 probe sets detected RNAs expressed at higher levels due to de-silencing of repeated genes in six multi-gene families whose members are located in the sub-telomeric repeats near chromosome ends. (B) Summary of NMD-sensitive genes coding for proteins that affect the cell surface and environmental interactions, including surface receptors for signal transduction, macromolecular transport, synthesis breakdown of the plasma membrane, cell wall mannoproteins, and the MDR system for cellular defense against toxins. Transcripts that increase in abundance in Nmd^−^ strains are indicated in red for direct targets with altered decay rates and blue for indirect targets with unaltered decay rates. Targets that decrease in abundance in Nmd^−^ strains are indicated in green.

Striking new functional relationships are evident in the genomic data. 35% of the protein-coding NMD targets identified in this study can be organized around two central themes (Figure 9). 62 NMD-sensitive transcripts code for proteins involved in replication and maintenance of telomeres, chromatin-mediated silencing, and post-replication events related to the transmission of chromosomes during the cell division cycle: including recombination, synapsis, cohesion, disjunction, segregation of chromatids, and the formation and function of the spindle apparatus (Figure 9A). 36 transcripts are direct targets of NMD-sensitive and 26 are indirect targets. 16 NMD-sensitive transcripts were identified that code for proteins known to function in the replication and maintenance of telomeres or as chromatin remodeling factors that affect either global or local aspects of chromatin structure. Previously unrecognized NMD-sensitive transcripts affecting telomere replication and DNA silencing were uncovered, including *YKU80* mRNA coding for a subunit of the Ku complex that plays a role in telomere length maintenance [45], *SIR1* mRNA coding for a protein required for silencing of mating type cassettes [46], and *LRS4* mRNA coding for a protein required for rDNA silencing [47].

Evidence for NMD-mediated effects on telomere replication and silencing of telomere-proximal reporters was previously reported [16]. In this study, we identified 32 probe sets that detected increased levels of the RNA products of repeated genes located in the sub-telomeric repeats near chromosome ends. The probe sets, which corresponded to genes at one or both ends of 15 out of the 16 haploid chromosomes, include representatives of the *PAU* [48], *DUP380* [49], *DAN* [50], *MAL* [51], *AAD* [52,53], and *YRF* [54] multi-gene families. Most of these transcripts were predicted to be indirect targets of NMD (the exceptions could be false discoveries). As expected for genes located in silent chromatin, the RNAs were barely detectable in Nmd^+^ strains. Clusters of telomere-proximal genes residing next to each other that were co-expressed in Nmd^−^ strains included *DAN3, MAL31,* and *MAL33* on C2R, *PAU11* and *COS12(DUP380)* on C7L, *PAU18* and *YRF1* on C12L, *PAU6* and *COS10* on C14R, and *PAU20* and *AAD15* on C15L. We propose that regional de-silencing is the underlying mechanism for the expression of telomere-proximal genes in Nmd^−^ strains.

Interestingly, many of the telomere-proximal repeated genes are naturally de-silenced under anaerobic growth conditions. In Nmd^−^ strains, unscheduled de-silencing occurs under conditions of aerobic growth. The *YRF* genes differ from other telomere-proximal repeated gene families in that expression occurs when the telomere replication machinery is compromised, as is the case in Nmd^−^ strains [16]. It was suggested that the putative DNA helicase encoded by the *YRF* genes may function in protecting the integrity of chromosome ends [54]. These findings build on the previous results showing that disruption of NMD reduces the number of hexameric DNA repeats at telomeres and de-silences reporters inserted near telomeres [16]. RNAs that increase in abundance due to de-silencing constitute a previously unrecognized class of indirect targets of NMD.

The dynamics of the plasma membrane and cell wall is another central theme evident among the targets of NMD. 71 NMD-sensitive transcripts (including some of the sub-telomeric de-silenced transcripts mentioned above) code for proteins that have an impact on communication with the extracellular environment, including synthesis and breakdown of the plasma membrane, plasma membrane receptors involved in signal transduction, transport of macromolecules and nutrients, cell wall proteins, and cellular defense (Figure 9B). Of these, 32 are direct targets. 13 NMD-sensitive transcripts, including one transcription factor, *INO4,* code for proteins involved in fatty acid and phospholipid metabolism. Two transcripts required for mating, *STE6* coding for the **a**-factor membrane transporter [55] and *STE2* coding for the α-factor membrane receptor [56], are direct targets of NMD. 21 transcripts code for proteins that transport nutrients and small molecules across the plasma membrane.

Transcripts coding for cell wall mannoproteins, the enzymes for assembling GPI anchors (glycosylphosphatidylinositol) that attach mannoproteins to the cell wall, and cell wall regulators are targets of NMD [57]. The genes for these proteins are unusual because they contain Ser-Thr repeats that expand and contract in their copy number leading to proteins of differing size and antigenicity [58]. Also, many of the NMD-sensitive genes in telomere-associated silent regions code for GPI-anchored cell wall mannoproteins.

A surprising number of NMD targets play central roles in multi-drug resistance (MDR), a phenomenon observed in organisms ranging from yeast to humans, which involves complex mechanisms of intracellular reorganization to remove or sequester toxins and other agents that threaten cell viability. MDR, which is clinically relevant and is a major cause of the failure of chemotherapy in cancer patients [59], is controlled by a complex network of transcription factors that auto-regulate their own synthesis, regulate each other, and regulate the expression of ATP-binding cassette transporters, permeases, and genes involved in lipid metabolism. NMD targets key transcription factors that control MDR, especially *PDR3* [60], *PDR8* [61], *RDR1* [62], and *YRR1* [63]. The transcription factor *FZF1,* which is sensitive to NMD due to uORF targeting, is part of the MDR network that causes a cellular response to the presence of nitric oxide leading to detoxification. *FZF1* transcription, which decreases in strains carrying a mutation in *PDR3* [64], controls the expression of the five genes including the NMD-sensitive transcript *YNL335W* [65].

The levels of the transcripts coding for the Upf proteins do not appear to change during the environmental stress response in which yeast cells adapt to the sudden exposure to suboptimal environments through global changes in the gene expression program [66]. However, like the transcripts affected by NMD, many of the transcripts affected by the environmental stress response code for proteins that influence chromosome dynamics and cell surface dynamics. To explain why so many NMD-sensitive mRNAs code for proteins that influence both chromosome and cell surface properties, we suggest a possible connection between NMD and the environmental stress response that might involve post-transcriptional regulation of the Upf proteins. Further studies will be required to substantiate whether this model has merit.

### Extension of Global Methods to Other RNA Decay Pathways and Other Organisms

Using our methods it should be immediately possible to examine other RNA decay pathways on a global basis in yeast using appropriate mutants. Furthermore, recent evidence from steady-state expression profiling of human cell lines indicates that numerous RNAs are up-regulated when cells are depleted of hUPF1, including transcripts that contain 5′ uORFs, alternatively spliced nonsense RNAs, transposons, and retroviruses [67]. Using our methods for global analysis of decay rates, these findings could be extended to distinguish direct versus indirect targets. In theory, global RNA decay rates could be monitored in any multi-cellular eukaryote where cell lines are available and could be employed to examine a multiplicity of mRNA decay pathways, including but not limited to NMD. We anticipate that global decay rate experiments will provide a wealth of new information on RNA surveillance and post-transcriptional control mechanisms.

## Materials and Methods

### Strains and genes.

The strains used in this study are listed in Table S1. The sources of RNA for global gene expression studies were the congenic Saccharomyces cerevisiae strains S288C and YRZ1. S288C has a functional NMD pathway and is referred to throughout as the wild-type strain. Strain YRZ1, which is defective for NMD because it carries the null allele *upf1Δ::KanMX4,* is referred to throughout as the *upf1^−^* strain. It was shown previously that null alleles of *UPF1, UPF2, UPF3,* or all three genes affect abundance of the same subset of transcripts [14].

### Bioinformatics.

The 96 raw image files (.CEL) were processed using the method of robust multichip average [24] to remove non-biological variation between arrays resulting in background adjusted, normalized signals summarized as ln(PM) (perfect match) values for each transcript. Several alternative mathematical models describing the kinetics of RNA decay were evaluated using both simulated and actual array data to determine the model giving the best fit. The models, methods for testing the models, and other bioinformatic methods used to analyze array data are described in detail in Text S1. Genomic DNA sequences were retrieved from the *Saccharomyces* Genome Database (http://www.yeastgenome.org). Transcription factors and target genes are described in the Promoter Database of S. cerevisiae (http://rulai.cshl.edu/SCPD). The yeast transcriptome data [68] were downloaded from http://web.wi.mit.edu/young/pub/data/orf_transcriptome.txt. The RNA sequence alignment to generate the AUG initiation context was done by using the logo generation form at http://weblogo.berkeley.edu/logo.cgi. The database for transcriptional start sites in S. cerevisiae is available at http://data.cgt.duke.edu/5sage.php.

### Verification of maximum likelihood estimation.

Bootstrapping was used to validate the maximum likelihood method used to estimate the proportion of direct targets (Figure S1). 607 data points were randomly sampled from the 607 *p*-values with replacement, and *η* was estimated using the same fˆ^(1)^(*p*) and maximum likelihood method as described in the Results. The process of re-sampling and estimation was repeated 1,000 times to get a distribution of ηˆ. The distribution was very close to a normal distribution, with the mean at 0.449521, 2.5% percentile at 0.39, and 97.5% percentile at 0.508. These numbers are very close to the inference of *η* obtained using the theoretical derivation. The empirical fˆ^(1)^(*p*) using linear interpolation of 14 known direct targets (Table 1) was not strictly isotonic in the interval [0, 1]. Isotonic regression was used to obtain a revised empirical distribution denoted as ĝ^(1)^(*p*). Using this modified empirical distribution ĝ^(1)^(*p*) to replace fˆ^(1)^(*p*), the bootstrap procedure was repeated and similar results were obtained with the distribution of the mean at 0.46298, 2.5% percentile at 0.407, and 97.5% percentile at 0.522. This indicates that the inference of *η* is robust.

### RNA methods.

To prepare probes for array hybridization, cell cultures of S288C and YRZ1 were grown to mid-log phase (O.D. = 0.6) and harvested at 0, 2, 4, 6, 8, 10, 12, 15, 20, 25, 30, 35, 40, 45, 50, and 60 min after the addition of 10 μg/ml thiolutin (a gift from Pfizer, Inc., part number CP-4092), which inhibits transcription [69]. The effective concentration of thiolutin was established by assaying for growth inhibition and inhibition of transcription of selected transcripts. At each time point total RNA was isolated by hot phenol extraction [7]. Double-stranded cDNA was synthesized by two-step reverse transcription from 15 μg total RNA and 100 pmol oligo(dT) primer, purified by PLG-P/C extraction, precipitated with ethanol, and resuspended in 12 μl of RNase-free water. 5 μl of the reaction mixture was used to prepare biotinylated cRNA using the Enzo BioArray HighYield RNA transcript labeling kit (T7). Half of the in vitro transcription products were purified using Qiagen (http://www1.qiagen.com) RNeasy spin columns. The cRNA quantity was measured and adjusted as directed by the Affymetrix Technical Manual. 20 μg of biotinylated cRNA was fragmented using 6 μl of 5× fragmentation buffer in a 30-μl reaction and incubated at 94 °C for 35 min, mixed in a hybridization cocktail as described in the Affymetrix Technical Manual, and hybridized to a YG-S98 GeneChip array for 20 h. Following hybridization, the arrays were washed and stained using a GeneChip Fluidics Station 400 and scanned with a GeneArray scanner following the manufacturer's instructions. Three independent trials were performed using RNA from each of the two strains for 16 time points giving a total of 96 arrays.

To measure mRNA half-lives, transcription was inhibited by adding thiolutin dissolved in DMSO (10 μg/ml for S288c and related strains; 25 μg/ml for W303 and related strains) or by shifting cell cultures to the restrictive temperature of 39 °C for strains carrying the *rpb1–1* allele coding for a temperature-sensitive subunit of RNAP II [70]. Total RNA was extracted at time intervals following inhibition of transcription, and 20 μg of RNA from each sample was fractionated on agarose gels, transferred to GeneScreen Plus (Dupont, NEN Research Products, http://www.dupont.com) and analyzed by Northern hybridization using sequence-specific probes prepared by in vitro reverse transcription. TY3 transcripts were detected on Northern blots using a fragment complementary to nucleotides 1366–1624 in the TY3B ORF [71], which specifically recognizes transcripts from two full-length TY3 elements in the genome but does not hybridize to genomic copies of TY1, TY2, TY4, or TY5. Hybridization signals were normalized to *SCR1* RNA [72]. Graphs were generated with SigmaPlot (version 9.0). Data fitting for experiments based on conventional Northern blotting was accomplished by non-linear regression using a biphasic decay model *(y* = *ae* ^−*bx*^ + *ce* ^−*dx*^ *).*

To determine the half-lives of *YIL168W* and *ASF2,* RNA levels were measured by quantitative real-time PCR using an ABI 7900HT RT-PCR thermocycler (Applied Biosystems, http://www.appliedbiosystems.com). The cycling conditions were: 48 °C for 30 min, 90 °C for 10 min, and 40 cycles at 95 °C for 15 s and 60 °C for 1 min. RNA was treated with DNase I (Ambion, http://ambion.com) at 37 °C for 30 min and then amplified with a TaqMan one-step RT-PCR master mix (Applied Biosystems) in the presence of 0.7 μM of each gene-specific primer and 0.35 μM 5,- 6-carboxyfluorescein (6-FAM), 3,- 6-carboxytetramethylrhodaminutese (TAMRA)-labeled probe. The RNA quantity in each time-course reaction was determined by comparison with standard curves generated by amplification of the time-zero RNA sample. Data were analyzed using the SDS 2.2.1 software (Applied Biosystems).

### Mutagenesis.

Mutations were created by PCR mutagenesis. Centromeric *(CEN)* plasmid pQG29 contains *YIL164C/YIL165C* plus 800 bp and 750 bp of upstream and downstream sequence, respectively. The TAG stop codon of *YIL164C* was changed to TCG (pQG44C) or TGG (pQG44G). *CEN* plasmid pQG32 contains *YIL168W/YIL167W* plus 580 bp and 400 bp of upstream and downstream sequence, respectively. The TGA stop codon of *YIL168W* was changed to GGA (pQG47G) or AGA (pQG47A). *CEN* plasmid pQG20 contains *FZF1* plus 195 bp and 731 bp of upstream and downstream sequence, respectively. Two uORF AUG codons starting at −64 and −58 were changed to AGG codons to generate *fzf1-Δ1* (AGG at −64) (pQG38), *fzf1-Δ2* (AGG at −58) (pQG39), and the double mutant *fzf1-Δ1,-Δ2* (pQG40). *CEN* plasmid pZW29 contains *RDR1* plus 317 bp and 595 bp of upstream and downstream sequence, respectively. *CEN* plasmid pZW28 contains *ASF2* plus 344 bp and 516 bp of upstream and downstream sequence, respectively.

To create an in-frame AUG in the *RDR1* ORF, GGCTTGC was changed to AATGTCT between the first AUG and the downstream out-of-frame AUG (pZW31) (Figure 8B). To create an in-frame AUG in the *ASF2* ORF, AATCGT was changed to ATGTCT between the first AUG and the downstream out-of-frame AUG (pZW30) (Figure 8C). The wild-type genes were separately deleted in strains W303 *(UPF1)* and AAy320 *(upf1Δ)* and replaced with *KanMX4,* conferring resistance to 200 μg/ml geneticin [73], to generate strains QGy29, QGy30, QGy33, QGy34, QGy11, QGy12, ZWY58, and ZWY59 (Table S1). These strains were transformed [74] with the appropriate plasmids.

### Revised codon adaptation index.

In order to determine whether transcripts were eligible for targeting by leaky scanning, the context surrounding the initiation codon was evaluated as follows: A revised AUG context adaptation index termed A_UG_CAI(r) was created based on a previous methodology developed by Miyasaka [39]. Two modifications were made. To avoid the arbitrary selection of reference genes, which should be highly representative of the nucleotide/codon usage bias, 63 highly expressed genes were used in the A_UG_CAI(r) calculation. They represent 1% of the yeast transcriptome and were selected from iterations of computational algorithms used to detect the most predominant codon bias [75].

The relative weight given to each nucleotide in the AUG context was calculated as follows. Position-specific weight matrices (PWM) were generated by calculating the frequency of a nucleotide *j* (A, U, G, C) at position *i* (−6, −5, −4, −3, −2, −1, 4, 5, 6) of the AUG initiation context from 63 reference genes. Since the occurrence of some nucleotides at some positions was zero, a value of 1 was added to the number of occurrences of each nucleotide at each position. The information content (IC) (bit score, see Figure 8), which measures the sequence conservation at each position, was calculated using the equation in [76]:

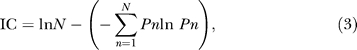
where *N* = 4 (A, U, G, C) and *Pn* is the frequency of a nucleotide at a particular sequence position as calculated in the PWM. The corrected weight for each nucleotide was calculated by multiplying the frequency for nucleotide *j* at position *i* (the values in PWM) by the IC value at position *I* (Table S11). Unlike the values in [39], the weight for the optimum nucleotide at each position is not equal to 1, but is dependent on both the nucleotide frequency and the weighted importance of the position. When the context adaptation index is calculated using the equation from [39], the maximum value is 0.22.

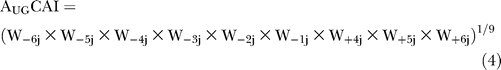



In order to obtain values ranging from 0 to 1, the A_UG_CAI(r) was calculated using a denominator equal to the maximum A_UG_CAI value of 0.22. A unique A_UG_CAI(r) for each individual mRNA was calculated using equation:

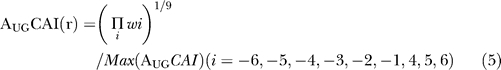



The higher the A_UG_CAI(r) value, the closer the context is to the optimal context for translation initiation. Low values correspond to a higher probability of read-through without initiation.

Candidates for targeting by leaky scanning were screened as follows: To avoid the presence of introns while at the same time retaining 5′ and 3′ flanking sequences for each gene, source code was written to attach the intronless coding sequence and flanking sequences downloaded separately from the SGD (Saccharomyces Genome Database). Source code was written to scan each ORF from start codon to termination codon. The algorithm calculates the A_UG_CAI(r) value of the first AUG and identifies those ORFs where the second AUG is out-of-frame and followed a termination codon in the same alternate frame.

## Supporting Information

Figure S1 Bootstrap Test of Maximum Likelihood Estimate for the Proportion of Direct Targets (47 MB TIF)

Figure S2 Comparison of NMD Targets Identified in Different Published Studies (2.2 MB TIF)

Table S1 Strains and Plasmids (36 KB DOC)

Table S2 Positive NMD-Sensitive Probe Sets Selected by SAM (601 KB DOC)

Table S3 Negative NMD-Sensitive Probe Sets Selected by SAM (35 KB DOC)

Table S4 Stable Transcripts Used to Generate a Reference Line (52 KB DOC)

Table S5 Performance Assessments of Decay Model Using Simulated Data (30 KB DOC)

Table S6 Performance Assessments of Decay Model Using Actual Data (23 KB DOC)

Table S7 Supporting Information on mRNA Half-Life Experiment (43 KB DOC)

Table S8 Accession Numbers for Genes/Proteins (32 KB DOC)

Table S9 Analysis of the False Discovery Rate (23 KB DOC)

Table S10 uORFs in Direct Targets of NMD (155 KB DOC)

Table S11 Nucleotide Weight Matrices Used to Calculate A_UG_CAI(r) (49 KB DOC)

Table S12 Leaky Scanning Analysis (264 KB DOC)

Text S1 Supplementary Methods (542 KB DOC)

### Accession Numbers

Source codes for all of the algorithms developed for use in this study were deposited at http://www.SourceForge.net. The Entrez Gene (http://www.ncbi.nlm.nih.gov/entrez/query.fcgi?db=gene) accession numbers for some of the entities discussed in this paper are *ADR1* (851802), *ARG81* (854874), *ASF2* (851330), *COS10* (855812), *COS12* (852628), *CPA1* (854479), *CTF13* (855119), *DAN3* (852603), *EBS1* (851787), *FZF1* (852638), *HHF2* (855701), hUPF1 (5976), *INO4* (854042), *LRS4* (852049), *MAF1* (851568), *MAL31* (852601), *MAL33* (852600), *MED1* (856183), *PAU6* (855813), *PAU11* (852630), *PAU18* (850662), *PDR3* (852278), *PDR8* (850971), *PET18* (850382), *PPR1* (850701), *RDR1* (854562), *RPB1* (851415), *RRN10* (852256), *RRP6* (854162), *SAS2* (855157), *SET7* (851844), *SGF11* (856060), *SK17* (854243), *S1R1* (853976), *SPT10* (853315), *STE2* (850518), *STE6* (853671), *SUP35* (851752), *SUP45* (852440), *UGA3* (851384), *unc-54* (259839), Upflp (855104), Upf2p (856476), Upf3p (852963), *URA3* (856692), *XRN1* (852702), *YER039C-A/YER039C* (856762/856761), *YIL009C/YIL009C-A* (EST3) (854806), *YIL164C/YIL165C* (854642/8854641), *YIL168W/YIL167W* (854638/854639), *YKU80* (855132), *YNL335W* (855381), *YOL163W/YOL162W* (854001/854002), *YOR239W/YOR240W* (ABP140) (854414), *YRF1–4* (851187), and *YRR1* (854333).

## Abbreviations

CAI: codon adaptation index
dORF: disabled open reading frame
FCR: fold change ratio
FDR: false discovery rate
LTR: long terminal repeat
MDR: multi-drug resistance
NMD: nonsense-mediated mRNA decay
ORF: open reading frame
pdf: probability density function
SAM: significance analysis of microarrays
TY: yeast transposon
uORF: upstream open reading frame
