# Supplementary figures and images for: Impact of Nonsense-Mediated mRNA Decay on the Global Expression Profile of Budding Yeast
Source: PLoS Genet. 2006 Nov 24;2(11):e203. doi: 10.1371/journal.pgen.0020203 (PMC1657058; doi:10.1371/journal.pgen.0020203)

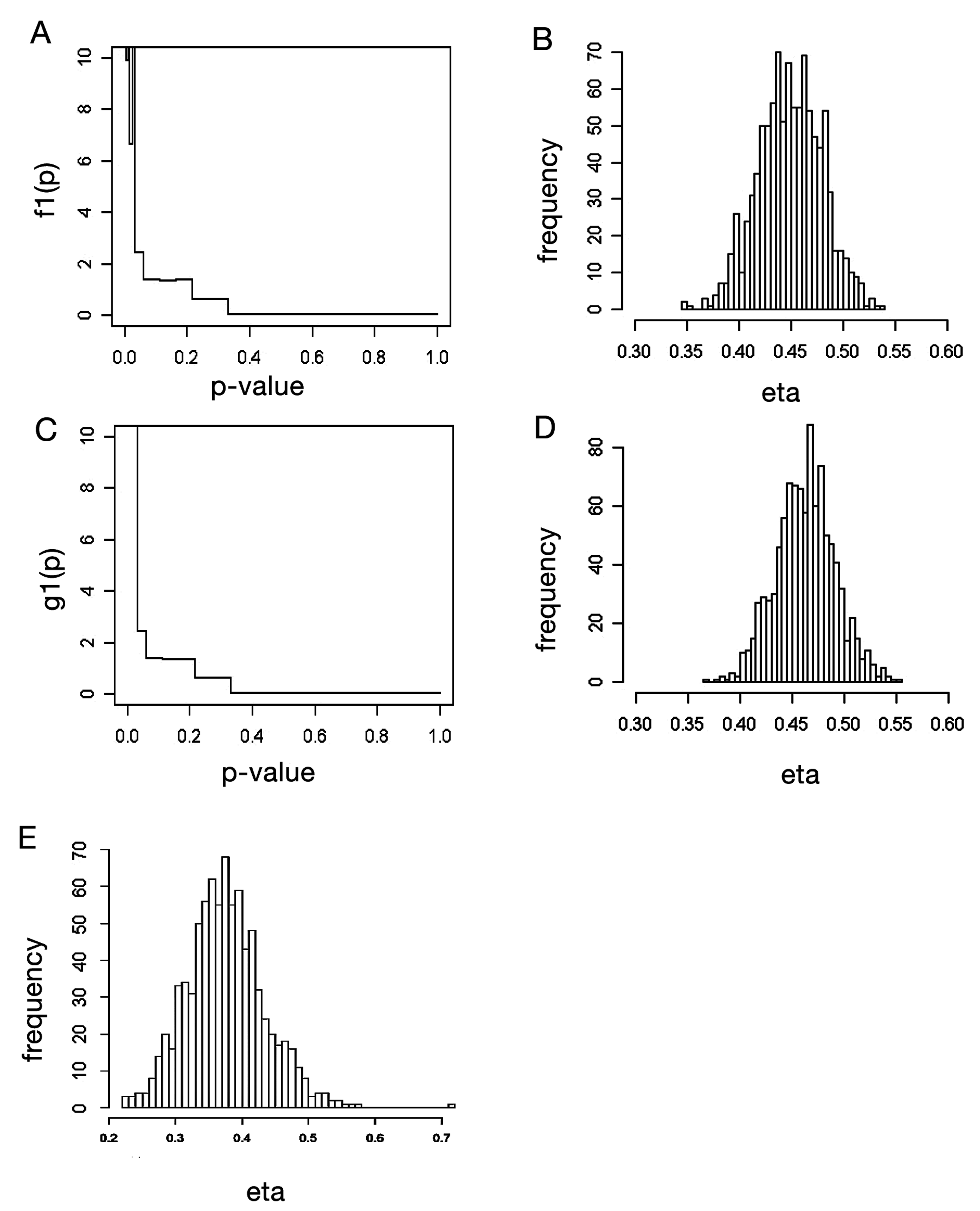

Supplement: Figure S1 — (47 MB TIF) [file pgen.0020203.sg001.tif]

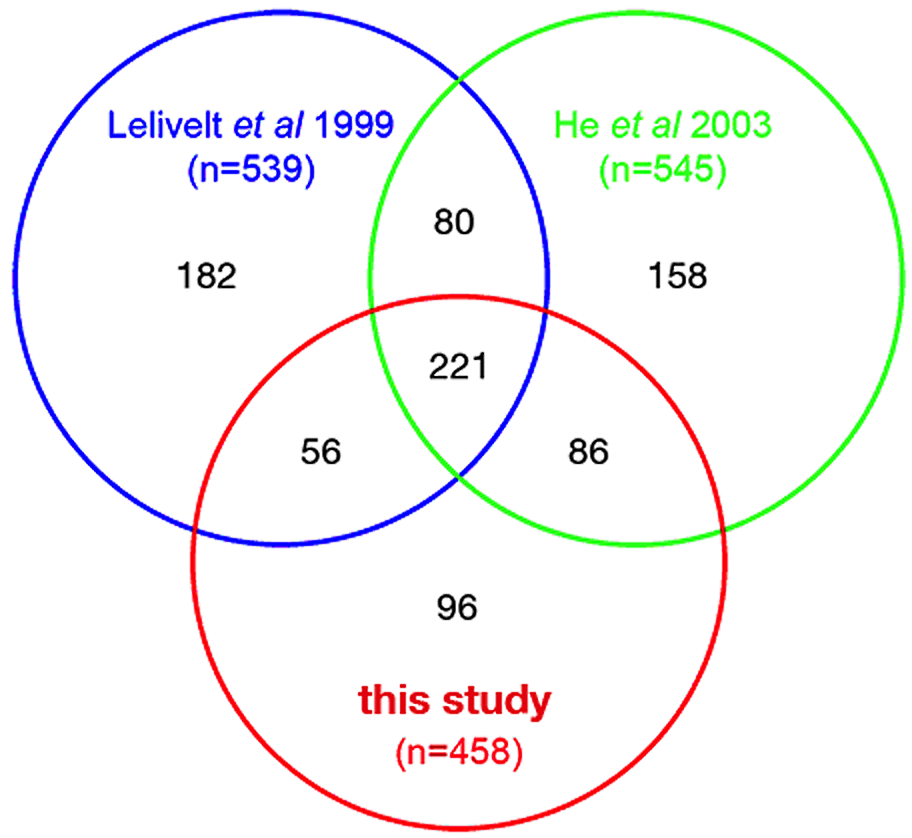

Supplement: Figure S2 — (2.2 MB TIF) [file pgen.0020203.sg002.tif]
