# Supplementary material for: Impact of Nonsense-Mediated mRNA Decay on the Global Expression Profile of Budding Yeast
Source: PLoS Genet. 2006 Nov 24;2(11):e203. doi: 10.1371/journal.pgen.0020203 (PMC1657058; doi:10.1371/journal.pgen.0020203)
Supplement: Table S1 — (36 KB DOC) [file pgen.0020203.st001.doc]

|  | **Table S1.** Yeast strains and plasmids |
| --- | --- |
| Strain/plasmid | Genotype/description |
|  |  |
| YRZ1 | *MATα SUC2* *mal mel gal2 CUP1 flo1 flo8-1 upf1ΔKanMX4* |
| S288C  AAY334^a^ | *MATα SUC2* *mal mel gal2 CUP1 flo1 flo8-1*  *MAT****a*** *ura3-1 his3-11,15 trp1-1 leu2-3,112 rpb1-1* |
| AAY335^a^ | *MAT****a*** *ura3-1 his3-11,15 trp1-1 leu2-3,112 rpb1-1 upf1-Δ2::URA3* |
| AAY320^a^ | *MAT****a*** *ura3-1 his3-11,15 leu2-3,112 trp1-1 ade2-1 can1-100 upf1-∆2::URA3* |
| QGY11 | *MAT****a*** *fzf1::KanMX4 ura3-1 his3-11 leu2-3,112 trp1-1 ade2-1 can1-100 GAL2* |
| QGY12 | *MAT****a*** *upf1∆ fzf1Δ::KanMX4 ura3-1 his3-11,15 leu2-3,112 trp1-1 ade2-1 can1-100* |
| QGY13 | *MAT****a*** *fzf1::KanMX4 ura3-1 or 3-15 his3-11,15 leu2-3,112 trp1-1 rpb1-1* |
| QGY14 | *MAT****a*** *upf1∆ fzf1::KanMX4 ura3-1 or 3-15 his3-11,15 leu2-3,112 trp1-1 rpb1-1* |
| QGY29 | *MAT****a*** *yil164c&165c::KanMX4 ura3-1 his3-11 leu2-3,112 trp1-1 ade2-1 can1-100 GAL2* |
| QGY30 | *MAT****a*** *upf1∆ yil164c&165c::KanMX4 ura3-1 his3-11,15 leu2-3,112 trp1-1 ade2-1 can1-100* |
| QGY33 | *MAT****a*** *yil168w&167w::KanMX4 ura3-1 his3-11 leu2-3,112 trp1-1 ade2-1 can1-100 GAL2* |
| QGY34 | *MAT****a*** *upf1∆ yil168w&167w::KanMX4 ura3-1 his3-11,15 leu2-3,112 trp1-1 ade2-1 can1-100* |
| QGY42 | *MAT****a*** *ura3-1 his3-11,15 leu2-3,112 trp1-1 ade2-1 can1-100 upf1-∆ xrn1∆::KanMX4* |
| QGY46 | *MAT****a*** *ura3-1 his3-11,15 leu2-3,112 trp1-1 ade2-1 can1-100 upf1-∆2 ski7∆::KanMX4* |
| QGY48 | *MAT****a*** *ura3-1 his3-11,15 leu2-3,112 trp1-1 ade2-1 can1-100 upf1-∆2 rrp6::KanMX4* |
| W303a | *MAT****a*** *ura3-1 his3-11 leu2-3,112 trp1-1 ade2-1 can1-100 GAL2* |
| ZWY56 | *MATα leu2∆ his3∆ ura3∆ lys2∆ asf2::KanMX4* |
| ZWY57 | *MATα leu2∆ his3∆ ura3∆ lys2∆ rdr1::KanMX4* |
| ZWY58 | MATα upf1∆ leu2∆ his3∆ ura3∆ lys2∆ asf2::KanMX4 |
| ZWY59 | *MATα upf1∆ leu2∆ his3∆ ura3∆ lys2∆ rdr1::KanMX4* |
| pRS313^b^ | *CEN4 HIS3 ARS4* |
| pRS314^b^ | *CEN4 TRP1* ARS4 |
| pQG20 | pRS313 *FZF1* (genomic clone) |
| pQG21 | pRS314 *FZF1* (genomic clone) |
| pQG29 | pRS313 *YIL164C/YIL165C* (genomic clone) |
| pQG30 | pRS314 *YIL164C/YIL165C* (genomic clone) |
| PQG32 | pRS313 *YIL168W/YIL167W* (genomic clone) |
| pQG33 | pRS314 *YIL168W/YIL167W* (genomic clone) |
| pQG38 | pRS313 *fzf1-Δ1* |
| pQG39 | pRS313 *fzf1-Δ2* |
| pQG40 | pRS313 *fzf1-Δ1,-Δ2* |
| pQG44C | pRS313 *yil164c-UCG* |
| pQG44G | pRS313 *yil164C-UGG* |
| pQG47A | pRS313 *yil168W-AGA* |
| pQG47G | pRS313 *yil168W-GGA* |
| pZW28 | pRS313 *ASF2* (genomic clone) |
| pZW29 | pRS313 *RDR1* (genomic clone) |
| pZW30 | pRS313 *asf2-AUG* |
| pZW31 pEGTy3-1^c^ | pRS313 *rdr1-AUG*  BglII-KpnI fragment from TY3B (nt 1366-1624) |
|  |  |

^a^Kebaara B, Nazarenus T, Taylor R, Forch A, Atkin AL (2003) The Upf-dependent decay of wild-type *PPR1* mRNA depends on its 5'-UTR and first 92 ORF nucleotides. Nucl Acids Res 31: 3157-3165.

^b^Sikorski RS, Hieter P (1989) A system fof shuttle vectors and yeast host strains designed for efficient manipulation of DNA in *Saccharomyces cerevisiae*. Genetics 122: 19-27.

^c^provided by S. Sandmeyer.
