# Supplementary material for: Impact of Nonsense-Mediated mRNA Decay on the Global Expression Profile of Budding Yeast
Source: PLoS Genet. 2006 Nov 24;2(11):e203. doi: 10.1371/journal.pgen.0020203 (PMC1657058; doi:10.1371/journal.pgen.0020203)
Supplement: Table S2 — (601 KB DOC) [file pgen.0020203.st002.doc]

| **Table S2**. Positive NMD-sensitive probe sets selected by SAM  Probe Set^a^ ORF Gene mut t1/2 wt t1/2 FCR -2lnλ *p*-value | | | | | | | |
| --- | --- | --- | --- | --- | --- | --- | --- |
| 6678_at | YDL186W | *---* | 4.552252 | 9.354655 | 0.48663 | 26.72527 | 2.35E-07 |
| 4568_at | YHL040C | *ARN1* | 7.253604 | 21.16056 | 0.342789 | 23.61452 | 1.18E-06 |
| 7044_at | non-annotated | *---* | 4.652302 | 9.054505 | 0.513811 | 22.72979 | 1.86E-06 |
| 7568_at | YPR170C | *---* | 4.352152 | 8.654304 | 0.502889 | 20.9348 | 4.75E-06 |
| 6052_at | YDR402C | *DIT2* | 5.452703 | 11.75586 | 0.463829 | 20.2381 | 6.84E-06 |
| 5855_at | non-annotated | *---* | 4.052002 | 10.05501 | 0.402984 | 19.48413 | 1.01E-05 |
| 4237_at | YIL165C | *NIT1* | 4.352152 | 8.954454 | 0.486032 | 18.75208 | 1.49E-05 |
| 8716_s_at | YOL165C | *AAD15* | 7.353654 | 4.652302 | 1.580648 | 17.69577 | 2.59E-05 |
| 6766_at | non-annotated | *---* | 4.752352 | 8.754354 | 0.542856 | 17.52422 | 2.84E-05 |
| 4855_at | YGR154C | *---* | 7.953954 | 24.66231 | 0.322515 | 17.51985 | 2.84E-05 |
| 8723_at | YOL158C | *ENB1* | 7.653804 | 19.05951 | 0.401574 | 17.19915 | 3.37E-05 |
| 9196_s_at | YNL335W | *---* | 5.352653 | 9.954955 | 0.537687 | 17.03232 | 3.67E-05 |
| 5065_at | YGL042C | *---* | 5.152553 | 10.65531 | 0.483567 | 15.5484 | 8.04E-05 |
| 7115_at | YBR250W | *---* | 4.552252 | 8.754354 | 0.519999 | 14.66288 | 0.000129 |
| 7553_at | YPR200C | *ARR2* | 4.252102 | 7.953954 | 0.53459 | 14.57507 | 0.000135 |
| 8208_at | YOR318C | *---* | 4.552252 | 8.054004 | 0.565216 | 14.33675 | 0.000153 |
| 4719_at | YGR289C | *MAL11* | 7.053504 | 16.45821 | 0.428571 | 14.32851 | 0.000154 |
| 6965_i_at | non-annotated | *---* | 2.951451 | 6.053003 | 0.487601 | 13.89502 | 0.000193 |
| 5284_at | non-annotated | *---* | 4.952452 | 8.354154 | 0.592813 | 13.7751 | 0.000206 |
| 5536_at | YER187W | *---* | 4.852402 | 9.954955 | 0.487436 | 13.57835 | 0.000229 |
| 10803_at | YKL209C | *STE6* | 5.352653 | 8.354154 | 0.640717 | 13.36967 | 0.000256 |
| 4383_at | YHR134W | *WSS1* | 3.451702 | 6.253103 | 0.551998 | 13.32701 | 0.000262 |
| 3986_f_at | --- | *---* | 4.652302 | 11.95596 | 0.38912 | 13.24814 | 0.000273 |
| 10857_at | YJR155W | *AAD10* | 6.053003 | 3.651802 | 1.657539 | 12.65916 | 0.000374 |
| 6677_at | YDL187C | *---* | 3.551752 | 8.154054 | 0.435581 | 12.5239 | 0.000402 |
| 5491_at | non-annotated | *---* | 4.552252 | 7.953954 | 0.572326 | 12.35892 | 0.000439 |
| 7546_at | YPR194C | *OPT2* | 5.552753 | 11.45571 | 0.484715 | 12.23587 | 0.000469 |
| 10216_at | YLR097C | *HRT3* | 4.952452 | 7.853904 | 0.630572 | 12.20613 | 0.000476 |
| 5535_at | YER186C | *---* | 4.452202 | 9.154555 | 0.486337 | 12.11944 | 0.000499 |
| 4015_at | non-annotated | *---* | 5.352653 | 8.454204 | 0.633135 | 12.09688 | 0.000505 |
| 9281_at | YMR316C-B | *---* | 4.252102 | 7.553754 | 0.562912 | 11.19438 | 0.00082 |
| 7422_at | YBL075C | *SSA3* | 6.053003 | 3.651802 | 1.657539 | 11.00542 | 0.000908 |
| 6416_at | YDR047W | *HEM12* | 4.452202 | 7.753854 | 0.574192 | 10.81933 | 0.001004 |
| 9055_at | YNL204C | *SPS18* | 4.352152 | 7.353654 | 0.591835 | 10.7417 | 0.001047 |
| 8194_at | YOR350C | *MNE1* | 5.252603 | 7.953954 | 0.660376 | 10.44785 | 0.001228 |
| 6744_at | non-annotated | *---* | 3.451702 | 6.553253 | 0.526716 | 10.23201 | 0.00138 |
| 5913_at | YDR534C | *FIT1* | 5.952953 | 10.05501 | 0.592039 | 10.20142 | 0.001403 |
| 6122_at | YDR336W | *---* | 3.751852 | 7.653804 | 0.490194 | 10.16724 | 0.00143 |
| 5283_at | non-annotated | *---* | 5.852903 | 8.754354 | 0.66857 | 10.07998 | 0.001499 |
| 7367_at | YBR008C | *FLR1* | 5.052503 | 9.154555 | 0.551911 | 9.976786 | 0.001585 |
| 7118_at | YBR253W | *SRB6* | 4.952452 | 7.953954 | 0.62264 | 9.82449 | 0.001722 |
| 5425_at | YFL055W | *AGP3* | 7.453704 | 11.85591 | 0.628691 | 9.69532 | 0.001847 |
| 5694_at | YER039C | *HVG1* | 4.552252 | 7.453704 | 0.610737 | 9.68626 | 0.001857 |
| 3876_at | YJLWTAU4 | *---* | 2.151051 | 6.953453 | 0.30935 | 9.670218 | 0.001873 |
| 5534_at | YER185W | *---* | 4.152052 | 7.753854 | 0.535482 | 9.644395 | 0.001899 |
| 7809_at | YPL034W | *---* | 3.551752 | 6.753353 | 0.525924 | 9.135197 | 0.002507 |
| 6349_at | YDR114C | *---* | 5.352653 | 8.154054 | 0.656441 | 9.134551 | 0.002508 |
| 5385_at | YFL003C | *MSH4* | 4.252102 | 7.653804 | 0.555554 | 8.952062 | 0.002772 |
| 8237_at | YOR303W | *CPA1* | 6.953453 | 4.952452 | 1.404042 | 8.934842 | 0.002798 |
| 8930_at | YNL057W | *---* | 5.652803 | 9.754855 | 0.579486 | 8.74052 | 0.003112 |
| 4631_at | non-annotated | *---* | 3.151552 | 6.353153 | 0.496061 | 8.564218 | 0.003428 |
| 9571_at | YMR085W | *---* | 7.953954 | 11.45571 | 0.694322 | 8.5127 | 0.003527 |
| 8180_at | YOR381W | *FRE3* | 5.052503 | 7.553754 | 0.668873 | 8.470312 | 0.00361 |
| 6996_at | non-annotated | *---* | 4.152052 | 7.953954 | 0.522011 | 8.450296 | 0.00365 |
| 8548_at | YOL014W | *---* | 4.352152 | 7.553754 | 0.576158 | 8.405713 | 0.00374 |
| 10289_f_at | YLR037C | *DAN2* | 5.752853 | 8.554254 | 0.672514 | 8.402186 | 0.003748 |
| 6616_at | YDL115C | *IWR1* | 4.852402 | 7.753854 | 0.625805 | 8.375541 | 0.003803 |
| 8547_at | YOL015W | *---* | 5.552753 | 8.454204 | 0.656804 | 8.344046 | 0.00387 |
| 10495_at | YKR070W | *---* | 5.652803 | 8.754354 | 0.645713 | 8.287071 | 0.003993 |
| 2929_at | --- | *---* | 5.152553 | 12.65631 | 0.407113 | 8.237024 | 0.004104 |
| 4238_at | YIL164C | *NIT1* | 5.652803 | 9.954955 | 0.567838 | 8.224507 | 0.004133 |
| 5298_at | YGL262W | *---* | 5.852903 | 9.754855 | 0.599999 | 8.096466 | 0.004435 |
| 9510_at | YMR114C | *---* | 6.253103 | 9.254605 | 0.675675 | 8.063929 | 0.004516 |
| 4142_at | YIL029C | *---* | 5.852903 | 9.554755 | 0.612564 | 8.063086 | 0.004518 |
| 5760_at | YEL030W | *ECM10* | 4.652302 | 7.153554 | 0.650348 | 7.98284 | 0.004722 |
| 4675_at | non-annotated | *---* | 4.352152 | 6.853403 | 0.635035 | 7.959982 | 0.004782 |
| 4739_at | YGR263C | *---* | 4.152052 | 6.653303 | 0.624059 | 7.929341 | 0.004864 |
| 4586_at | non-annotated | *---* | 5.052503 | 8.254104 | 0.61212 | 7.921067 | 0.004886 |
| 10758_at | YKL161C | *---* | 7.453704 | 5.252603 | 1.41905 | 7.917767 | 0.004895 |
| 4284_at | YIL167W | *SDL1* | 5.952953 | 10.35516 | 0.574878 | 7.908911 | 0.004919 |
| 4210_at | YIL100W | *---* | 6.553253 | 10.55526 | 0.620852 | 7.709837 | 0.005492 |
| 9095_at | YNL254C | *---* | 8.654304 | 16.25811 | 0.532307 | 7.678364 | 0.005589 |
| 4295_at | non-annotated | *---* | 2.851401 | 6.453203 | 0.441858 | 7.676085 | 0.005596 |
| 9236_at | non-annotated | *---* | 6.353153 | 10.35516 | 0.613526 | 7.664566 | 0.005632 |
| 9942_at | YLR363C | *NMD4* | 6.853403 | 4.552252 | 1.505497 | 7.579322 | 0.005904 |
| 10373_at | YLL057C | *JLP1* | 6.253103 | 10.25511 | 0.609755 | 7.511216 | 0.006132 |
| 4032_f_at | YIR043C | *---* | 3.351652 | 8.054004 | 0.416147 | 7.49134 | 0.0062 |
| 5308_at | YFR054C | *---* | 6.053003 | 9.254605 | 0.654053 | 7.340532 | 0.006742 |
| 6054_at | YDR403W | *DIT1* | 5.052503 | 7.453704 | 0.677851 | 7.243931 | 0.007114 |
| 4681_at | non-annotated | *---* | 4.652302 | 7.153554 | 0.650348 | 7.226423 | 0.007184 |
| 5479_r_at | non-annotated | *---* | 4.752352 | 9.154555 | 0.519124 | 7.217664 | 0.007219 |
| 5537_at | YER188W | *---* | 5.552753 | 8.554254 | 0.649122 | 7.147998 | 0.007505 |
| 5430_at | YFL050C | *ALR2* | 4.852402 | 7.753854 | 0.625805 | 7.12485 | 0.007602 |
| 4649_r_at | non-annotated | *---* | 5.252603 | 8.554254 | 0.614034 | 7.106682 | 0.00768 |
| 7333_at | YBR020W | *GAL1* | 5.752853 | 8.654304 | 0.664739 | 6.999404 | 0.008154 |
| 8639_at | YOL104C | *NDJ1* | 6.253103 | 9.154555 | 0.683059 | 6.91741 | 0.008536 |
| 8049_at | non-annotated | *---* | 5.852903 | 8.554254 | 0.68421 | 6.860445 | 0.008812 |
| 4159_at | YIL058W | *---* | 5.052503 | 7.753854 | 0.651612 | 6.807047 | 0.00908 |
| 4723_f_at | YGR294W | *PAU4* | 5.252603 | 7.353654 | 0.714285 | 6.799519 | 0.009118 |
| 7925_at | YPL189W | *GUP2* | 5.252603 | 7.553754 | 0.695363 | 6.767809 | 0.009282 |
| 7494_at | non-annotated | *---* | 6.553253 | 10.05501 | 0.65174 | 6.759779 | 0.009324 |
| 9280_at | non-annotated | *---* | 5.652803 | 8.454204 | 0.668638 | 6.67131 | 0.009798 |
| 6784_at | YCR089W | *FIG2* | 5.152553 | 7.853904 | 0.65605 | 6.668283 | 0.009814 |
| 7823_at | YPL066W | *---* | 3.651802 | 6.153053 | 0.593494 | 6.667933 | 0.009816 |
| 10190_at | YLR165C | *PUS5* | 4.752352 | 6.853403 | 0.69343 | 6.620004 | 0.010084 |
| 3624_at | YNRCDELTA9 | *---* | 3.551752 | 6.053003 | 0.586775 | 6.584318 | 0.010288 |
| 5579_at | YER139C | *---* | 8.654304 | 5.352653 | 1.616825 | 6.57209 | 0.010359 |
| 9687_at | YML023C | *---* | 3.751852 | 5.852903 | 0.641024 | 6.538671 | 0.010555 |
| 9594_at | YMR064W | *AEP1* | 5.952953 | 4.352152 | 1.367818 | 6.484115 | 0.010884 |
| 5767_at | YEL023C | *---* | 3.551752 | 6.153053 | 0.577234 | 6.474063 | 0.010946 |
| 7356_at | YBL005W | *PDR3* | 5.452703 | 7.553754 | 0.721853 | 6.413163 | 0.011328 |
| 7071_s_at | YBR298C | *MAL31* | 8.854404 | 15.95796 | 0.554858 | 6.408423 | 0.011358 |
| 6338_at | YDR147W | *EKI1* | 4.652302 | 7.053504 | 0.659573 | 6.40713 | 0.011366 |
| 9868_at | YLL018C-A | *COX19* | 8.254104 | 4.352152 | 1.896557 | 6.402127 | 0.011398 |
| 9010_at | YNL158W | *---* | 8.254104 | 5.152553 | 1.601945 | 6.361451 | 0.011663 |
| 4413_at | YHR120W | *MSH1* | 3.051502 | 5.052503 | 0.603958 | 6.29583 | 0.012102 |
| 10575_at | YKR012C | *---* | 4.652302 | 6.753353 | 0.688888 | 6.164678 | 0.013033 |
| 7185_at | YBR184W | *---* | 4.352152 | 6.553253 | 0.664121 | 6.150778 | 0.013135 |
| 4680_at | non-annotated | *---* | 5.252603 | 8.954454 | 0.586591 | 6.069455 | 0.013754 |
| 6852_at | YCR020C | *PET18* | 4.152052 | 6.653303 | 0.624059 | 6.068848 | 0.013759 |
| 5251_f_at | YGL261C | *PAU4* | 5.052503 | 7.053504 | 0.716311 | 6.065201 | 0.013787 |
| 8680_at | YOL108C | *INO4* | 3.651802 | 6.053003 | 0.603304 | 6.060327 | 0.013825 |
| 4534_at | YHR015W | *MIP6* | 4.652302 | 7.353654 | 0.632652 | 6.057652 | 0.013846 |
| 8817_at | YNR058W | *BIO3* | 4.652302 | 6.553253 | 0.709923 | 6.011545 | 0.014213 |
| 9548_at | YMR106C | *YKU80* | 4.952452 | 6.953453 | 0.712229 | 5.997894 | 0.014323 |
| 8117_i_at | non-annotated | *---* | 3.651802 | 6.253103 | 0.583998 | 5.989941 | 0.014388 |
| 6690_at | YDL218W | *---* | 5.352653 | 7.953954 | 0.672955 | 5.878858 | 0.015324 |
| 6738_at | non-annotated | *---* | 4.952452 | 7.453704 | 0.664428 | 5.849964 | 0.015577 |
| 4285_at | YIL166C | *---* | 5.452703 | 7.953954 | 0.685534 | 5.822528 | 0.015822 |
| 3448_s_at | YDRCSIGMA2 | *PPT1* | 4.252102 | 5.952953 | 0.714285 | 5.742123 | 0.016563 |
| 5831_at | non-annotated | *---* | 6.253103 | 9.554755 | 0.654449 | 5.729133 | 0.016686 |
| 7906_at | YPL164C | *MLH3* | 5.652803 | 8.254104 | 0.684848 | 5.706399 | 0.016903 |
| 8236_at | YOR302W | *---* | 9.954955 | 5.952953 | 1.672272 | 5.692214 | 0.01704 |
| 9912_at | YLR421C | *RPN13* | 4.952452 | 6.653303 | 0.74436 | 5.66449 | 0.017312 |
| 8700_at | YOL134C | *---* | 5.252603 | 7.153554 | 0.734265 | 5.546898 | 0.018513 |
| 6694_at | YDL214C | *PRR2* | 11.95596 | 36.56827 | 0.326949 | 5.530961 | 0.018683 |
| 4668_at | non-annotated | *---* | 2.851401 | 4.752352 | 0.599998 | 5.53093 | 0.018683 |
| 5300_at | YFR046C | *CNN1* | 4.952452 | 7.453704 | 0.664428 | 5.527901 | 0.018716 |
| 5811_at | YEL073C | *---* | 6.153053 | 3.751852 | 1.640004 | 5.520012 | 0.0188 |
| 11227_at | YJL199C | *---* | 6.353153 | 8.854404 | 0.717513 | 5.508299 | 0.018926 |
| 8176_at | YOR377W | *ATF1* | 4.752352 | 7.153554 | 0.664334 | 5.45776 | 0.019482 |
| 3232_f_at | YGRCTAU3 | *---* | 4.152052 | 6.553253 | 0.633586 | 5.37302 | 0.020451 |
| 8409_at | YOR162C | *YRR1* | 7.453704 | 11.65581 | 0.639484 | 5.326784 | 0.021 |
| 8537_at | YOR019W | *---* | 6.453203 | 9.854905 | 0.654821 | 5.312875 | 0.021168 |
| 9199_at | YNL331C | *AAD14* | 4.752352 | 6.353153 | 0.748031 | 5.307744 | 0.021231 |
| 4609_at | non-annotated | *---* | 4.152052 | 6.553253 | 0.633586 | 5.285641 | 0.021502 |
| 7818_at | YPL071C | *---* | 3.651802 | 5.752853 | 0.634781 | 5.230784 | 0.022191 |
| 4453_at | YHR072W | *ERG7* | 3.851902 | 6.153053 | 0.626015 | 5.177691 | 0.022879 |
| 4065_at | YIR029W | *DAL2* | 4.852402 | 6.953453 | 0.697841 | 5.055486 | 0.024548 |
| 6266_at | YDR209C | *---* | 4.852402 | 7.353654 | 0.659863 | 5.048423 | 0.024648 |
| 4077_at | YIR042C | *---* | 4.652302 | 7.453704 | 0.62416 | 5.023775 | 0.025002 |
| 10494_at | YKR069W | *MET1* | 8.254104 | 16.75836 | 0.492537 | 4.988824 | 0.025512 |
| 8437_at | YOR100C | *CRC1* | 7.053504 | 9.554755 | 0.738219 | 4.981874 | 0.025614 |
| 7096_at | YBR278W | *DPB3* | 5.052503 | 6.853403 | 0.737225 | 4.980828 | 0.02563 |
| 9706_at | YML050W | *---* | 5.052503 | 6.753353 | 0.748147 | 4.980356 | 0.025637 |
| 5762_at | YEL028W | *---* | 4.952452 | 7.253604 | 0.682758 | 4.940075 | 0.026241 |
| 5778_at | YEL057C | *---* | 6.553253 | 9.354655 | 0.700534 | 4.721655 | 0.029785 |
| 4388_at | YHR139C | *SPS100* | 12.15606 | 0.05 | 243.1211 | 4.716587 | 0.029873 |
| 9344_at | YMR294W | *JNM1* | 4.652302 | 6.053003 | 0.768594 | 4.711825 | 0.029956 |
| 4585_at | non-annotated | *---* | 4.752352 | 7.253604 | 0.655171 | 4.700211 | 0.030159 |
| 6666_at | YDL197C | *ASF2* | 7.353654 | 5.252603 | 1.400002 | 4.668415 | 0.030722 |
| 10071_at | YLR266C | *PDR8* | 4.852402 | 6.953453 | 0.697841 | 4.66482 | 0.030787 |
| 4076_f_at | YIR041W | *PAU3* | 6.353153 | 8.554254 | 0.742689 | 4.591705 | 0.032127 |
| 2213_at | --- | *---* | 6.753353 | 9.954955 | 0.678391 | 4.563206 | 0.032666 |
| 8566_at | YOR005C | *DNL4* | 5.352653 | 6.753353 | 0.792592 | 4.538282 | 0.033145 |
| 10811_at | non-annotated | *---* | 4.252102 | 5.652803 | 0.752211 | 4.522667 | 0.033449 |
| 11092_at | YJL065C | *DLS1* | 5.752853 | 7.853904 | 0.732483 | 4.515431 | 0.03359 |
| 11025_at | YJR003C | *---* | 3.951952 | 5.652803 | 0.699114 | 4.416671 | 0.035589 |
| 7796_at | YPL047W | *SGF11* | 4.052002 | 5.752853 | 0.704347 | 4.348701 | 0.037037 |
| 4997_at | YGR024C | *THG1* | 4.452202 | 7.153554 | 0.622376 | 4.337251 | 0.037287 |
| 6320_at | YDR174W | *HMO1* | 5.552753 | 7.253604 | 0.765516 | 4.30248 | 0.038057 |
| 8780_at | YNR068C | *---* | 7.053504 | 27.66381 | 0.254972 | 4.293026 | 0.038269 |
| 4158_at | YIL059C | *---* | 3.751852 | 5.652803 | 0.663715 | 4.260572 | 0.039007 |
| 6118_at | YDR332W | *---* | 4.152052 | 5.952953 | 0.697478 | 4.249705 | 0.039257 |
| 6253_at | YDR242W | *AMD2* | 4.152052 | 5.852903 | 0.7094 | 4.224753 | 0.039838 |
| 4878_at | YGR133W | *PEX4* | 5.252603 | 7.153554 | 0.734265 | 4.221568 | 0.039913 |
| 7661_at | YPR085C | *---* | 4.652302 | 3.351652 | 1.388063 | 4.205078 | 0.040303 |
| 8629_at | YOL068C | *HST1* | 4.852402 | 3.551752 | 1.3662 | 4.191932 | 0.040617 |
| 5297_at | YGL263W | *COS12* | 4.252102 | 6.853403 | 0.620437 | 4.153467 | 0.041549 |
| 3477_at | YBRWDELTA17 | *---* | 4.252102 | 7.753854 | 0.548386 | 4.085174 | 0.043261 |
| 5539_at | YER039C-A | *---* | 5.352653 | 7.853904 | 0.681528 | 4.07841 | 0.043435 |
| 10119_at | YLR227C | *ADY4* | 4.552252 | 6.353153 | 0.716534 | 4.03087 | 0.044675 |
| 8717_at | YOL164W | *---* | 6.153053 | 8.054004 | 0.763974 | 4.01184 | 0.045182 |
| 4627_at | non-annotated | *---* | 3.651802 | 5.352653 | 0.682242 | 4.006465 | 0.045326 |
| 8346_at | YOR190W | *SPR1* | 5.552753 | 7.453704 | 0.744966 | 4.001374 | 0.045463 |
| 8881_at | YNL014W | *HEF3* | 6.053003 | 7.953954 | 0.761006 | 3.997051 | 0.04558 |
| 10048_at | YLR288C | *MEC3* | 5.052503 | 6.453203 | 0.782945 | 3.963082 | 0.046508 |
| 10503_at | YKR078W | *---* | 3.751852 | 5.152553 | 0.728154 | 3.959779 | 0.0466 |
| 5954_at | YDR530C | *APA2* | 6.653303 | 9.154555 | 0.726775 | 3.945564 | 0.046995 |
| 6911_at | YCL016C | *DCC1* | 5.252603 | 7.053504 | 0.74468 | 3.937386 | 0.047224 |
| 4378_at | YHR129C | *ARP1* | 3.251602 | 5.052503 | 0.643563 | 3.916162 | 0.047824 |
| 7127_at | YBR217W | *ATG12* | 4.552252 | 6.753353 | 0.674073 | 3.888107 | 0.048629 |
| 5270_at | YGL243W | *TAD1* | 4.152052 | 5.752853 | 0.721738 | 3.817711 | 0.050713 |
| 7623_at | YPR135W | *CTF4* | 4.452202 | 5.952953 | 0.747898 | 3.809165 | 0.050973 |
| 7505_at | non-annotated | *---* | 4.152052 | 5.552753 | 0.747747 | 3.806701 | 0.051048 |
| 10154_at | YLR174W | *IDP2* | 5.052503 | 6.253103 | 0.807999 | 3.792057 | 0.051496 |
| 6797_at | YCR102C | *---* | 7.953954 | 11.55576 | 0.688311 | 3.77995 | 0.05187 |
| 8526_at | YOR009W | *TIR4* | 5.452703 | 7.053504 | 0.773049 | 3.77911 | 0.051896 |
| 6794_at | YCR099C | *---* | 5.752853 | 7.553754 | 0.761589 | 3.750239 | 0.0528 |
| 4283_at | YIL168W | *---* | 6.653303 | 10.05501 | 0.661691 | 3.7473 | 0.052893 |
| 8042_at | non-annotated | *---* | 5.452703 | 8.054004 | 0.677018 | 3.713137 | 0.053986 |
| 10317_at | YLR021W | *---* | 5.252603 | 7.053504 | 0.74468 | 3.697511 | 0.054494 |
| 8120_at | non-annotated | *---* | 3.451702 | 5.652803 | 0.610618 | 3.680213 | 0.055062 |
| 5153_at | YGL136C | *MRM2* | 4.252102 | 6.053003 | 0.702478 | 3.67693 | 0.05517 |
| 10413_at | YLL063C | *AYT1* | 5.452703 | 7.053504 | 0.773049 | 3.669279 | 0.055424 |
| 4262_i_at | non-annotated | *---* | 4.952452 | 6.753353 | 0.733332 | 3.598378 | 0.057836 |
| 7323_at | YBR055C | *PRP6* | 4.452202 | 6.053003 | 0.735536 | 3.561775 | 0.059124 |
| 5259_at | YGL254W | *FZF1* | 5.852903 | 7.653804 | 0.764705 | 3.544596 | 0.05974 |
| 6188_at | YDR268W | *MSW1* | 4.452202 | 5.852903 | 0.760683 | 3.519192 | 0.060662 |
| 8553_at | YOL009C | *MDM12* | 4.252102 | 6.453203 | 0.658913 | 3.498413 | 0.061428 |
| 4340_at | YHR180W | *---* | 6.353153 | 8.254104 | 0.769696 | 3.395906 | 0.065358 |
| 6050_at | YDR399W | *HPT1* | 2.851401 | 4.452202 | 0.640447 | 3.395145 | 0.065389 |
| 8159_at | non-annotated | *---* | 4.452202 | 6.253103 | 0.711999 | 3.392442 | 0.065496 |
| 4400_at | YHR150W | *PEX28* | 5.152553 | 6.653303 | 0.774435 | 3.383999 | 0.065832 |
| 4313_at | YHR199C | *---* | 5.552753 | 7.153554 | 0.776223 | 3.373313 | 0.06626 |
| 8785_at | YNR074C | *AIF1* | 6.153053 | 7.453704 | 0.825503 | 3.367951 | 0.066476 |
| 10186_s_at | YLR156W | *---* | 6.753353 | 9.854905 | 0.685278 | 3.346751 | 0.067338 |
| 7903_at | YPL167C | *REV3* | 3.951952 | 6.053003 | 0.652891 | 3.337627 | 0.067712 |
| 6230_at | YDR265W | *PEX10* | 6.453203 | 8.454204 | 0.763313 | 3.290494 | 0.069682 |
| 10724_at | YKL149C | *DBR1* | 5.352653 | 6.653303 | 0.804511 | 3.278383 | 0.070198 |
| 6205_at | YDR285W | *ZIP1* | 5.952953 | 7.853904 | 0.757961 | 3.253221 | 0.071283 |
| 4083_at | YIR002C | *MPH1* | 4.852402 | 6.553253 | 0.740457 | 3.245978 | 0.071599 |
| 7892_at | YPL133C | *RDS2* | 4.952452 | 7.653804 | 0.647058 | 3.241925 | 0.071776 |
| 8694_at | YOL140W | *ARG8* | 4.652302 | 6.653303 | 0.699247 | 3.234836 | 0.072088 |
| 7498_at | non-annotated | *---* | 6.253103 | 7.853904 | 0.796178 | 3.214302 | 0.072997 |
| 5917_at | YDR538W | *PAD1* | 5.952953 | 7.653804 | 0.777777 | 3.191343 | 0.074029 |
| 9745_at | YML099C | *ARG81* | 6.553253 | 5.152553 | 1.271846 | 3.179542 | 0.074566 |
| 6466_at | YDR005C | *MAF1* | 6.253103 | 8.654304 | 0.722543 | 3.16696 | 0.075142 |
| 9134_at | YNL260C | *---* | 7.853904 | 6.553253 | 1.198474 | 3.166714 | 0.075154 |
| 4724_i_at | YGR122C-A | *---* | 4.252102 | 5.852903 | 0.726495 | 3.104661 | 0.078069 |
| 4224_at | YIL132C | *CSM2* | 5.252603 | 6.953453 | 0.755395 | 3.104622 | 0.07807 |
| 8249_at | YOR313C | *SPS4* | 5.352653 | 7.353654 | 0.72789 | 3.090877 | 0.078732 |
| 4823_at | YGR168C | *---* | 3.851902 | 5.552753 | 0.693692 | 3.039822 | 0.081245 |
| 5143_at | YGL146C | *---* | 6.953453 | 9.054505 | 0.767955 | 3.004372 | 0.08304 |
| 3828_f_at | YKRCDELTA11 | *---* | 3.351652 | 5.152553 | 0.650484 | 2.993564 | 0.083596 |
| 7111_at | YBR246W | *---* | 3.951952 | 5.752853 | 0.686955 | 2.991061 | 0.083725 |
| 5898_at | non-annotated | *---* | 4.352152 | 5.652803 | 0.76991 | 2.970114 | 0.084816 |
| 4358_at | YHR153C | *SPO16* | 4.952452 | 6.953453 | 0.712229 | 2.966514 | 0.085005 |
| 7609_at | YPR123C | *---* | 5.952953 | 8.554254 | 0.695906 | 2.949025 | 0.085929 |
| 6921_at | YCL056C | *---* | 4.452202 | 6.153053 | 0.723576 | 2.945664 | 0.086108 |
| 11372_at | YAL037W | *---* | 4.852402 | 6.453203 | 0.751937 | 2.907241 | 0.088183 |
| 3850_at | YJRWDELTA20 | *---* | 4.752352 | 6.453203 | 0.736433 | 2.906421 | 0.088227 |
| 5101_at | YGL096W | *TOS8* | 5.852903 | 8.154054 | 0.717791 | 2.89647 | 0.088774 |
| 10581_at | YKL026C | *GPX1* | 8.454204 | 13.65681 | 0.619047 | 2.888813 | 0.089197 |
| 10067_at | YLR263W | *RED1* | 4.152052 | 5.352653 | 0.7757 | 2.885284 | 0.089392 |
| 10375_at | YLL055W | *---* | 15.65781 | 29.06451 | 0.538726 | 2.885177 | 0.089398 |
| 6267_at | YDR210W | *---* | 4.752352 | 6.353153 | 0.748031 | 2.882217 | 0.089563 |
| 7549_g_at | YPR196W | *MAL33* | 5.952953 | 7.853904 | 0.757961 | 2.878371 | 0.089777 |
| 5404_at | YFL026W | *STE2* | 5.752853 | 7.453704 | 0.771811 | 2.870788 | 0.090201 |
| 10361_f_at | YLL025W | *DAN2* | 6.953453 | 9.154555 | 0.759562 | 2.86747 | 0.090387 |
| 8240_at | YOR305W | *---* | 5.252603 | 3.851902 | 1.363639 | 2.846917 | 0.091549 |
| 5454_at | non-annotated | *---* | 8.654304 | 5.752853 | 1.50435 | 2.841447 | 0.091861 |
| 4505_at | YHR031C | *RRM3* | 4.952452 | 6.853403 | 0.722627 | 2.832394 | 0.092381 |
| 8704_at | YOL130W | *ALR1* | 7.253604 | 5.452703 | 1.330277 | 2.796347 | 0.094479 |
| 6826_at | YCR036W | *RBK1* | 5.652803 | 6.953453 | 0.812949 | 2.771103 | 0.09598 |
| 6995_at | non-annotated | *---* | 3.851902 | 5.152553 | 0.747572 | 2.742215 | 0.097729 |
| 6057_at | YDR406W | *PDR15* | 8.454204 | 4.352152 | 1.942534 | 2.726153 | 0.098717 |
| 3894_s_at | --- | *---* | 10.15506 | 7.553754 | 1.344372 | 2.71349 | 0.099503 |
| 8544_at | YOR026W | *BUB3* | 4.552252 | 6.053003 | 0.752065 | 2.700862 | 0.100294 |
| 8112_at | non-annotated | *---* | 4.252102 | 5.452703 | 0.779816 | 2.644289 | 0.103923 |
| 10340_at | YLL003W | *SFI1* | 3.751852 | 4.852402 | 0.773195 | 2.641654 | 0.104095 |
| 6045_at | YDR438W | *---* | 5.852903 | 4.652302 | 1.258066 | 2.639633 | 0.104228 |
| 4361_at | YHR156C | *LIN1* | 4.952452 | 7.053504 | 0.702127 | 2.616461 | 0.10576 |
| 5478_i_at | non-annotated | *---* | 4.652302 | 7.953954 | 0.584904 | 2.610026 | 0.10619 |
| 6474_at | YDR013W | *PSF1* | 5.052503 | 6.753353 | 0.748147 | 2.568576 | 0.109006 |
| 5854_at | non-annotated | *---* | 5.452703 | 8.054004 | 0.677018 | 2.561784 | 0.109475 |
| 3540_at | YPRCTAU3 | *---* | 3.051502 | 5.452703 | 0.559631 | 2.554137 | 0.110006 |
| 10080_at | YLR233C | *EST1* | 5.152553 | 6.353153 | 0.811023 | 2.53952 | 0.111029 |
| 6202_at | YDR282C | *---* | 5.052503 | 6.553253 | 0.770991 | 2.539128 | 0.111056 |
| 10854_at | YJR152W | *DAL5* | 7.153554 | 5.552753 | 1.28829 | 2.520146 | 0.1124 |
| 6165_at | YDR291W | *---* | 4.952452 | 6.153053 | 0.804877 | 2.518376 | 0.112526 |
| 4079_at | YIL003W | *CFD1* | 4.552252 | 5.852903 | 0.777777 | 2.482452 | 0.115123 |
| 4563_f_at | YHL046C | *PAU4* | 5.552753 | 6.853403 | 0.810218 | 2.465407 | 0.116378 |
| 4078_i_at | YIR043C | *---* | 4.652302 | 8.354154 | 0.556885 | 2.461085 | 0.116698 |
| 6891_at | YCR014C | *POL4* | 4.352152 | 5.552753 | 0.783783 | 2.453822 | 0.117239 |
| 10483_at | YKR102W | *FLO10* | 4.852402 | 6.353153 | 0.763779 | 2.438288 | 0.118405 |
| 5396_at | YFL034C-A | *RPL22B* | 3.451702 | 4.952452 | 0.696968 | 2.417573 | 0.119981 |
| 8832_at | YNR029C | *---* | 4.852402 | 5.852903 | 0.829059 | 2.383045 | 0.122658 |
| 5264_at | YGL249W | *ZIP2* | 5.552753 | 7.553754 | 0.735098 | 2.3706 | 0.12364 |
| 8492_at | YOR064C | *YNG1* | 4.552252 | 5.652803 | 0.805309 | 2.356572 | 0.124756 |
| 4573_at | YHL035C | *---* | 7.253604 | 16.25811 | 0.446153 | 2.312722 | 0.128319 |
| 8064_at | non-annotated | *---* | 6.153053 | 8.154054 | 0.7546 | 2.286105 | 0.130537 |
| 5812_at | YEL072W | *RMD6* | 12.25611 | 21.36066 | 0.57377 | 2.273612 | 0.131593 |
| 5453_at | non-annotated | *---* | 4.652302 | 6.153053 | 0.756097 | 2.239212 | 0.13455 |
| 3152_at | --- | *---* | 5.152553 | 12.15606 | 0.423867 | 2.230102 | 0.135345 |
| 9987_at | YLR318W | *EST2* | 3.951952 | 5.052503 | 0.782177 | 2.226218 | 0.135686 |
| 6875_at | YCL004W | *PGS1* | 7.453704 | 6.153053 | 1.211383 | 2.213509 | 0.136807 |
| 8719_at | YOL162W | *---* | 6.853403 | 8.554254 | 0.801169 | 2.205717 | 0.1375 |
| 4622_at | non-annotated | *---* | 5.952953 | 4.652302 | 1.279571 | 2.204676 | 0.137593 |
| 8903_at | YNL038W | *GPI15* | 3.551752 | 5.252603 | 0.676189 | 2.190538 | 0.138861 |
| 6214_at | YDR249C | *---* | 3.751852 | 5.252603 | 0.714284 | 2.170384 | 0.140691 |
| 8499_at | YOR072W | *---* | 7.353654 | 10.15506 | 0.724137 | 2.165415 | 0.141146 |
| 5696_at | YER041W | *YEN1* | 5.052503 | 6.153053 | 0.821137 | 2.164621 | 0.141219 |
| 8131_at | non-annotated | *---* | 5.652803 | 7.153554 | 0.790209 | 2.163359 | 0.141335 |
| 7382_at | YBL025W | *RRN10* | 6.353153 | 4.952452 | 1.28283 | 2.149424 | 0.142623 |
| 4270_f_at | non-annotated | *---* | 5.952953 | 7.853904 | 0.757961 | 2.146306 | 0.142913 |
| 3683_f_at | YMRWDEL16 | *---* | 3.451702 | 4.652302 | 0.741934 | 2.143913 | 0.143136 |
| 9237_at | non-annotated | *---* | 4.452202 | 5.952953 | 0.747898 | 2.130634 | 0.144381 |
| 6440_at | YDR026C | *---* | 4.152052 | 5.252603 | 0.790475 | 2.128465 | 0.144586 |
| 8777_at | YNR065C | *---* | 4.852402 | 3.951952 | 1.22785 | 2.123016 | 0.145101 |
| 5347_at | YFR005C | *SAD1* | 4.852402 | 6.353153 | 0.763779 | 2.108973 | 0.146438 |
| 5533_at | YER184C | *---* | 6.053003 | 7.653804 | 0.790849 | 2.060765 | 0.151134 |
| 5362_at | YFR020W | *---* | 6.253103 | 7.853904 | 0.796178 | 2.059465 | 0.151263 |
| 9865_f_at | YLR461W | *PAU4* | 5.752853 | 6.953453 | 0.827338 | 2.034733 | 0.153741 |
| 10312_at | YLR016C | *---* | 6.153053 | 7.553754 | 0.814569 | 2.033615 | 0.153854 |
| 5872_at | non-annotated | *---* | 4.252102 | 5.652803 | 0.752211 | 2.020454 | 0.155193 |
| 6281_at | YDR179W-A | *---* | 3.651802 | 5.052503 | 0.722771 | 1.97846 | 0.159553 |
| 4968_at | YGR042W | *---* | 4.252102 | 5.252603 | 0.809523 | 1.974851 | 0.159934 |
| 8179_at | YOR380W | *RDR1* | 4.452202 | 5.352653 | 0.831775 | 1.934908 | 0.164222 |
| 10189_at | YLR164W | *---* | 7.153554 | 10.55526 | 0.677724 | 1.9349 | 0.164223 |
| 7478_s_at | YBL109W | *---* | 5.452703 | 6.653303 | 0.819548 | 1.933098 | 0.16442 |
| 4280_f_at | YIL176C | *PAU4* | 5.752853 | 7.153554 | 0.804195 | 1.929759 | 0.164785 |
| 4582_at | YHL026C | *---* | 3.551752 | 4.352152 | 0.816091 | 1.926717 | 0.165118 |
| 9786_at | non-annotated | *---* | 5.652803 | 6.953453 | 0.812949 | 1.890881 | 0.169103 |
| 7133_at | YBR223C | *TDP1* | 6.653303 | 8.254104 | 0.80606 | 1.889161 | 0.169297 |
| 7102_at | YBR237W | *PRP5* | 5.252603 | 4.352152 | 1.206898 | 1.881027 | 0.170217 |
| 10743_at | YKL176C | *LST4* | 6.453203 | 7.953954 | 0.81132 | 1.835283 | 0.175505 |
| 9785_at | non-annotated | *---* | 4.952452 | 6.153053 | 0.804877 | 1.835088 | 0.175528 |
| 3150_at | --- | *---* | 6.153053 | 12.45621 | 0.493975 | 1.820541 | 0.177249 |
| 9365_at | YMR271C | *URA10* | 8.254104 | 6.753353 | 1.222223 | 1.806291 | 0.178954 |
| 4738_at | YGR262C | *BUD32* | 5.052503 | 6.453203 | 0.782945 | 1.791744 | 0.180714 |
| 9157_at | YNL282W | *POP3* | 7.153554 | 5.552753 | 1.28829 | 1.789918 | 0.180936 |
| 4067_at | YIR031C | *DAL7* | 4.452202 | 5.852903 | 0.760683 | 1.755243 | 0.185219 |
| 4236_at | YIL120W | *QDR1* | 4.652302 | 6.053003 | 0.768594 | 1.755084 | 0.185239 |
| 5621_at | YER092W | *IES5* | 6.853403 | 8.354154 | 0.820359 | 1.749463 | 0.185944 |
| 6745_at | non-annotated | *---* | 4.552252 | 6.153053 | 0.739836 | 1.736575 | 0.187573 |
| 4650_f_at | non-annotated | *---* | 5.852903 | 7.453704 | 0.785234 | 1.723067 | 0.189299 |
| 10869_at | non-annotated | *---* | 4.452202 | 5.252603 | 0.847618 | 1.722557 | 0.189364 |
| 8736_at | YNL138W-A | *YSF3* | 6.253103 | 7.653804 | 0.816993 | 1.716289 | 0.190171 |
| 7343_at | YBR030W | *---* | 4.252102 | 5.352653 | 0.794392 | 1.695477 | 0.192881 |
| 5765_at | YEL025C | *---* | 5.252603 | 6.353153 | 0.826771 | 1.654686 | 0.198322 |
| 4305_at | non-annotated | *---* | 3.451702 | 4.552252 | 0.75824 | 1.6516 | 0.198741 |
| 2714_s_at | --- | *---* | 9.154555 | 7.153554 | 1.279721 | 1.632999 | 0.201289 |
| 5186_at | YGL193C | *---* | 4.552252 | 5.552753 | 0.819819 | 1.624575 | 0.202455 |
| 8994_at | YNL128W | *TEP1* | 6.453203 | 7.853904 | 0.821655 | 1.622916 | 0.202686 |
| 7566_at | YPR168W | *NUT2* | 9.154555 | 7.453704 | 1.228189 | 1.61036 | 0.204441 |
| 6465_at | YDR004W | *RAD57* | 4.552252 | 5.452703 | 0.834862 | 1.599217 | 0.206014 |
| 2107_at | --- | *---* | 4.752352 | 5.852903 | 0.811965 | 1.594241 | 0.206721 |
| 5786_f_at | YEL049W | *PAU4* | 6.353153 | 7.453704 | 0.852348 | 1.589017 | 0.207467 |
| 8066_at | non-annotated | *---* | 5.652803 | 6.853403 | 0.824817 | 1.587913 | 0.207625 |
| 7990_at | YPL214C | *THI6* | 6.853403 | 7.953954 | 0.861635 | 1.571417 | 0.210001 |
| 4322_at | YHR210C | *---* | 5.252603 | 6.553253 | 0.801526 | 1.543791 | 0.214054 |
| 6222_at | YDR257C | *SET7* | 6.153053 | 5.152553 | 1.194176 | 1.533036 | 0.215657 |
| 10379_at | YLL051C | *FRE6* | 7.353654 | 8.654304 | 0.849711 | 1.521083 | 0.217456 |
| 6798_f_at | YCR104W | *PAU3* | 7.253604 | 8.654304 | 0.83815 | 1.514614 | 0.218436 |
| 10827_s_at | non-annotated | *---* | 7.653804 | 6.353153 | 1.204725 | 1.467519 | 0.225738 |
| 4156_at | YIL061C | *SNP1* | 4.552252 | 5.552753 | 0.819819 | 1.462348 | 0.226557 |
| 5410_f_at | YFL020C | *PAU5* | 6.453203 | 7.953954 | 0.81132 | 1.452164 | 0.228181 |
| 6693_at | YDL215C | *GDH2* | 6.253103 | 7.953954 | 0.786163 | 1.448359 | 0.228791 |
| 5632_at | YER101C | *AST2* | 5.152553 | 6.253103 | 0.823999 | 1.419757 | 0.233443 |
| 10070_at | YLR265C | *NEJ1* | 4.752352 | 5.652803 | 0.840707 | 1.41544 | 0.234155 |
| 8578_at | YOL029C | *---* | 5.352653 | 6.153053 | 0.869918 | 1.409044 | 0.235215 |
| 8896_at | YNL046W | *---* | 5.852903 | 7.153554 | 0.818181 | 1.365202 | 0.242638 |
| 7825_at | YPL064C | *CWC27* | 4.452202 | 5.352653 | 0.831775 | 1.359994 | 0.243538 |
| 7074_f_at | YBR301W | *PAU4* | 5.952953 | 6.953453 | 0.856115 | 1.343405 | 0.246434 |
| 5649_at | YER076C | *---* | 4.652302 | 5.452703 | 0.85321 | 1.339973 | 0.247039 |
| 5020_at | YGR003W | *CUL3* | 4.752352 | 6.053003 | 0.785123 | 1.338603 | 0.247281 |
| 8230_at | YOR298W | *MUM3* | 6.153053 | 7.153554 | 0.860139 | 1.301961 | 0.253855 |
| 6964_s_at | non-annotated | *---* | 6.153053 | 7.553754 | 0.814569 | 1.300544 | 0.254114 |
| 4945_at | YGR064W | *---* | 4.752352 | 5.952953 | 0.798318 | 1.299819 | 0.254246 |
| 6800_at | YCR106W | *RDS1* | 9.654805 | 7.253604 | 1.331036 | 1.282414 | 0.257451 |
| 10832_s_at | non-annotated | *---* | 7.053504 | 6.053003 | 1.16529 | 1.272659 | 0.259268 |
| 3555_s_at | --- | *---* | 8.354154 | 6.553253 | 1.27481 | 1.268248 | 0.260096 |
| 9290_f_at | YMR325W | *PAU3* | 6.953453 | 8.254104 | 0.842424 | 1.2626 | 0.26116 |
| 9595_at | YMR065W | *KAR5* | 4.152052 | 5.052503 | 0.821781 | 1.261717 | 0.261326 |
| 7923_at | YPL147W | *PXA1* | 5.552753 | 4.852402 | 1.144331 | 1.260638 | 0.26153 |
| 4832_at | YGR177C | *ATF2* | 4.352152 | 5.452703 | 0.798164 | 1.252303 | 0.263113 |
| 6479_at | YDR018C | *---* | 6.453203 | 8.354154 | 0.772454 | 1.246249 | 0.26427 |
| 6914_at | YCL011C | *GBP2* | 5.752853 | 6.553253 | 0.877862 | 1.234638 | 0.266507 |
| 10306_at | YLR010C | *TEN1* | 5.452703 | 6.653303 | 0.819548 | 1.227721 | 0.267851 |
| 6437_at | YDR022C | *CIS1* | 4.852402 | 5.852903 | 0.829059 | 1.223737 | 0.268629 |
| 9619_at | YMR041C | *---* | 7.553754 | 6.053003 | 1.247935 | 1.222019 | 0.268965 |
| 6962_at | non-annotated | *---* | 6.653303 | 7.953954 | 0.836477 | 1.22012 | 0.269337 |
| 9800_s_at | YML133C | *YRF1* | 7.853904 | 10.85541 | 0.723502 | 1.218727 | 0.269611 |
| 4626_at | non-annotated | *---* | 4.952452 | 5.752853 | 0.860869 | 1.200325 | 0.273257 |
| 4613_at | non-annotated | *---* | 4.452202 | 5.252603 | 0.847618 | 1.192438 | 0.274838 |
| 6088_at | YDR391C | *---* | 5.952953 | 6.853403 | 0.868613 | 1.190686 | 0.275191 |
| 3900_f_at | YJLWTY4-1 | *---* | 4.952452 | 5.852903 | 0.846153 | 1.184908 | 0.276359 |
| 9731_at | YML068W | *ITT1* | 6.653303 | 5.852903 | 1.136753 | 1.182218 | 0.276905 |
| 7545_at | YPR193C | *HPA2* | 6.653303 | 8.454204 | 0.786982 | 1.178393 | 0.277683 |
| 8182_at | YOR383C | *FIT3* | 7.153554 | 5.052503 | 1.415844 | 1.166092 | 0.280206 |
| 8037_at | YPL258C | *THI21* | 5.452703 | 6.453203 | 0.844961 | 1.160138 | 0.281437 |
| 4143_at | YIL028W | *---* | 6.653303 | 8.254104 | 0.80606 | 1.15172 | 0.283189 |
| 10249_at | YLR085C | *ARP6* | 6.353153 | 7.353654 | 0.863945 | 1.150452 | 0.283454 |
| 3541_at | YPRWTAU4 | *---* | 7.053504 | 5.452703 | 1.293579 | 1.146475 | 0.284288 |
| 6621_at | YDL154W | *MSH5* | 5.852903 | 7.053504 | 0.829787 | 1.14472 | 0.284657 |
| 8073_f_at | non-annotated | *---* | 8.154054 | 10.85541 | 0.751152 | 1.139916 | 0.28567 |
| 7138_at | YBR228W | *SLX1* | 4.952452 | 5.752853 | 0.860869 | 1.129194 | 0.287947 |
| 3556_s_at | --- | *---* | 7.153554 | 5.552753 | 1.28829 | 1.114105 | 0.291191 |
| 4639_f_at | non-annotated | *---* | 5.152553 | 6.753353 | 0.762962 | 1.103722 | 0.293451 |
| 8395_at | YOR148C | *SPP2* | 6.253103 | 7.153554 | 0.874125 | 1.09971 | 0.29433 |
| 5323_at | YFR026C | *---* | 4.652302 | 5.552753 | 0.837837 | 1.089451 | 0.296593 |
| 7210_at | YBR163W | *DEM1* | 4.152052 | 4.952452 | 0.838383 | 1.086593 | 0.297227 |
| 4562_at | YHL047C | *ARN2* | 6.553253 | 5.452703 | 1.201836 | 1.081834 | 0.298288 |
| 3893_s_at | --- | *---* | 9.554755 | 7.753854 | 1.232259 | 1.048608 | 0.305828 |
| 7436_at | YBL060W | *---* | 5.052503 | 4.352152 | 1.16092 | 1.047846 | 0.306004 |
| 10195_at | YLR122C | *---* | 6.053003 | 7.153554 | 0.846153 | 1.024294 | 0.311503 |
| 3336_f_at | YERCTAU2 | *---* | 7.653804 | 5.852903 | 1.307694 | 1.01819 | 0.312949 |
| 9488_at | YMR135C | *GID8* | 5.952953 | 6.953453 | 0.856115 | 1.018142 | 0.31296 |
| 4317_at | YHR204W | *MNL1* | 3.651802 | 4.552252 | 0.802197 | 1.014548 | 0.313816 |
| 7311_at | YBR043C | *QDR3* | 4.852402 | 5.852903 | 0.829059 | 1.009854 | 0.314938 |
| 3791_s_at | YLL067C | *YRF1* | 7.453704 | 9.554755 | 0.780104 | 1.009282 | 0.315075 |
| 4448_at | YHR067W | *HTD2* | 4.352152 | 5.352653 | 0.813083 | 1.006646 | 0.315708 |
| 6643_at | YDL177C | *---* | 4.252102 | 4.852402 | 0.876288 | 1.001862 | 0.31686 |
| 5253_g_at | YGL259W | *YPS6* | 11.25561 | 8.554254 | 1.31579 | 1.000054 | 0.317298 |
| 8218_at | YOR328W | *PDR10* | 6.053003 | 5.252603 | 1.152382 | 0.997062 | 0.318022 |
| 5509_at | non-annotated | *---* | 5.352653 | 6.353153 | 0.842519 | 0.99191 | 0.319276 |
| 10224_at | YLR105C | *SEN2* | 5.052503 | 5.852903 | 0.863247 | 0.980561 | 0.32206 |
| 10416_at | YLL060C | *GTT2* | 7.953954 | 17.15856 | 0.463556 | 0.974796 | 0.323487 |
| 10433_at | non-annotated | *---* | 5.552753 | 6.453203 | 0.860465 | 0.965662 | 0.325765 |
| 3901_f_at | YJLWTAU2 | *---* | 4.652302 | 5.452703 | 0.85321 | 0.960426 | 0.32708 |
| 9526_at | YMR127C | *SAS2* | 3.851902 | 4.652302 | 0.827956 | 0.956972 | 0.327951 |
| 7922_at | YPL148C | *PPT2* | 4.452202 | 5.352653 | 0.831775 | 0.954788 | 0.328504 |
| 8488_at | YOR060C | *---* | 8.054004 | 6.553253 | 1.229009 | 0.949715 | 0.329792 |
| 6795_at | YCR100C | *---* | 8.854404 | 7.353654 | 1.204082 | 0.94381 | 0.3313 |
| 4308_at | non-annotated | *---* | 5.552753 | 6.753353 | 0.822222 | 0.923129 | 0.336655 |
| 8456_at | YOR119C | *RIO1* | 5.852903 | 4.852402 | 1.206187 | 0.92007 | 0.337457 |
| 6709_i_at | YDL247W | *MPH2* | 6.853403 | 7.853904 | 0.872611 | 0.917247 | 0.338199 |
| 7847_at | YPL088W | *---* | 5.752853 | 5.052503 | 1.138615 | 0.905385 | 0.341342 |
| 8783_at | YNR071C | *---* | 6.453203 | 6.953453 | 0.928057 | 0.904062 | 0.341695 |
| 8701_at | YOL133W | *HRT1* | 7.353654 | 6.753353 | 1.088889 | 0.892967 | 0.344675 |
| 6112_at | YDR370C | *---* | 3.751852 | 4.652302 | 0.806451 | 0.884313 | 0.347023 |
| 3151_g_at | --- | *---* | 7.153554 | 13.45671 | 0.531598 | 0.880115 | 0.34817 |
| 7068_at | YBR295W | *PCA1* | 7.053504 | 6.053003 | 1.16529 | 0.857015 | 0.354575 |
| 4683_at | YGR169C-A | *---* | 6.053003 | 5.252603 | 1.152382 | 0.84768 | 0.357209 |
| 6519_at | YDL033C | *SLM3* | 6.653303 | 5.852903 | 1.136753 | 0.843404 | 0.358425 |
| 2822_at | --- | *---* | 8.454204 | 10.65531 | 0.793427 | 0.839367 | 0.359578 |
| 5600_at | YER115C | *SPR6* | 4.752352 | 5.452703 | 0.871559 | 0.833645 | 0.361221 |
| 5515_at | non-annotated | *---* | 3.551752 | 2.951451 | 1.203392 | 0.827595 | 0.362969 |
| 5787_at | YEL048C | *---* | 6.553253 | 5.552753 | 1.180181 | 0.824138 | 0.363973 |
| 6746_at | non-annotated | *---* | 5.952953 | 7.053504 | 0.843971 | 0.820237 | 0.365111 |
| 9450_at | YMR180C | *CTL1* | 6.353153 | 5.652803 | 1.123894 | 0.818318 | 0.365672 |
| 4955_at | YGR074W | *SMD1* | 5.352653 | 6.253103 | 0.855999 | 0.803845 | 0.369946 |
| 8909_at | YNL032W | *SIW14* | 4.852402 | 5.552753 | 0.873873 | 0.799642 | 0.3712 |
| 8718_at | YOL163W | *---* | 7.853904 | 8.854404 | 0.887005 | 0.798977 | 0.371399 |
| 7153_at | YBR197C | *---* | 6.653303 | 5.652803 | 1.176992 | 0.791082 | 0.373773 |
| 3332_i_at | YERWDELTA11 | *---* | 5.052503 | 7.153554 | 0.706293 | 0.789403 | 0.374281 |
| 9100_at | YNL249C | *MPA43* | 5.152553 | 4.552252 | 1.131869 | 0.788779 | 0.37447 |
| 6946_at | --- | *---* | 6.553253 | 5.852903 | 1.119659 | 0.784577 | 0.375745 |
| 10819_at | non-annotated | *---* | 5.352653 | 6.353153 | 0.842519 | 0.777862 | 0.377796 |
| 4682_at | YGR169C-A | *---* | 5.152553 | 4.352152 | 1.183909 | 0.777032 | 0.37805 |
| 10030_at | YLR315W | *NKP2* | 7.253604 | 6.253103 | 1.160001 | 0.769017 | 0.380521 |
| 4938_at | YGR057C | *LST7* | 5.052503 | 5.952953 | 0.848739 | 0.766181 | 0.381401 |
| 7286_at | YBR063C | *---* | 5.852903 | 5.152553 | 1.135923 | 0.753977 | 0.38522 |
| 6756_at | YCR020W-B | *HTL1* | 4.152052 | 5.352653 | 0.7757 | 0.72072 | 0.395908 |
| 3899_s_at | YJLWTY4-1 | *---* | 5.752853 | 4.952452 | 1.161617 | 0.711021 | 0.399105 |
| 8362_at | YOR205C | *---* | 3.451702 | 4.052002 | 0.851851 | 0.702305 | 0.40201 |
| 10031_at | YLR316C | *TAD3* | 5.552753 | 6.353153 | 0.874015 | 0.694429 | 0.404662 |
| 5423_s_at | YFL057C | *AAD16* | 5.752853 | 6.753353 | 0.851851 | 0.68912 | 0.406464 |
| 9234_at | non-annotated | *---* | 6.253103 | 7.053504 | 0.886524 | 0.688526 | 0.406666 |
| 10374_at | YLL056C | *---* | 7.153554 | 8.254104 | 0.866666 | 0.687608 | 0.406979 |
| 4112_at | YIL013C | *PDR11* | 4.752352 | 5.452703 | 0.871559 | 0.677625 | 0.410406 |
| 5601_at | YER116C | *SLX8* | 7.053504 | 6.153053 | 1.146342 | 0.667472 | 0.413934 |
| 9813_at | non-annotated | *---* | 7.653804 | 9.054505 | 0.845303 | 0.665344 | 0.41468 |
| 3306_i_at | YGLWDELTA4 | *---* | 5.352653 | 4.552252 | 1.175825 | 0.654766 | 0.418414 |
| 5424_at | YFL056C | *AAD6* | 8.854404 | 10.55526 | 0.838862 | 0.649797 | 0.420185 |
| 8487_at | YOR059C | *---* | 5.352653 | 6.053003 | 0.884297 | 0.649213 | 0.420394 |
| 9299_at | non-annotated | *---* | 7.253604 | 6.353153 | 1.141733 | 0.648325 | 0.420712 |
| 5512_at | non-annotated | *---* | 4.152052 | 5.052503 | 0.821781 | 0.646025 | 0.421537 |
| 6193_at | YDR273W | *DON1* | 6.453203 | 7.553754 | 0.854304 | 0.643013 | 0.422622 |
| 10478_at | YKR097W | *PCK1* | 5.252603 | 5.852903 | 0.897435 | 0.626667 | 0.42858 |
| 8038_at | YPL257W | *---* | 6.053003 | 6.653303 | 0.909774 | 0.623731 | 0.429664 |
| 9621_at | YML002W | *---* | 5.652803 | 5.052503 | 1.118812 | 0.621734 | 0.430404 |
| 4100_at | YIR019C | *MUC1* | 5.952953 | 6.853403 | 0.868613 | 0.621081 | 0.430646 |
| 8720_f_at | YOL161C | *PAU4* | 5.852903 | 6.453203 | 0.906976 | 0.62061 | 0.430821 |
| 6714_at | YDL239C | *ADY3* | 5.252603 | 4.552252 | 1.153847 | 0.615415 | 0.432756 |
| 10572_at | YKR009C | FOX2 | 6.353153 | 6.953453 | 0.913669 | 0.604404 | 0.436903 |
| 5273_at | YGL240W | *DOC1* | 8.254104 | 9.954955 | 0.829145 | 0.593026 | 0.441251 |
| 5160_at | YGL128C | *CWC23* | 6.353153 | 7.053504 | 0.900709 | 0.588948 | 0.442826 |
| 6018_at | YDR459C | *---* | 4.452202 | 5.052503 | 0.881188 | 0.577998 | 0.447098 |
| 10814_s_at | non-annotated | *---* | 5.152553 | 4.452202 | 1.157304 | 0.573772 | 0.448764 |
| 9285_at | YMR320W | *---* | 6.453203 | 5.652803 | 1.141594 | 0.560797 | 0.453939 |
| 9600_at | YMR023C | *MSS1* | 5.752853 | 6.353153 | 0.905511 | 0.554036 | 0.456673 |
| 4651_at | non-annotated | *---* | 4.152052 | 4.852402 | 0.855669 | 0.543636 | 0.46093 |
| 10199_at | YLR125W | *---* | 7.653804 | 8.854404 | 0.864406 | 0.540173 | 0.462361 |
| 9379_at | YMR240C | *CUS1* | 5.052503 | 5.552753 | 0.90991 | 0.534475 | 0.464731 |
| 6046_at | YDR439W | *LRS4* | 5.252603 | 5.752853 | 0.913043 | 0.528512 | 0.467233 |
| 10842_at | non-annotated | *---* | 7.153554 | 8.054004 | 0.888198 | 0.527538 | 0.467644 |
| 5518_at | non-annotated | *---* | 5.552753 | 6.353153 | 0.874015 | 0.508624 | 0.475735 |
| 6691_at | YDL217C | *TIM22* | 4.652302 | 5.152553 | 0.902912 | 0.494523 | 0.481917 |
| 5389_at | YFL044C | *---* | 7.653804 | 7.053504 | 1.085107 | 0.484123 | 0.486561 |
| 6084_at | YDR387C | *---* | 5.252603 | 5.852903 | 0.897435 | 0.444776 | 0.504826 |
| 4966_at | YGR040W | *KSS1* | 6.153053 | 5.552753 | 1.108109 | 0.425849 | 0.514033 |
| 3554_f_at | YPLCTY4-1 | *---* | 7.353654 | 6.353153 | 1.157481 | 0.420724 | 0.516576 |
| 4653_at | non-annotated | *---* | 4.952452 | 5.652803 | 0.876106 | 0.414625 | 0.519631 |
| 3553_f_at | YPLCTAU1 | *---* | 4.452202 | 5.152553 | 0.864077 | 0.39003 | 0.532283 |
| 6562_at | YDL080C | *THI3* | 5.152553 | 5.552753 | 0.927928 | 0.387714 | 0.533504 |
| 4652_at | non-annotated | *---* | 4.752352 | 5.352653 | 0.88785 | 0.386211 | 0.534298 |
| 7548_at | YPR196W | *---* | 5.952953 | 5.352653 | 1.11215 | 0.377258 | 0.539074 |
| 9082_at | YNL221C | *POP1* | 5.452703 | 4.952452 | 1.101011 | 0.370668 | 0.542641 |
| 9145_at | YNL294C | *RIM21* | 5.852903 | 6.253103 | 0.936 | 0.356728 | 0.550329 |
| 5715_at | YER015W | *FAA2* | 4.852402 | 5.352653 | 0.906542 | 0.350635 | 0.553754 |
| 6805_at | YCR063W | *BUD31* | 5.852903 | 5.252603 | 1.114286 | 0.341594 | 0.558911 |
| 10432_at | non-annotated | *---* | 5.252603 | 5.652803 | 0.929203 | 0.338094 | 0.560932 |
| 8733_at | non-annotated | *---* | 5.052503 | 5.652803 | 0.893805 | 0.336438 | 0.561892 |
| 10482_at | YKR101W | *SIR1* | 4.652302 | 5.052503 | 0.920792 | 0.335763 | 0.562285 |
| 7690_at | YPR070W | *MED1* | 5.552753 | 5.052503 | 1.09901 | 0.334111 | 0.563248 |
| 9201_at | YNL329C | *PEX6* | 6.153053 | 5.752853 | 1.069566 | 0.33045 | 0.565394 |
| 2698_s_at | Saccharomyces | *---* | 7.753854 | 8.954454 | 0.865921 | 0.313976 | 0.57525 |
| 3686_f_at | YMRCDELTA18 | *---* | 5.452703 | 4.852402 | 1.123712 | 0.300247 | 0.583727 |
| 6774_at | YCL004W | *PGS1* | 7.153554 | 6.753353 | 1.059259 | 0.290019 | 0.590209 |
| 8818_at | YNR059W | *MNT4* | 5.052503 | 5.452703 | 0.926605 | 0.287901 | 0.591569 |
| 5862_at | non-annotated | *---* | 6.253103 | 6.853403 | 0.912408 | 0.286093 | 0.592735 |
| 4056_at | YIR020C | *---* | 6.153053 | 7.053504 | 0.87234 | 0.282899 | 0.594807 |
| 5693_at | YER038C | *KRE29* | 6.953453 | 7.553754 | 0.92053 | 0.281946 | 0.595429 |
| 10567_at | YKR004C | *ECM9* | 4.952452 | 5.452703 | 0.908256 | 0.269627 | 0.603582 |
| 4117_at | YIL009C-A | *EST3* | 5.052503 | 5.452703 | 0.926605 | 0.266443 | 0.605728 |
| 3670_f_at | YNRCTAU3 | *---* | 5.452703 | 5.952953 | 0.915966 | 0.263331 | 0.607841 |
| 10196_g_at | YLR122C | *---* | 7.153554 | 7.853904 | 0.910828 | 0.26243 | 0.608455 |
| 5809_i_at | YEL074W | *---* | 6.153053 | 6.653303 | 0.924812 | 0.262171 | 0.608632 |
| 8348_i_at | YOR192C | *---* | 5.652803 | 6.153053 | 0.918699 | 0.257221 | 0.612036 |
| 3626_i_at | YNR075W | *COS10* | 5.752853 | 5.252603 | 1.095239 | 0.255806 | 0.613016 |
| 9072_at | YNL187W | *---* | 6.153053 | 6.553253 | 0.938931 | 0.25248 | 0.615334 |
| 5089_at | YGL063W | *PUS2* | 4.452202 | 4.852402 | 0.917525 | 0.251965 | 0.615695 |
| 10775_at | YKL188C | *PXA2* | 7.553754 | 7.053504 | 1.070922 | 0.25166 | 0.615909 |
| 10437_at | non-annotated | *---* | 5.652803 | 6.153053 | 0.918699 | 0.248475 | 0.618151 |
| 5529_at | YER180C | *ISC10* | 4.852402 | 4.452202 | 1.089888 | 0.241021 | 0.62347 |
| 5637_at | YER066W | *---* | 5.052503 | 4.652302 | 1.086022 | 0.240689 | 0.623709 |
| 3583_i_at | YPLCDELTA1 | *---* | 6.953453 | 5.852903 | 1.188035 | 0.237237 | 0.626209 |
| 7866_at | YPL114W | *---* | 7.353654 | 7.953954 | 0.924528 | 0.23385 | 0.628683 |
| 10412_f_at | YLL064C | *PAU* | 5.752853 | 6.253103 | 0.92 | 0.233065 | 0.629261 |
| 10353_at | YLL033W | *---* | 6.053003 | 6.553253 | 0.923664 | 0.22853 | 0.632616 |
| 11169_at | YJL126W | *NIT2* | 7.653804 | 8.554254 | 0.894737 | 0.224842 | 0.635375 |
| 8564_at | YOR003W | *YSP3* | 6.653303 | 6.953453 | 0.956834 | 0.219633 | 0.63932 |
| 4771_at | YGR251W | *---* | 4.652302 | 5.052503 | 0.920792 | 0.217487 | 0.640962 |
| 3149_at | Saccharomyces | *---* | 9.554755 | 11.85591 | 0.805907 | 0.213409 | 0.644109 |
| 7776_at | YPL022W | *RAD1* | 5.752853 | 6.153053 | 0.934959 | 0.206331 | 0.649659 |
| 11247_s_at | YJL225C | *YRF1* | 6.653303 | 6.153053 | 1.081301 | 0.199359 | 0.655239 |
| 6963_at | non-annotated | *---* | 6.653303 | 7.253604 | 0.917241 | 0.199334 | 0.655259 |
| 8925_at | YNL063W | *---* | 5.152553 | 4.952452 | 1.040404 | 0.197357 | 0.656863 |
| 8786_f_at | YNR076W | *PAU4* | 5.652803 | 5.952953 | 0.94958 | 0.189817 | 0.663069 |
| 9861_at | YLR457C | *NBP1* | 4.652302 | 4.952452 | 0.939394 | 0.183913 | 0.668032 |
| 4544_at | YHL018W | *---* | 8.054004 | 7.153554 | 1.125875 | 0.180958 | 0.670551 |
| 6945_g_at | Saccharomyces | *---* | 7.153554 | 7.553754 | 0.94702 | 0.179004 | 0.67223 |
| 6650_at | YDL170W | *UGA3* | 5.752853 | 5.452703 | 1.055046 | 0.178224 | 0.672904 |
| 10686_at | YKL098W | *---* | 5.752853 | 5.452703 | 1.055046 | 0.175547 | 0.675229 |
| 6710_at | YDL243C | *AAD16* | 7.153554 | 6.653303 | 1.075188 | 0.170324 | 0.679824 |
| 6608_at | YDL123W | *SNA4* | 7.153554 | 7.753854 | 0.92258 | 0.166269 | 0.683449 |
| 3617_f_at | YORWTAU2 | *---* | 6.753353 | 6.153053 | 1.097561 | 0.162729 | 0.686656 |
| 10968_at | YJR039W | *---* | 6.753353 | 7.053504 | 0.957447 | 0.162114 | 0.687218 |
| 9867_s_at | YLR465C | *---* | 7.853904 | 8.654304 | 0.907514 | 0.15515 | 0.693662 |
| 10485_at | YKR104W | *---* | 6.353153 | 6.853403 | 0.927007 | 0.154887 | 0.693908 |
| 3301_f_at | YGLWTAU1 | *---* | 5.652803 | 5.252603 | 1.076191 | 0.15145 | 0.697153 |
| 4116_at | YIL009C-A | *EST3* | 4.552252 | 4.852402 | 0.938144 | 0.151283 | 0.697312 |
| 7596_at | YPR153W | *---* | 7.953954 | 7.353654 | 1.081633 | 0.146804 | 0.701609 |
| 5636_at | YER065C | *ICL1* | 5.252603 | 5.552753 | 0.945946 | 0.145142 | 0.703222 |
| 9073_at | YNL230C | *ELA1* | 5.352653 | 5.652803 | 0.946902 | 0.144734 | 0.703619 |
| 7757_at | YPR005C | *HAL1* | 5.852903 | 5.552753 | 1.054054 | 0.14469 | 0.703662 |
| 3907_f_at | YAL068C | *PAU4* | 6.253103 | 6.553253 | 0.954198 | 0.144645 | 0.703706 |
| 8071_f_at | non-annotated | *---* | 8.554254 | 7.953954 | 1.075472 | 0.140466 | 0.707818 |
| 9668_at | YML042W | *CAT2* | 6.753353 | 7.053504 | 0.957447 | 0.140409 | 0.707875 |
| 8609_at | YOL043C | *NTG2* | 5.252603 | 5.652803 | 0.929203 | 0.134757 | 0.71355 |
| 7088_at | YBR270C | *---* | 3.751852 | 3.951952 | 0.949367 | 0.118463 | 0.730708 |
| 8023_at | YPL272C | *---* | 4.952452 | 4.752352 | 1.042105 | 0.117727 | 0.731513 |
| 10792_at | YKL220C | *FRE2* | 5.452703 | 5.752853 | 0.947826 | 0.112149 | 0.737711 |
| 5929_at | non-annotated | *---* | 4.852402 | 4.552252 | 1.065934 | 0.108399 | 0.741975 |
| 7919_at | YPL151C | *PRP46* | 5.952953 | 5.752853 | 1.034783 | 0.108225 | 0.742174 |
| 3625_at | YNR075W | *COS10* | 7.453704 | 8.154054 | 0.91411 | 0.105485 | 0.745345 |
| 5679_at | YER024W | *YAT2* | 5.852903 | 6.153053 | 0.951219 | 0.099551 | 0.752369 |
| 8676_at | YOL112W | *MSB4* | 5.252603 | 5.452703 | 0.963303 | 0.097963 | 0.754288 |
| 3542_f_at | YPR204W | *YRF1* | 5.852903 | 6.153053 | 0.951219 | 0.092419 | 0.761124 |
| 3691_f_at | YMRCTAU3 | *---* | 6.753353 | 6.353153 | 1.062992 | 0.089142 | 0.765271 |
| 6862_at | YCR028C | *FEN2* | 6.053003 | 5.752853 | 1.052174 | 0.088653 | 0.765896 |
| 10812_s_at | non-annotated | *---* | 5.052503 | 5.252603 | 0.961905 | 0.086757 | 0.768341 |
| 5638_g_at | YER066W | *CDC4* | 5.852903 | 5.652803 | 1.035398 | 0.084448 | 0.771358 |
| 11385_s_at | YAL063C | *FLO5* | 6.553253 | 6.253103 | 1.048 | 0.078301 | 0.779613 |
| 10372_at | YLL016W | *SDC25* | 7.553754 | 7.253604 | 1.041379 | 0.072164 | 0.788211 |
| 6263_at | YDR206W | *EBS1* | 5.852903 | 6.053003 | 0.966942 | 0.071334 | 0.789404 |
| 7940_at | YPL175W | *SPT14* | 4.352152 | 4.552252 | 0.956044 | 0.068131 | 0.794077 |
| 7921_at | YPL149W | *ATG5* | 4.652302 | 4.852402 | 0.958763 | 0.057402 | 0.810651 |
| 8209_at | YOR319W | *HSH49* | 5.552753 | 5.752853 | 0.965217 | 0.055506 | 0.813745 |
| 2923_at | YKR103/104W | *NFT1* | 7.153554 | 6.853403 | 1.043796 | 0.053488 | 0.817101 |
| 6149_at | YDR318W | *MCM21* | 5.152553 | 5.352653 | 0.962617 | 0.04864 | 0.825447 |
| 10998_at | YJR021C | *REC107* | 7.053504 | 7.353654 | 0.959184 | 0.0471 | 0.828188 |
| 7607_at | YPR121W | *THI22* | 4.152052 | 4.252102 | 0.97647 | 0.04702 | 0.828332 |
| 10288_at | YLR036C | *---* | 5.152553 | 5.352653 | 0.962617 | 0.044097 | 0.833673 |
| 4988_at | YGR015C | *---* | 4.652302 | 4.552252 | 1.021978 | 0.038926 | 0.843595 |
| 3792_s_at | YLL066C | *YRF1* | 5.752853 | 5.952953 | 0.966386 | 0.038465 | 0.844513 |
| 4362_at | YHR157W | *REC104* | 6.753353 | 6.553253 | 1.030534 | 0.037368 | 0.846718 |
| 3895_s_at | --- | *---* | 7.453704 | 7.253604 | 1.027586 | 0.028562 | 0.865793 |
| 10853_at | YJR151C | *DAN4* | 7.653804 | 7.453704 | 1.026846 | 0.025654 | 0.872748 |
| 4556_at | YHL006C | *SHU1* | 6.053003 | 6.153053 | 0.98374 | 0.02356 | 0.878009 |
| 10197_at | YLR123C | *---* | 8.054004 | 8.254104 | 0.975758 | 0.023172 | 0.879011 |
| 4148_at | YIL071C | *PCI8* | 6.253103 | 6.153053 | 1.01626 | 0.022604 | 0.880491 |
| 6195_at | YDR275W | *BSC2* | 4.452202 | 4.652302 | 0.956989 | 0.022027 | 0.882015 |
| 10539_at | YKR022C | *---* | 6.353153 | 6.253103 | 1.016 | 0.021918 | 0.882307 |
| 9471_at | YMR159C | *ATG16* | 8.854404 | 9.054505 | 0.9779 | 0.0165 | 0.897792 |
| 3183_f_at | YIL177C | *YRF1* | 7.053504 | 7.253604 | 0.972414 | 0.014768 | 0.903277 |
| 8044_at | non-annotated | *---* | 5.952953 | 6.153053 | 0.96748 | 0.014497 | 0.904162 |
| 5810_s_at | YEL074W | *---* | 6.453203 | 6.553253 | 0.984733 | 0.014426 | 0.904397 |
| 7567_at | YPR169W | *---* | 7.053504 | 6.953453 | 1.014389 | 0.013265 | 0.908308 |
| 7479_at | YBL107C | *---* | 4.752352 | 4.852402 | 0.979381 | 0.011635 | 0.914103 |
| 9735_at | YML107C | *---* | 5.752853 | 5.852903 | 0.982906 | 0.011519 | 0.914528 |
| 6217_at | YDR252W | *BTT1* | 6.153053 | 6.253103 | 0.984 | 0.011409 | 0.914936 |
| 6402_at | YDR078C | *SHU2* | 5.252603 | 5.352653 | 0.981308 | 0.010483 | 0.91845 |
| 4075_at | YIR039C | *YPS6* | 7.753854 | 7.553754 | 1.02649 | 0.010107 | 0.919921 |
| 5262_at | YGL251C | *HFM1* | 5.752853 | 5.852903 | 0.982906 | 0.009369 | 0.922892 |
| 8165_at | YOR366W | *---* | 4.752352 | 4.852402 | 0.979381 | 0.007594 | 0.930559 |
| 6406_at | YDR082W | *STN1* | 4.152052 | 4.052002 | 1.024692 | 0.006308 | 0.936696 |
| 9849_at | non-annotated | *---* | 5.752853 | 5.652803 | 1.017699 | 0.006096 | 0.937766 |
| 7057_at | YBR284W | *---* | 5.952953 | 6.053003 | 0.983471 | 0.004259 | 0.947967 |
| 3982_at | KANMX4 | *---* | 5.152553 | 5.352653 | 0.962617 | 0.004036 | 0.949343 |
| 5883_i_at | non-annotated | *---* | 6.653303 | 6.753353 | 0.985185 | 0.003915 | 0.950111 |
| 4263_at | non-annotated | *NHR013C* | 8.354154 | 8.254104 | 1.012121 | 0.002787 | 0.9579 |
| 10484_at | YKR103W | *NFT1* | 7.553754 | 7.753854 | 0.974193 | 0.001189 | 0.972492 |
| 6947_g_at | Saccharomyces | *---* | 6.653303 | 6.653303 | 1 | 2.00E-14 | 1 |
| 8202_at | YOR358W | *HAP5* | 5.852903 | 5.852903 | 1 | 0 | 1 |
| 8239_at | YOR304C-A | *---* | 5.352653 | 5.352653 | 1 | 0 | 1 |
| 8419_at | YOR127W | *RGA1* | 4.952452 | 4.952452 | 1 | 0 | 1 |
| 4090_at | YIR009W | *MSL1* | 5.152553 | 5.152553 | 1 | 0 | 1 |
| 6106_at | YDR364C | *CDC40* | 4.852402 | 4.852402 | 1 | -6.00E-15 | 1 |
| 7066_at | YBR293W | *VBA2* | 5.152553 | 5.152553 | 1 | 0 | 1 |
| 8774_at | YNL024C-A | *---* | 5.652803 | 5.652803 | 1 | 0 | 1 |
| 3684_f_at | YMRWTAU2 | *---* | 4.752352 | 4.752352 | 1 | 0 | 1 |
| 3374_at | YERWDELTA9 | *---* | 7.253604 | 7.253604 | 1 | 0 | 1 |
| 3480_at | YBRCDELTA18 | *---* | 5.752853 | 5.752853 | 1 | 0 | 1 |
| 7016_at | non-annotated | *---* | 5.152553 | 5.152553 | 1 | -6.00E-15 | 1 |

^a^Red = Direct targets Blue = Indirect targets
