# Supplementary material for: Impact of Nonsense-Mediated mRNA Decay on the Global Expression Profile of Budding Yeast
Source: PLoS Genet. 2006 Nov 24;2(11):e203. doi: 10.1371/journal.pgen.0020203 (PMC1657058; doi:10.1371/journal.pgen.0020203)
Supplement: Table S3 — (35 KB DOC) [file pgen.0020203.st003.doc]

| Table S3. Negative NMD-sensitive probe sets selected by SAMProbe Sets^a^ ORF Gene mut t1/2 wt t1/2 FCR -2lnλ  *p*-value | | | | | | | |
| --- | --- | --- | --- | --- | --- | --- | --- |
| 11137_at | YJL110C | GZF3 | 15.3577 | 3.8519 | 3.98703 | 293385 | 5.2E-07 |
| 11184_at | YJL154C | *VPS35* | 10.3552 | 5.35265 | 1.93458 | 182.226 | 0.00125 |
| 11207_at | YJL176C | *SWI3* | 14.2571 | 6.053 | 2.35538 | 120.162 | 0.00197 |
| 11136_at | YJL111W | *CCT7* | 14.6573 | 5.95295 | 2.46219 | 71.9266 | 0.00345 |
| 11148_at | YJL145W | *SFH5* | 18.2591 | 8.054 | 2.26708 | 36.612 | 0.00729 |
| 11060_at | YJL052W | *TDH1* | 16.3582 | 6.6533 | 2.45865 | 28.0908 | 0.0098 |
| 11194_at | YJL186W | *MNN5* | 9.95495 | 4.95245 | 2.01011 | 20.9019 | 0.01367 |
| 11350_at | YAL017W | *PSK1* | 15.1576 | 7.8539 | 1.92994 | 13.3363 | 0.02284 |
| 3073_s_at | --- | *CYR1* | 13.8569 | 6.55325 | 2.11451 | 12.5271 | 0.02454 |
| 9271_at | --- | *---* | 3.4517 | 5.75285 | 0.6 | 11.9848 | 0.02583 |
| 10925_at | YJR086W | *STE18* | 11.2556 | 7.35365 | 1.53061 | 3.66945 | 0.10686 |
| 3951_at | --- | *---* | 7.8539 | 5.2526 | 1.49524 | 1.47894 | 0.37633 |
| 3953_at | --- | *---* | 0.4502 | 4.6523 | 0.09677 | 1.23144 | 0.51876 |
| 3954_at | --- | *---* | 6.75335 | 3.6518 | 1.84932 | 1.09765 | 0.66598 |
| 2836_g_at | --- | *FKS1* | 10.4552 | 9.2546 | 1.12973 | 1.05682 | 0.73954 |
| 3952_at | --- | *---* | 1.95095 | 5.55275 | 0.35135 | 1.04407 | 0.76898 |
| 9566_at | YMR080C | *NAM7* | 7.2536 | 6.15305 | 1.17886 | 1.02452 | 0.82578 |
| 9762_at | YML123C | *PHO84* | 1.15055 | 6.053 | 0.19008 | 1.01045 | 0.88534 |

^a^Red = Direct targets Blue = Indirect targets
