# Supplementary material for: Impact of Nonsense-Mediated mRNA Decay on the Global Expression Profile of Budding Yeast
Source: PLoS Genet. 2006 Nov 24;2(11):e203. doi: 10.1371/journal.pgen.0020203 (PMC1657058; doi:10.1371/journal.pgen.0020203)
Supplement: Table S4 — (52 KB DOC) [file pgen.0020203.st004.doc]

| **Table S4.** Stable transcripts used to generate a reference line  Probe ID Gene Probe ID Gene Probe ID Gene | | | | | |
| --- | --- | --- | --- | --- | --- |
| 10019_at | *ACO1* | 10721_at | *GPM1* | 4194_at | *PRM5* |
| 11356_at | *ACS1* | 4074_at | *GTT1* | 6513_at | *PRM7* |
| 7748_at | *AEP3* | 4576_at | *GUT1* | 11350_at | *PSK1* |
| 10228_at | *AHP1* | 8881_at | *HEF3* | 6446_at | *PST2* |
| 5179_at | *AMS1* | 10143_at | *HMX1* | 4584_at | *RIM4* |
| 6499_at | *APC11* | 4432_at | *HXT5* | 5812_at | *RMD6* |
| 4386_at | *ARO9* | 11258_at | *HXT8* | 7192_at | *RPL21B* |
| 8433_at | *ARP8* | 8886_at | *IDP3* | 7918_at | *RRD2* |
| 11213_at | *ASG7* | 8642_at | *IZH4* | 5589_at | *RTT105* |
| 9107_at | *ATG2* | 10930_at | *JSN1* | 7124_at | *SDS24* |
| 11046_at | *BBC1* | 11078_at | *KAR2* | 11142_at | *SET4* |
| 11388_at | *BDH1* | 10681_at | *LAP4* | 6222_at | *SET7* |
| 9329_at | *CAT8* | 6909_at | *LEU2* | 5627_at | *SHC1* |
| 11371_at | *CDC19* | 4519_at | *LEU5* | 8888_at | *SIS1* |
| 6314_at | *CDC37* | 4881_at | *LSB1* | 5368_at | *SMX2* |
| 11103_at | *CHS6* | 10723_at | *MCR1* | 4526_at | *SOD2* |
| 8849_at | *CIT1* | 7859_at | *MEI5* | 8883_at | *SPO1* |
| 6261_at | *COQ4* | 4534_at | *MIP6* | 5218_at | *SPT16* |
| 9721_at | *CPR3* | 10731_at | *MRP8* | 10470_at | *SRL3* |
| 4784_at | *CRM1* | 8672_at | *MSN1* | 10362_at | *SSA2* |
| 4224_at | *CSM2* | 5055_at | *MST27* | 9950_at | *SSQ1* |
| 7958_at | *CSM4* | 7289_at | *NRG2* | 8229_at | *TIM18* |
| 6483_at | *DIA3* | 11352_at | *NTG1* | 9355_at | *TPS3* |
| 6193_at | *DON1* | 9444_at | *PAI3* | 5036_at | *TRP5* |
| 5760_at | *ECM10* | 8880_at | *PBI2* | 4774_at | *TRX2* |
| 4495_at | *ECM12* | 10208_at | *PDC5* | 9743_at | *TSL1* |
| 4714_at | *ERV29* | 4923_at | *PDC6* | 8188_at | *TYE7* |
| 7339_at | *ETR1* | 7916_at | *PEP4* | 10802_at | *UBA1* |
| 7282_at | *FES1* | 10518_at | *PET10* | 7686_at | *UBA3* |
| 7308_at | *FIG1* | 4204_at | *PFK26* | 10392_at | *UBI4* |
| 4478_at | *FSH1* | 6890_at | *PGK1* | 10479_at | *UBP11* |
| 8412_at | *GCY1* | 8659_at | *PHM7* | 7928_at | *UIP4* |
| 6099_at | *GGA1* | 8408_at | *PNS1* | 11098_at | *YHC3* |
| 6937_at | *GLK1* | 7214_at | *POP7* | 10690_at | *YJU2* |
| 4732_at | *GND2* | 8932_at | *POR2* | 5704_at | *YND1* |
| 6485_at | *GPD1* | 11140_at | *PRM10* | 7184_at | *YPC1* |
| 4075_at | *YPS6* | 7322_at | *YRO2* |  |  |
