# Supplementary material for: Impact of Nonsense-Mediated mRNA Decay on the Global Expression Profile of Budding Yeast
Source: PLoS Genet. 2006 Nov 24;2(11):e203. doi: 10.1371/journal.pgen.0020203 (PMC1657058; doi:10.1371/journal.pgen.0020203)
Supplement: Table S5 — (30 KB DOC) [file pgen.0020203.st005.doc]

**Table S5. Performance assessments of decay models using simulated data^a^**

| Model | Criterion | M1 best | M2 best | M3 best | M4 best |
| --- | --- | --- | --- | --- | --- |
| 1 | AIC | 391 | 366 | 41 | 202 |
|  | BIC | 496 | 332 | 16 | 156 |
| 2 | AIC | 10 | 895 | 8 | 87 |
|  | BIC | 19 | 922 | 6 | 53 |
| 3 | AIC | 37 | 49 | 893 | 21 |
|  | BIC | 56 | 53 | 878 | 13 |
| 4 | AIC | 176 | 570 | 27 | 227 |
|  | BIC | 261 | 557 | 9 | 173 |

^a^When data were simulated from model k (k=1, 2, 3, 4), model k can be identified as the best model fitting the data most of the time. Parameters in simulated data were , , , , and . When data were simulated from model 4, model 2 was identified as the best model. Model 2 tolerates the data from Model 1 or Model 4 better than Model 1 or 4 tolerates the data from Model 2, suggesting that Model 2 is the best choice.
