# Supplementary material for: Impact of Nonsense-Mediated mRNA Decay on the Global Expression Profile of Budding Yeast
Source: PLoS Genet. 2006 Nov 24;2(11):e203. doi: 10.1371/journal.pgen.0020203 (PMC1657058; doi:10.1371/journal.pgen.0020203)
Supplement: Table S6 — (23 KB DOC) [file pgen.0020203.st006.doc]

**Table S6. Performance assessments of decay models using actual data**

| Strain | Criterion | M1 best | M2 best | M3 best | M4 best |
| --- | --- | --- | --- | --- | --- |
| Nmd^+^ | AIC | 77 | 359 | 4 | 167 |
|  | BIC | 140 | 388 | 2 | 77 |
| Nmd^-^ | AIC | 46 | 232 | 5 | 324 |
|  | BIC | 117 | 326 | 2 | 162 |
