# Supplementary material for: Impact of Nonsense-Mediated mRNA Decay on the Global Expression Profile of Budding Yeast
Source: PLoS Genet. 2006 Nov 24;2(11):e203. doi: 10.1371/journal.pgen.0020203 (PMC1657058; doi:10.1371/journal.pgen.0020203)
Supplement: Table S7 — (43 KB DOC) [file pgen.0020203.st007.doc]

| **Table S7.** Supplementary details on mRNA half-life experiments^a^ | | | | | | | | | | | | |
| --- | --- | --- | --- | --- | --- | --- | --- | --- | --- | --- | --- | --- |
|  | Half-life in NMD^+^ strain (min) | | | | | | Half-life in Nmd^-^ strain (min) | | | | | |
| Transcript | #1 | #2 | #3 | #4 | Avg. | SD | #1 | #2 | #3 | #4 | Avg. | SD |
|  |  |  |  |  |  |  |  |  |  |  |  |  |
| PET18 | 7 | 5 | **3** |  | 5.0 | 2.0 | 25 | 23 | **20** |  | 22.7 | 2.5 |
| FZF1 | **2** | 2 | 3 | 2 | 2.1 | 0.6 | **12** | 8 | 18 | 11 | 12.3 | 4.0 |
| PDR8 | 14 | 15 | **9** |  | 12.7 | 3.2 | 26 | 23 | **18** |  | 22.3 | 4.0 |
| PDR3 | **11** | 10 | 11 |  | 10.7 | 0.6 | **27** | 24 | 26 |  | 25.7 | 1.5 |
| INO4 | 3 | **5** | 2 |  | 3.3 | 1.5 | 9 | **18** | 7 |  | 11.3 | 5.9 |
| ARG81 | 39 | 13 | 31 | **15** | 24.5 | 12.6 | 32 | 17 | 37 | **20** | 26.5 | 9.5 |
| SGF11 | **13** | 12 | 6 |  | 10.3 | 3.8 | **16** | 9 | 9 |  | 11.3 | 4.0 |
| YRR1 | 15 | **7** | 16 |  | 12.7 | 4.9 | 20 | **10** | 14 |  | 14.7 | 5.0 |
| MAF1 | 32 | 34 | **6** |  | 24.0 | 15.6 | 25 | 31 | **8** |  | 21.3 | 11.9 |
| RDR1 | **4** | 5 | 5 |  | 4.7 | 0.6 | **12** | 17 | 10 |  | 13.0 | 3.6 |
| SET7 | 8 | 7 | **7** | 10 | 8.0 | 1.4 | 16 | 16 | **22** | 20 | 18.5 | 3.0 |
| ASF2 | 7 | 4 | **4** |  | 5.0 | 1.7 | 21 | 11 | **11** |  | 14.3 | 5.8 |
| UGA3 | 22 | 27 | **5** |  | 18.0 | 11.5 | 24 | 32 | **6** |  | 20.7 | 13.3 |
| MED1 | 35 | 28 | **3** |  | 22.1 | 16.6 | 30 | 33 | **4** |  | 22.4 | 15.9 |
| SAS2 | 14 | **6** | 38 |  | 19.3 | 16.7 | 12 | **8** | 36 |  | 18.7 | 15.1 |
| RRN10 | **22** | 21 | 22 |  | 21.7 | 0.6 | **22** | 26 | 26 |  | 24.7 | 2.3 |

^a^Trials highlighted in blue were used to generate plots in Figure 2, 3, and 4.
