# Supplementary material for: Impact of Nonsense-Mediated mRNA Decay on the Global Expression Profile of Budding Yeast
Source: PLoS Genet. 2006 Nov 24;2(11):e203. doi: 10.1371/journal.pgen.0020203 (PMC1657058; doi:10.1371/journal.pgen.0020203)
Supplement: Table S8 — (32 KB DOC) [file pgen.0020203.st008.doc]

Table S8. Entrez Gene accession numbers^a^

| Gene | accession # | Gene | accession # |
| --- | --- | --- | --- |
| *AAD15* | 853999 | *RRP6* | 854162 |
| *ABP140* | 854414 | *SAS2* | 855157 |
| *ADR1* | 851802 | *SET7* | 851844 |
| *ARG81* | 854874 | *SGF11* | 856060 |
| *ASF2* | 851330 | *SIR1* | 853976 |
| *COS10* | 855812 | *SKI7* | 854243 |
| *COS12* | 852628 | *SPT10* | 853315 |
| *CPA1* | 854479 | *STE2* | 850518 |
| *CTF13* | 855119 | *STE6* | 853671 |
| *DAN3* | 852603 | *SUP 45* | 852440 |
| *EBS1* | 851787 | *SUP35* | 851752 |
| *EST3* | 854806 | *UGA3* | 851384 |
| *FZF1* | 852638 | *unc-54* | 259839 |
| *HHF2* | 855701 | *hUPF1* | 5976 |
| *INO4* | 854042 | *UPF1* | 855104 |
| *LRS4* | 852049 | *UPF2* | 856476 |
| *MAF1* | 851568 | *UPF3* | 852963 |
| *MAL31* | 852601 | *URA3* | 856692 |
| *MAL33* | 852600 | *XRN1* | 852702 |
| *MED1* | 856183 | *YER039C* | 856761 |
| *PAU11* | 852630 | *YER039C-A* | 856762 |
| *PAU18* | 850662 | *YIL164C* | 854642 |
| *PAU20* | 854003 | *YIL165C* | 854641 |
| *PAU6* | 855813 | *YIL167W* | 854639 |
| *PDR3* | 852278 | *YIL168W* | 854638 |
| *PDR8* | 850971 | *YKU80* | 855132 |
| *PET18* | 850382 | *YNL335W* | 855381 |
| *PPR1* | 850701 | *YOL162W* | 854002 |
| *PPT2* | 855955 | *YOL163W* | 854001 |
| *RDR1* | 854562 | *YRF1* | 855377 |
| *RPB1* | 851415 | *YRR1* | 854333 |
| *RRN10* | 852256 |  |  |

^a^http://www.ncbi.nlm.nih.gov/entrez/query.fcgi?db=gene
