# Supplementary material for: Impact of Nonsense-Mediated mRNA Decay on the Global Expression Profile of Budding Yeast
Source: PLoS Genet. 2006 Nov 24;2(11):e203. doi: 10.1371/journal.pgen.0020203 (PMC1657058; doi:10.1371/journal.pgen.0020203)
Supplement: Table S9 — (23 KB DOC) [file pgen.0020203.st009.doc]

**Table S9. Estimating the number of significant probe sets at different levels of FDR^a^**

| Group | FDR=0.5 | FDR=0.3 | FDR=0.2 | FDR=0.1 | FDR=0.05 |
| --- | --- | --- | --- | --- | --- |
| Positive targets | 316 (52%) | 200 (33%) | 145 (24%) | 75 (12%) | 38 (6%) |
| Negative targets | 11 (61%) | 10 (56%) | 10 (56%) | 4 (22%) | 2 (11%) |
| Non-targets | 3197 (37%) | 1749 (20%) | 1056 (12%) | 490 (6%) | 229 (3%) |

^a^The table provides estimates of how many probe sets can be called “significant”, and how many among these are actually “false” using different stringencies. 9335 probe sets were ranked according to their *p*-values, and threshold lines at different FDR levels were drawn. Points below the threshold line all have a *p* values smaller than or equal to the cutoff *p* value. For example, when setting FDR=0.1, 75+490+4=569 probe sets are called significant, while about 569*0.1=56.9 probe sets are mistakenly classified as significant. At this setting, 75 out of 607 (75/607*100%=12%) positive targets have significantly different half-lives in Nmd^+^ and Nmd^-^ strains, while 7.5 (75*0.1) probe sets are mistakenly called significant.
