# Supplementary material for: Impact of Nonsense-Mediated mRNA Decay on the Global Expression Profile of Budding Yeast
Source: PLoS Genet. 2006 Nov 24;2(11):e203. doi: 10.1371/journal.pgen.0020203 (PMC1657058; doi:10.1371/journal.pgen.0020203)
Supplement: Table S10 — (155 KB DOC) [file pgen.0020203.st010.doc]

| Table S10. uORFs in direct targets of NMD Name start stop length transcription start site^a^ AUG context -3 AUG-CAI(r) | | | | | | | | |
| --- | --- | --- | --- | --- | --- | --- | --- | --- |
| YIL165C | -26 | 1 | 27 | n/a |  | CGGTGGATGCTA | T | 0.1343 |
| YGR133W | -59 | -21 | 39 | n/a |  | CGGGTTATGCTG | G | 0.148 |
| YML050W | -94 | -29 | 66 | -14(1) |  | TCGTGCATGCGG | T | 0.1551 |
| YGR294W | -64 | -35 | 30 | n/a |  | TGAGGTATGCGC | G | 0.1864 |
| YHR199C | -8 | 10 | 18 | -10(1) |  | AGGTATATGGAA | T | 0.1882 |
| YGL146C | -80 | -24 | 57 | n/a |  | AATCTTATGAAA | C | 0.1892 |
| YLR174W | -94 | -77 | 18 | n/a |  | AATTGTATGTTA | T | 0.1915 |
| YJR155W | -4 | 23 | 27 | n/a |  | CGGTGAATGGAT | T | 0.1934 |
| YEL028W | -62 | 4 | 66 | n/a |  | GATTGGATGAAG | T | 0.1938 |
| YMR106C | -31 | -20 | 12 | n/a |  | GGACATATGCAC | C | 0.2027 |
| YOR381W | -43 | 71 | 114 | n/a |  | TTGCTGATGAAC | C | 0.2032 |
| YER187W | -41 | -3 | 39 | n/a |  | CGGAGCATGGAA | A | 0.2037 |
| YER186C | -25 | 17 | 42 | n/a |  | TAGTATATGATC | T | 0.2052 |
| YFL050C | -26 | 1 | 27 | n/a |  | ATTTTTATGAGA | T | 0.2096 |
| YKR012C | -47 | -27 | 21 | n/a |  | GAGCTGATGTTG | C | 0.2198 |
| YGL261C | -76 | -35 | 42 | n/a |  | AACTGTATGCTT | T | 0.2199 |
| YGR168C | -25 | -5 | 21 | n/a |  | GAGCTAATGAGA | C | 0.2222 |
| YLR266C | -71 | 25 | 96 | n/a |  | AAGCCCATGACA | C | 0.2288 |
| YOR100C | -83 | 1 | 84 | n/a |  | CGCTCTATGTGT | T | 0.2326 |
| YML023C | -26 | -3 | 24 | n/a |  | CGGCAAATGGTG | C | 0.2446 |
| YBR008C | -65 | -42 | 24 | n/a |  | ATCCTTATGCCG | C | 0.248 |
| YJR003C | -19 | 17 | 36 | n/a |  | AAGTAGATGAAA | T | 0.2555 |
| YNR058W | -44 | -12 | 33 | -36(1) |  | GGGAGGATGTCA | A | 0.257 |
| YDR013W | -16 | 20 | 36 | n/a |  | ACGGCCATGCTC | G | 0.2607 |
| YBR250W | -67 | 32 | 99 | n/a |  | AAGCCCATGTTT | C | 0.2628 |
| YCR020C | -92 | -54 | 39 | -437(1) |  | ACGAATATGAAC | A | 0.2698 |
| YPR123C | -35 | 70 | 105 | n/a |  | TTTTATATGTGG | T | 0.2713 |
| YGR289C | -44 | -15 | 30 | n/a |  | TTTCTGATGCTA | C | 0.276 |
| YFL003C | -5 | 4 | 9 | n/a |  | AACTATATGCAA | T | 0.2775 |
| YIL167W | -4 | 53 | 57 | n/a |  | GCACGAATGAAT | C | 0.2775 |
| YLL063C | -4 | 8 | 12 | n/a |  | AGGTAGATGGAT | T | 0.2836 |
| YDL187C | -41 | -3 | 39 | n/a |  | ATATGGATGCGT | T | 0.284 |
| YOL068C | -79 | -71 | 9 | n/a |  | ATTGCTATGTTG | G | 0.29 |
| YPL066W | -40 | -29 | 12 | n/a |  | TTTTCTATGGGT | T | 0.2905 |
| YOL158C | -14 | -3 | 12 | n/a |  | TCGTGAATGTCT | T | 0.2928 |
| YPR085C | -44 | -6 | 39 | n/a |  | GGTGTGATGTGC | G | 0.2936 |
| YLR318W | -70 | -53 | 18 | n/a |  | CAATGAATGACA | T | 0.3012 |
| YLR288C | -44 | 4 | 48 | -17(1) |  | GAGATGATGAGA | A | 0.3021 |
| YBR184W | -77 | -9 | 69 | n/a |  | GAATTGATGAAA | T | 0.3022 |
| YMR316C-B | -23 | 64 | 87 | n/a |  | CGGATGATGTGT | A | 0.3024 |
| YOR026W | -97 | 20 | 117 | n/a |  | ATCTGCATGTCC | T | 0.3085 |
| YOL134C | -58 | 83 | 141 | n/a |  | GTGCAAATGGAA | C | 0.3088 |
| YOL104C | -52 | -23 | 30 | n/a |  | ATTGTCATGAAC | G | 0.3092 |
| YNL204C | -7 | 14 | 21 | n/a |  | GAATATATGGAC | T | 0.3128 |
| YML099C | -61 | -53 | 9 | n/a |  | GATTTGATGCTT | T | 0.314 |
| YMR114C | -29 | 22 | 51 | n/a |  | TACCTGATGCTA | C | 0.314 |
| YLR097C | -31 | -14 | 18 | n/a |  | CGACACATGTGT | C | 0.3162 |
| YPL164C | -31 | 17 | 48 | n/a |  | GGAAGAATGAAC | A | 0.3192 |
| YLR227C | -46 | 8 | 54 | -184(1) |  | ACATATATGCCC | T | 0.3237 |
| YGL262W | -13 | 8 | 21 | n/a |  | GTCTTCATGTGC | T | 0.325 |
| YHL040C | -38 | 1 | 39 | n/a |  | AAATATATGGAC | T | 0.3277 |
| YDR336W | -82 | -71 | 12 | n/a |  | GTTTTAATGTGG | T | 0.3311 |
| YDR332W | -16 | 23 | 39 | -10(1) |  | GGAGCCATGAGT | G | 0.3317 |
| YDR174W | -37 | 83 | 120 | -23(1) |  | TCTAGGATGTAC | A | 0.3436 |
| YGR024C | -11 | 28 | 39 | n/a |  | TTCCTAATGCGA | C | 0.3462 |
| YIL029C | -44 | -24 | 21 | n/a |  | GTAAGGATGCTG | A | 0.3476 |
| YBL075C | -49 | -23 | 27 | n/a |  | ATGTCAATGTTT | T | 0.3482 |
| YER039C | -94 | -65 | 30 | n/a |  | GACGCTATGTAT | G | 0.3493 |
| YBR253W | -37 | 23 | 60 | -47(1) |  | ATTGTGATGATA | G | 0.3513 |
| YMR294W | -35 | 1 | 36 | n/a |  | TAAGTCATGAGG | G | 0.3529 |
| YOL108C | -67 | -53 | 15 | n/a |  | ATTCCAATGCCA | C | 0.3531 |
| YIR041W | -62 | -18 | 45 | n/a |  | GAGAATATGTTT | A | 0.3578 |
| YIL100W | -4 | 17 | 21 | n/a |  | ATATATATGTAT | T | 0.3601 |
| YOR005C | -65 | -33 | 33 | n/a |  | GTTAATATGTGG | A | 0.367 |
| YLL003W | -79 | 26 | 105 | -57(1) |  | TTGGCAATGGGA | G | 0.3731 |
| YNL254C | -74 | -60 | 15 | -48(1) |  | GTAGCGATGAGA | G | 0.3767 |
| YDR242W | -70 | 62 | 132 | -15(1) |  | TTTTCAATGGGG | T | 0.3768 |
| YIL003W | -46 | 92 | 138 | -426(1) -52(1) -48(1) |  | ATACACATGCGT | C | 0.377 |
| YIR043C | -5 | 52 | 57 | n/a |  | TCTTCAATGCCA | T | 0.3806 |
| YLL057C | -89 | -21 | 69 | n/a |  | TTCTTGATGACA | T | 0.3812 |
| YIL132C | -73 | -62 | 12 | n/a |  | ATTTTAATGTTA | T | 0.3815 |
| YBR217W | -8 | 4 | 12 | n/a |  | GAACCAATGACA | C | 0.3866 |
| YAL037W | -82 | -53 | 30 | n/a |  | AATTAGATGGGA | T | 0.3867 |
| YCL016C | -49 | 35 | 84 | -20(1) |  | TATGAGATGAAC | G | 0.3874 |
| YIL059C | -52 | 77 | 129 | -145(1) -6(1) |  | AACCACATGGGA | C | 0.3885 |
| YLR233C | -22 | 8 | 30 | n/a |  | CATTAAATGACA | T | 0.3949 |
| YDR438W | -35 | -9 | 27 | n/a |  | AGAGTGATGGTA | G | 0.3962 |
| YOL009C | -55 | 11 | 66 | n/a |  | AAAAGCATGCCA | A | 0.397 |
| YHL046C | -71 | -27 | 45 | n/a |  | GAGAATATGTCT | A | 0.3973 |
| YDL218W | -86 | -78 | 9 | -16(1) |  | ACCATTATGCTT | A | 0.4032 |
| YOL014W | -55 | 29 | 84 | n/a |  | GTGAGAATGTCT | A | 0.4047 |
| YNL335W | -53 | 22 | 75 | n/a |  | GTTAACATGAAG | A | 0.4052 |
| YEL023C | -85 | -65 | 21 | n/a |  | AAATTAATGTAC | T | 0.4066 |
| YHR153C | -14 | -3 | 12 | n/a |  | TCTGGAATGGTT | G | 0.4164 |
| YFR054C | -88 | -35 | 54 | n/a |  | TTTTTCATGGCT | T | 0.4202 |
| YPR170C | -7 | 35 | 42 | n/a |  | GAAGAGATGAGC | G | 0.4269 |
| YPR200C | -49 | -17 | 33 | -59(1) -43(1) |  | AAACTAATGTAT | C | 0.4281 |
| YLR165C | -37 | 35 | 72 | -8(1) |  | GTATGAATGGCT | T | 0.4299 |
| YOL164W | -89 | 4 | 93 | n/a |  | AAATTAATGACA | T | 0.4301 |
| YFL055W | -86 | 1 | 87 | -306(1) |  | TAATTGATGGAT | T | 0.4301 |
| YJL199C | -56 | -33 | 24 | n/a |  | ATATAAATGCAA | T | 0.4325 |
| YHR072W | -62 | -42 | 21 | -85(1) -49(1) |  | CCAGTAATGTAC | G | 0.4328 |
| YDL115C | -26 | 7 | 33 | -425(1) |  | ATGACGATGGGT | A | 0.4354 |
| YEL072W | -23 | 1 | 24 | n/a |  | TCTATCATGCCC | A | 0.439 |
| YEL073C | -55 | -41 | 15 | n/a |  | AGCGAGATGGTG | G | 0.4429 |
| YCR089W | -94 | 56 | 150 | n/a |  | AGTACAATGGAA | A | 0.4537 |
| YHR139C | -56 | -48 | 9 | n/a |  | TCAATCATGCTC | A | 0.4578 |
| YDR282C | -23 | 22 | 45 | n/a |  | TAATAAATGATC | T | 0.4593 |
| YBR020W | -71 | -63 | 9 | n/a |  | ATTCAAATGTCA | C | 0.4609 |
| YPL034W | -37 | -8 | 30 | -336(1) |  | GAGACAATGGGT | A | 0.4728 |
| YHR120W | -41 | 4 | 45 | n/a |  | ATATAGATGGTA | T | 0.4751 |
| YER039C-A | -46 | -35 | 12 | n/a |  | ACAAGAATGGAA | A | 0.4756 |
| YPL071C | -16 | 26 | 42 | -30(1) |  | GTTTAAATGACT | T | 0.4763 |
| YLR021W | -34 | 14 | 48 | n/a |  | AAAAGAATGGGC | A | 0.4829 |
| YLR156W | -29 | -18 | 12 | n/a |  | GAAGTAATGGAC | G | 0.4856 |
| YGR154C | -52 | -23 | 30 | n/a |  | GGAGAGATGGCC | G | 0.49 |
| YNL158W | -67 | -35 | 33 | -41(1) |  | ACAGCAATGTTG | G | 0.5037 |
| YGL254W | -58 | 29 | 87 | n/a |  | ATGAAAATGACA | A | 0.5045 |
| YNR029C | -70 | 32 | 102 | n/a |  | TATTAAATGTTT | T | 0.5113 |
| YHL035C | -20 | 70 | 90 | n/a |  | ACCTCAATGGTT | T | 0.5176 |
| YCR014C | -25 | 17 | 42 | n/a |  | ATAAACATGCGA | A | 0.5211 |
| YDR538W | -68 | -54 | 15 | n/a |  | AACATAATGCTG | A | 0.5292 |
| YER188W | -55 | 98 | 153 | n/a |  | TATATAATGACA | A | 0.5439 |
| YDR530C | -76 | -68 | 9 | n/a |  | TCGAAAATGTGT | A | 0.5487 |
| YOR305W | -10 | 38 | 48 | -68(1) |  | TAAAACATGATA | A | 0.5517 |
| YDR291W | -44 | 31 | 75 | n/a |  | ATTGAGATGGTT | G | 0.5533 |
| YGL136C | -32 | 4 | 36 | -115(1) |  | GAAGTAATGACT | G | 0.5536 |
| YKL149C | -26 | 4 | 30 | -169(1) |  | ATTAAGATGGGC | A | 0.5566 |
| YOR064C | -55 | 56 | 111 | n/a |  | ACTGAAATGTAT | G | 0.5573 |
| YEL030W | -92 | -78 | 15 | n/a |  | AAAATCATGGCG | A | 0.5785 |
| YHR150W | -59 | -15 | 45 | n/a |  | ACCATAATGAAT | A | 0.5797 |
| YIR042C | -22 | 41 | 63 | n/a |  | GAAATAATGCCC | A | 0.5854 |
| YHR015W | -53 | -33 | 21 | n/a |  | AACATAATGAAT | A | 0.5866 |
| YFR046C | -7 | 29 | 36 | n/a |  | TAAGAAATGAAT | G | 0.5874 |
| YGL243W | -26 | -12 | 15 | n/a |  | AAAAAAATGAAC | A | 0.6111 |
| YFL026W | -34 | -23 | 12 | n/a |  | TTAAAAATGCAC | A | 0.6269 |
| YOR350C | -25 | 17 | 42 | n/a |  | GAAAAAATGATA | A | 0.6279 |
| YCR036W | -46 | 35 | 81 | -11(1) |  | AATAAGATGTTT | A | 0.6312 |
| YMR085W | -47 | 37 | 84 | n/a |  | TCAATCATGGCT | A | 0.6863 |
| YKL161C | -82 | -71 | 12 | n/a |  | GACAAAATGGAA | A | 0.6919 |
| YPL167C | -10 | 44 | 54 | n/a |  | AATAAAATGTTT | A | 0.7247 |
| YER185W | -74 | -54 | 21 | n/a |  | CTCAAAATGTCC | A | 0.7376 |
| YNR068C | -11 | 4 | 15 | n/a |  | AACAAAATGTAT | A | 0.7813 |
| YGL263W | -28 | 20 | 48 | n/a |  | AAAATAATGTCT | A | 0.7904 |
| YOR303W^b^ | -134 | -57 | 78 | n/a |  | CATTATATGTTT | T | 0.297 |

^a^http://data.cgt.duke.edu/5sage.php

^b^Gaba *et al.* 2005
