# Supplementary material for: Impact of Nonsense-Mediated mRNA Decay on the Global Expression Profile of Budding Yeast
Source: PLoS Genet. 2006 Nov 24;2(11):e203. doi: 10.1371/journal.pgen.0020203 (PMC1657058; doi:10.1371/journal.pgen.0020203)
Supplement: Table S11 — (49 KB DOC) [file pgen.0020203.st011.doc]

| Table S11. Nucleotide weight matrices for calculating A_UG_CAI(r) | | | | | | | | | | |
| --- | --- | --- | --- | --- | --- | --- | --- | --- | --- | --- |
| Nucleotide distribution at each position of the AUG Context^a^ | | | | | | | | | | |
|  | -6 | -5 | -4 | -3 | -2 | -1 | AUG | 4 | 5 | 6 |
| A | 20 | 20 | 29 | 53 | 43 | 44 |  | 6 | 7 | 11 |
| C | 7 | 18 | 27 | 0 | 11 | 7 |  | 6 | 35 | 10 |
| G | 13 | 3 | 0 | 9 | 0 | 12 |  | 31 | 8 | 7 |
| U | 23 | 22 | 7 | 1 | 9 | 0 |  | 20 | 13 | 35 |
| Total | 63 | 63 | 63 | 63 | 63 | 63 |  | 63 | 63 | 63 |
| Position-Specific Weight Matrices (PWMs)^b^ | | | | | | | | | | |
| ^p^A | 0.313 | 0.313 | 0.448 | 0.806 | 0.657 | 0.672 | AUG | 0.104 | 0.119 | 0.179 |
| ^p^C | 0.119 | 0.284 | 0.418 | 0.015 | 0.179 | 0.119 |  | 0.104 | 0.537 | 0.164 |
| ^p^G | 0.209 | 0.060 | 0.015 | 0.149 | 0.015 | 0.194 |  | 0.478 | 0.134 | 0.119 |
| ^p^U | 0.358 | 0.343 | 0.119 | 0.030 | 0.149 | 0.015 |  | 0.313 | 0.209 | 0.537 |
| Sum | 1 | 1 | 1 | 1 | 1 | 1 |  | 1 | 1 | 1 |
| Bit score at each position^c^ | | | | | | | | | | |
| Bit | 0.122 | 0.219 | 0.609 | 1.294 | 0.783 | 0.830 | AUG | 0.325 | 0.329 | 0.316 |
| Corrected Weight Matrices^d^ | | | | | | | | | | |
| ^w^A | 0.038 | 0.069 | 0.273 | 1.043 | 0.514 | 0.558 | AUG | 0.034 | 0.039 | 0.057 |
| ^w^C | 0.015 | 0.062 | 0.254 | 0.019 | 0.140 | 0.099 |  | 0.034 | 0.177 | 0.052 |
| ^w^G | 0.025 | 0.013 | 0.009 | 0.193 | 0.012 | 0.161 |  | 0.155 | 0.044 | 0.038 |
| ^w^U | 0.044 | 0.075 | 0.073 | 0.039 | 0.117 | 0.012 |  | 0.102 | 0.069 | 0.170 |

^a^63 highly expressed genes were included as the reference genes (Carbo*ne et a*l. 2003)

^b^reflects the nucleotide frequencies at each position (Miyasaka 1999)

^c^reflects the relative conservation of each position (Croo*ks et a*l. 2004)

^d^used for A_UG_CAI(r) calculation
