# Supplementary material for: Impact of Nonsense-Mediated mRNA Decay on the Global Expression Profile of Budding Yeast
Source: PLoS Genet. 2006 Nov 24;2(11):e203. doi: 10.1371/journal.pgen.0020203 (PMC1657058; doi:10.1371/journal.pgen.0020203)
Supplement: Table S12 — (264 KB DOC) [file pgen.0020203.st012.doc]

| Table S12. Leaky scanning analysis First AUG Second AUG  Gene Context –3 AUGCAI(r) start stop Context –3 AUGCAI(r) | | | | | | | | | |
| --- | --- | --- | --- | --- | --- | --- | --- | --- | --- |
| YDL197C | ASF2 | GTGCTTATGCCA | C | 0.1709 | 26 | 46 | TCTTGGATGCCA | T | 0.2523 |
| YLR156W | *---* | AGTCGAATGAAG | C | 0.1787 | 14 | 37 | TCCAATATGCGT | A | 0.4595 |
| YMR294W | *JNM1* | TGGTTGATGAAC | T | 0.1811 | 35 | 55 | CCATCAATGTGG | T | 0.3618 |
| YCR099C | *---* | TCCTGTATGCGA | T | 0.1861 | 77 | 145 | TGGCAGATGGGA | C | 0.2389 |
| YHR120W | *MSH1* | CCACGGATGAAG | C | 0.1934 | 197 | 268 | AGCAAGATGACA | A | 0.5242 |
| YOR026W | *BUB3* | CGCGGCATGCAG | G | 0.1977 | 95 | 106 | CTTGGGATGGCT | G | 0.365 |
| YKL149C | *DBR1* | TTGCTTATGACT | C | 0.2055 | 38 | 49 | GCTGTCATGGTC | G | 0.3644 |
| YBR055C | *PRP6* | CATTTTATGGAG | T | 0.2091 | 107 | 154 | TTAGTAATGATG | G | 0.4537 |
| YML023C | *---* | CTGAGTATGGAT | A | 0.221 | 5 | 16 | GTATGGATGGTG | T | 0.2855 |
| YHR156C | *LIN1* | AAGGATATGAAA | G | 0.2287 | 80 | 124 | TTAGCGATGAGT | G | 0.4529 |
| YER187W | *---* | GTGAGCATGCTC | A | 0.2334 | 105 | 125 | CACCTCATGGAT | C | 0.3311 |
| YLL003W | *SFI1* | TAGGTTATGGGC | G | 0.2341 | 92 | 118 | CCACTGATGTAC | C | 0.2914 |
| YIL100W | *---* | ATATGTATGTAT | T | 0.2372 | 11 | 124 | ATATATATGTTG | T | 0.3249 |
| YLR421C | *RPN13* | GTGTTGATGAGT | T | 0.2389 | 69 | 95 | ACGTCTATGTAC | T | 0.1831 |
| YOR005C | *DNL4* | TTAGGTATGATA | G | 0.2405 | 63 | 74 | TTTCAAATGGCT | C | 0.5542 |
| YBR184W | *---* | CGGGACATGTAC | G | 0.2422 | 14 | 22 | AAAATAATGTAT | A | 0.6681 |
| YLR363C | *NMD4* | AGGTTAATGACA | T | 0.2445 | 26 | 52 | TTATAGATGCTT | T | 0.4606 |
| YOL164W | *---* | CCGGATATGATT | G | 0.2452 | 54 | 86 | GAAGGAATGTCA | G | 0.4301 |
| YHL035C | *---* | CGGAGAATGGGA | A | 0.2502 | 32 | 70 | GAAATAATGGTT | A | 0.7118 |
| YMR085W | *---* | CGACCCATGGAA | C | 0.2505 | 44 | 49 | CGCGGTATGTGA | G | 0.1873 |
| YFR054C | *---* | AAGACTATGCTC | A | 0.2517 | 36 | 47 | CCCTCAATGCAT | T | 0.3702 |
| YOR377W | *ATF1* | GCTCTCATGAAT | C | 0.2547 | 5 | 52 | TCATGAATGAAA | T | 0.2835 |
| YDR438W | *---* | AGGAGAATGAAT | A | 0.2611 | 95 | 148 | TGACTAATGAAT | C | 0.32 |
| YPR196W | *MAL33* | AGGAATATGAGT | A | 0.2622 | 21 | 26 | ACAGTCATGTGA | G | 0.4054 |
| YOR350C | *MNE1* | AAGAATATGAAG | A | 0.2637 | 156 | 188 | TGATAAATGGTT | T | 0.5152 |
| YPL071C | *---* | CATTCCATGAGT | T | 0.2728 | 26 | 76 | GAAGTAATGGCA | G | 0.5804 |
| YLR097C | *HRT3* | GAGCAAATGATA | C | 0.2754 | 14 | 70 | TAGATTATGAAA | A | 0.2378 |
| YGR168C | *---* | AACTATATGAAA | T | 0.2775 | 20 | 58 | GTCCAAATGGTA | C | 0.4768 |
| YLL063C | *AYT1* | AGATGGATGTTT | T | 0.2776 | 117 | 155 | TCAAATATGCTC | A | 0.4269 |
| YGL243W | *TAD1* | ATGTGAATGGTT | T | 0.2776 | 27 | 59 | AAGGCCATGCAT | G | 0.2825 |
| YHR139C | *SPS100* | GCTATTATGAAA | A | 0.2785 | 360 | 383 | TTTCTTATGGGG | C | 0.2226 |
| YHR129C | *ARP1* | TAGCCAATGGAC | C | 0.2791 | 23 | 46 | ACAGCTATGCTT | G | 0.3437 |
| YHR150W | *PEX28* | CAGCAAATGAGT | C | 0.2794 | 335 | 451 | CAGTAAATGATT | T | 0.3182 |
| YLR318W | *EST2* | ATACTCATGAAA | C | 0.2795 | 81 | 107 | TTTAAAATGTGG | A | 0.5988 |
| YDL218W | *---* | AACGTTATGAAA | G | 0.2812 | 86 | 169 | TGGCGTATGCCA | C | 0.1163 |
| YBR217W | *ATG12* | GACAGTATGAGT | A | 0.2876 | 26 | 109 | GCGAAAATGAAA | A | 0.3986 |
| YCL056C | *---* | AGTTGAATGGTT | T | 0.2884 | 45 | 83 | AGATATATGGGA | T | 0.2787 |
| YNL204C | *SPS18* | TGGACCATGCGT | A | 0.2916 | 90 | 149 | TTTTGAATGCAA | T | 0.2501 |
| YHR134W | *WSS1* | TGATTAATGAAG | T | 0.2934 | 41 | 136 | AATACCATGATA | A | 0.4057 |
| YDR406W | *PDR15* | AAACCTATGTCA | C | 0.2987 | 137 | 268 | GGGTGCATGAGT | T | 0.1447 |
| YGL042C | *---* | GTATATATGTAC | T | 0.3014 | 14 | 103 | TCGTATATGAAT | T | 0.2171 |
| YKR012C | *---* | TTGGTAATGAAC | G | 0.3018 | 75 | 110 | GTGGACATGTTG | G | 0.3205 |
| YBR246W | *---* | ATACATATGGAC | C | 0.3053 | 23 | 31 | AATCAGATGTAC | C | 0.3328 |
| YIL058W | *---* | AAATTCATGATG | T | 0.3055 | 53 | 112 | TGTCCCATGTGG | C | 0.2255 |
| YLR288C | *MEC3* | AGTTAAATGAAA | T | 0.3074 | 23 | 58 | TAGTAAATGGTT | T | 0.4245 |
| YDR209C | *---* | CAACAGATGAAC | C | 0.3076 | 8 | 94 | TGAACGATGGTC | A | 0.4905 |
| YPR085C | *---* | GAACACATGAGA | C | 0.3158 | 17 | 25 | TTAGTAATGAGA | G | 0.4515 |
| YKR070W | *---* | GCGAGAATGATT | A | 0.3158 | 62 | 115 | ATATTGATGGTG | T | 0.3853 |
| YER186C | *---* | AGTATCATGCTG | A | 0.3158 | 17 | 37 | ATCCTGATGGTT | C | 0.4167 |
| YPL189W | *GUP2* | AGTCAGATGTCG | C | 0.3159 | 131 | 250 | AAGAGAATGGTT | A | 0.3963 |
| YJL199C | *---* | TATTGAATGTAT | T | 0.3161 | 18 | 71 | GTCAGCATGTGG | A | 0.3511 |
| YGL262W | *---* | ACTCACATGCGT | C | 0.3188 | 11 | 16 | GTAATAATGTGA | A | 0.5778 |
| YIL166C | *---* | TCATCTATGTCC | T | 0.3217 | 143 | 223 | AAGTCTATGAAT | T | 0.1871 |
| YIL165C | *NIT1* | ACTGTGATGAAA | G | 0.3228 | 62 | 211 | TGCCAGATGCGA | C | 0.2925 |
| YFL055W | *AGP3* | TCGCTAATGGCA | C | 0.3231 | 83 | 148 | CTTCGAATGTTG | C | 0.2357 |
| YBR250W | *---* | TGACTAATGTTG | C | 0.3261 | 131 | 166 | TTGGTAATGAAG | G | 0.2915 |
| YLR266C | *PDR8* | GTTGGGATGGAT | G | 0.3265 | 5 | 25 | GGATGGATGGAT | T | 0.2605 |
| YJR155W | *AAD10* | GAATGGATGGCA | T | 0.3286 | 68 | 76 | AGGGGTATGATG | G | 0.1274 |
| YAL037W | *---* | ACTGCTATGGAT | G | 0.3298 | 35 | 43 | CCATAGATGACC | T | 0.3895 |
| YER039C-A | *---* | GTAATTATGAGC | A | 0.3304 | 17 | 88 | ATAAGCATGAAT | A | 0.3824 |
| YNR074C | *AIF1* | AAATATATGACA | T | 0.3309 | 125 | 157 | CTAATTATGTGT | A | 0.4023 |
| YNR068C | *---* | TTTTCAATGAGA | T | 0.333 | 99 | 116 | ACTAGTATGTAT | A | 0.289 |
| YDR210W | *---* | AAACTAATGAGC | C | 0.3367 | 131 | 181 | CAGTCTATGTCC | T | 0.1977 |
| YCR014C | *POL4* | TCGCACATGTCT | C | 0.3387 | 227 | 280 | GCTTAAATGATG | T | 0.3556 |
| YGR024C | *THG1* | AGCGGGATGGCA | G | 0.3389 | 47 | 79 | CACACGATGTTA | A | 0.5012 |
| YDL187C | *---* | GTGAAGATGCAG | A | 0.339 | 177 | 233 | AAAGTTATGTCG | G | 0.3621 |
| YDR538W | *PAD1* | GTAGATATGCTC | G | 0.3394 | 270 | 296 | TTCAAAATGGGG | A | 0.7205 |
| YHR180W | *---* | TGCCCAATGGAA | C | 0.3394 | 89 | 112 | TCCCTGATGTTG | C | 0.3352 |
| YBR298C | *MAL31* | TTAACTATGAAG | A | 0.342 | 59 | 79 | ACTTAGATGAGA | T | 0.3228 |
| YGR289C | *MAL11* | TATAATATGAAA | A | 0.3537 | 50 | 91 | CAAAAAATGAGG | A | 0.5394 |
| YHR031C | *RRM3* | ATCCCGATGTTC | C | 0.3558 | 14 | 178 | GGTCGCATGCCT | C | 0.1967 |
| YKL209C | *STE6* | TACGTCATGAAC | G | 0.3576 | 65 | 82 | TACGGAATGACT | G | 0.4541 |
| YOR019W | *---* | ATTATTATGATT | A | 0.3585 | 23 | 187 | CACAAAATGACT | A | 0.7378 |
| YOL014W | *---* | CACCAAATGCGA | C | 0.3587 | 93 | 101 | CTGGGCATGCGG | G | 0.1679 |
| YIL167W | *SDL1* | GAATGAATGGCG | T | 0.3606 | 20 | 103 | GCCAACATGGTT | A | 0.6788 |
| YDR005C | *MAF1* | ACGACAATGAAA | A | 0.3614 | 14 | 22 | TTATTGATGAGC | T | 0.3259 |
| YGL249W | *ZIP2* | AATACTATGATT | A | 0.3623 | 36 | 41 | ATCTAAATGTAA | T | 0.4847 |
| YHL040C | *ARN1* | GTACTAATGGAG | C | 0.3658 | 47 | 76 | AGAAACATGTCT | A | 0.6387 |
| YER039C | *HVG1* | GTATTGATGATT | T | 0.367 | 89 | 136 | TTGCTTATGGCG | C | 0.2059 |
| YDR336W | *---* | TTGGCAATGGAA | G | 0.3681 | 197 | 241 | TAAACGATGTTT | A | 0.6429 |
| YPR123C | *---* | ATACTCATGTCC | C | 0.3698 | 41 | 70 | CAGACGATGACA | A | 0.3398 |
| YGR133W | *PEX4* | CATAATATGCCA | A | 0.3713 | 51 | 107 | TGATACATGTAT | T | 0.3809 |
| YPR194C | *OPT2* | GAAATTATGAGT | A | 0.3734 | 35 | 160 | TAATTGATGAGA | T | 0.3262 |
| YCR089W | *FIG2* | ACTGCAATGAAC | G | 0.3742 | 191 | 208 | ATTCGTATGTGC | C | 0.1681 |
| YIR043C | *---* | AATGCCATGGGC | G | 0.3749 | 147 | 161 | AAAACAATGGAA | A | 0.6324 |
| YGL263W | *COS12* | ATAGGCATGGAT | G | 0.3753 | 5 | 31 | GCATGGATGGAG | T | 0.2624 |
| YPL047W | *SGF11* | GTTGCGATGACC | G | 0.3759 | 38 | 49 | TTTCAAATGGGA | C | 0.4205 |
| YDR534C | *FIT1* | CCGAAAATGAAA | A | 0.3766 | 89 | 160 | CCAAAAATGGGC | A | 0.6533 |
| YDR114C | *---* | ATATAGATGAAA | T | 0.3767 | 200 | 226 | CTTCAAATGACC | C | 0.3642 |
| YIL132C | *CSM2* | CAAAGGATGGAA | A | 0.3781 | 8 | 25 | TGGAATATGAAG | A | 0.2226 |
| YDR285W | *ZIP1* | TAGAGAATGTCA | A | 0.3781 | 77 | 82 | TCAGAGATGTAG | G | 0.4836 |
| YOL162W | *---* | TACTTGATGGGC | T | 0.3791 | 147 | 167 | TTTAACATGGTC | A | 0.5634 |
| YOR318C | *---* | ATCAGCATGTGC | A | 0.3808 | 119 | 130 | CAGTCTATGCGA | T | 0.1514 |
| YLR227C | *ADY4* | CGTATAATGAAT | A | 0.3827 | 95 | 106 | CATATAATGGTG | A | 0.4918 |
| YDL214C | *PRR2* | GGAGTGATGTCG | G | 0.3832 | 173 | 274 | AATCATATGATT | C | 0.2683 |
| YDL186W | *---* | AAAAATATGCAG | A | 0.3852 | 26 | 34 | TAGAAGATGGTT | A | 0.5327 |
| YKR078W | *---* | TTAGATATGGAG | G | 0.3878 | 23 | 46 | CGTCACATGCGA | C | 0.2141 |
| YEL023C | *---* | CATTAGATGGAT | T | 0.3885 | 23 | 67 | ATATTCATGGAA | T | 0.3584 |
| YDR047W | *HEM12* | AACGCTATGGGT | G | 0.3885 | 75 | 140 | ACCGCCATGCTG | G | 0.365 |
| YIL003W | *CFD1* | TTAAGCATGGAG | A | 0.3895 | 327 | 341 | CGTGATATGGAG | G | 0.2446 |
| YDR268W | *MSW1* | AGGAAGATGTCG | A | 0.3907 | 101 | 121 | ATAGTAATGCTA | G | 0.4669 |
| YOL158C | *ENB1* | CTGAAAATGCTG | A | 0.3918 | 26 | 37 | CTAGGAATGACA | G | 0.3631 |
| YLL018C-A | *COX19* | AATCCAATGTCA | C | 0.3952 | 87 | 158 | CGGCGAATGCAC | C | 0.1323 |
| YPL034W | *---* | ATAGGAATGACC | G | 0.3985 | 39 | 95 | TTTAGAATGGCA | A | 0.5045 |
| YNR058W | *BIO3* | CAACTAATGTCC | C | 0.4005 | 29 | 46 | CACCAGATGTCG | C | 0.3939 |
| YIR002C | *MPH1* | TCTCTGATGGCT | C | 0.4009 | 14 | 85 | GTGCAGATGATT | C | 0.2733 |
| YCR102C | *---* | GAAACCATGAAG | A | 0.4023 | 168 | 173 | TCTGGGATGTGA | G | 0.2917 |
| YLR021W | *---* | GCAAATATGATA | A | 0.405 | 11 | 55 | TATCATATGAAT | C | 0.2559 |
| YGL136C | *MRM2* | TACAGCATGATT | A | 0.407 | 59 | 97 | GAATACATGTAC | T | 0.3775 |
| YHR072W | *ERG7* | AAACAGATGACA | C | 0.4076 | 71 | 79 | GAACTGATGAGC | C | 0.2799 |
| YDL115C | *IWR1* | AATCAAATGGTG | C | 0.4119 | 8 | 19 | TGGTGAATGACT | T | 0.2179 |
| YDR282C | *---* | TGAAGAATGCTT | A | 0.4131 | 104 | 127 | TGCAAGATGGCC | A | 0.6243 |
| YCL004W | *PGS1* | ATATTAATGACG | T | 0.4149 | 165 | 248 | ACAAACATGCTT | A | 0.6056 |
| YPR135W | *CTF4* | ACTATTATGGTT | A | 0.4154 | 98 | 106 | ATAAAAATGGCT | A | 0.9851 |
| YOR190W | *SPR1* | AAGTAAATGGTT | T | 0.4176 | 152 | 190 | CCATCAATGAAA | T | 0.3305 |
| YPR170C | *---* | TGAGCAATGACG | G | 0.423 | 26 | 166 | CACCAAATGCCG | C | 0.4003 |
| YBR253W | *SRB6* | AAGAAAATGAGC | A | 0.4239 | 20 | 58 | CACTATATGAGA | T | 0.2536 |
| YPL164C | *MLH3* | AAGAAAATGAGC | A | 0.4239 | 32 | 76 | ATTCTGATGTTT | C | 0.3463 |
| YOL068C | *HST1* | ATCTAAATGAAC | T | 0.4247 | 68 | 82 | ACTTTCATGTTG | T | 0.2946 |
| YCL016C | *DCC1* | TGTACCATGTCC | A | 0.4254 | 32 | 52 | CCGAGTATGATC | A | 0.1708 |
| YIL168W | *---* | GTTACCATGGAA | A | 0.4342 | 17 | 34 | CTTACTATGAAA | A | 0.2742 |
| YBL005W | *PDR3* | ACCGCAATGAAA | G | 0.4342 | 42 | 83 | GACAGCATGTGT | A | 0.4108 |
| YBR008C | *FLR1* | TCCACTATGGTA | A | 0.4382 | 101 | 160 | GTGAAGATGAAA | A | 0.3546 |
| YEL030W | *ECM10* | AGTAACATGTTA | A | 0.44 | 12 | 26 | ACCATCATGGAA | A | 0.5015 |
| YJR003C | *---* | ATCACCATGAAA | A | 0.4414 | 14 | 40 | AGTTCGATGAGA | T | 0.2349 |
| YFR046C | *CNN1* | TGAATAATGAGC | A | 0.4437 | 104 | 115 | TCAAGGATGAGG | A | 0.3447 |
| YFL003C | *MSH4* | TATGCAATGAGT | G | 0.445 | 183 | 197 | GACAACATGGGA | A | 0.5787 |
| YCR020C | *PET18* | AGAACAATGAGC | A | 0.4453 | 84 | 110 | GGAACTATGTGC | A | 0.3134 |
| YKL026C | *GPX1* | GTAACGATGCAA | A | 0.4484 | 32 | 85 | CAATAGATGAAA | T | 0.3366 |
| YEL072W | *RMD6* | AAACTAATGTCA | C | 0.4486 | 104 | 136 | GAGGATATGATG | G | 0.2223 |
| YPR200C | *ARR2* | AGCGTAATGGTA | G | 0.4513 | 122 | 133 | CTTGGCATGTTC | G | 0.2606 |
| YIR042C | *---* | ACAATTATGGCG | A | 0.4522 | 131 | 226 | AAAGACATGGTT | G | 0.6014 |
| YNL331C | *AAD14* | TTCAGCATGACT | A | 0.4562 | 126 | 155 | CGACGCATGGTC | C | 0.2011 |
| YDR013W | *PSF1* | AGAATCATGTAT | A | 0.458 | 5 | 16 | TCATGTATGGAG | T | 0.2094 |
| YPL167C | *REV3* | TTGGAAATGTCG | G | 0.4592 | 227 | 271 | ACGTCCATGGCA | T | 0.2898 |
| YNL158W | *---* | AAGAAGATGGTC | A | 0.4594 | 17 | 154 | CTCAGAATGTTC | A | 0.4376 |
| YPL066W | *---* | GTGAAAATGACG | A | 0.4604 | 90 | 167 | AATCGAATGCCA | C | 0.2663 |
| YDR147W | *EKI1* | GGCATAATGTAC | A | 0.4608 | 149 | 172 | ATACTAATGAGC | C | 0.3398 |
| YHR199C | *---* | GGAAACATGCGT | A | 0.4623 | 69 | 188 | CCCACAATGGTA | A | 0.5957 |
| YOR303W | *CPA1* | TTTCAAATGTCC | C | 0.4637 | 41 | 139 | TCCAAAATGGTC | A | 0.7679 |
| YOL165C | *AAD* | ATTCCAATGGCT | C | 0.4718 | 33 | 68 | CGCCCCATGGGA | C | 0.2519 |
| YGR263C | *---* | AGAAGAATGGCA | A | 0.473 | 56 | 118 | AAGAGAATGAAA | A | 0.2783 |
| YLR165C | *PUS5* | ATTTTAATGTCT | T | 0.4782 | 102 | 122 | CAGAACATGGGG | A | 0.3606 |
| YDR332W | *---* | TAAAATATGACC | A | 0.4796 | 116 | 124 | AACAAGATGCTA | A | 0.5683 |
| YLR263W | *RED1* | ATAAGAATGGAA | A | 0.4858 | 44 | 52 | TGAAAAATGACT | A | 0.6963 |
| YIL059C | *---* | TCCATCATGAAT | A | 0.4862 | 92 | 133 | TAGGCTATGAAG | G | 0.1923 |
| YNR029C | *---* | GGAAGAATGTCT | A | 0.4866 | 29 | 64 | AGTTCAATGAAG | T | 0.2544 |
| YLR233C | *EST1* | TAGATAATGGAT | A | 0.4869 | 8 | 34 | TGGATAATGAAG | A | 0.2894 |
| YIR029W | *DAL2* | AGAAAAATGAAG | A | 0.4903 | 23 | 46 | TGGCAGATGAGG | C | 0.1929 |
| YOL134C | *---* | TCCGTCATGGCC | G | 0.4948 | 26 | 55 | CAATGCATGGTT | T | 0.2991 |
| YKL161C | *---* | AATTAAATGGCG | T | 0.4954 | 293 | 304 | ACCCAAATGGGG | C | 0.4447 |
| YGR154C | *---* | TAAGCCATGTCA | G | 0.4966 | 299 | 304 | TAGGTGATGTGA | G | 0.3013 |
| YOR162C | *YRR1* | GTCACAATGAAA | A | 0.5106 | 17 | 154 | GAAGCGATGCTT | G | 0.443 |
| YOL104C | *NDJ1* | ACTATAATGAGT | A | 0.5115 | 77 | 127 | AATTCCATGACT | T | 0.353 |
| YKR069W | *MET1* | AAAAGAATGGTA | A | 0.5128 | 143 | 163 | TTTTGAATGCTG | T | 0.2547 |
| YLR174W | *IDP2* | ATCGTAATGACA | G | 0.5143 | 47 | 64 | ACGGCGATGAGC | G | 0.2618 |
| YMR064W | *AEP1* | TAGAAAATGATT | A | 0.5167 | 30 | 53 | CAGCAAATGGAG | C | 0.2763 |
| YOL015W | *---* | TCAATCATGTTC | A | 0.5173 | 191 | 220 | ATCCAGATGAAA | C | 0.345 |
| YIL029C | *---* | AAAACAATGCGG | A | 0.5177 | 192 | 257 | TCCAAGATGTAC | A | 0.5992 |
| YML099C | *ARG81* | GATATAATGGGA | A | 0.518 | 20 | 343 | GCAAGAATGGCC | A | 0.5317 |
| YOR064C | *YNG1* | ACAGCAATGGAA | G | 0.5181 | 75 | 80 | TTTACCATGTGA | A | 0.4473 |
| YEL057C | *---* | AATTAAATGGCA | T | 0.5182 | 8 | 91 | TGGCAAATGATG | C | 0.2329 |
| YOL130W | *ALR1* | TTTACCATGTCA | A | 0.5221 | 239 | 331 | ACAGCCATGTGG | G | 0.3954 |
| YNL260C | *---* | TCAATCATGGTG | A | 0.5233 | 78 | 119 | GAGTAAATGGAA | T | 0.3314 |
| YOL140W | *ARG8* | CCAATCATGTTT | A | 0.5235 | 102 | 113 | AGATCTATGTAT | T | 0.2565 |
| YJL065C | *DLS1* | AACACAATGAAC | A | 0.5247 | 92 | 133 | CCAAGAATGATC | A | 0.3822 |
| YDR174W | *HMO1* | CACACCATGACT | A | 0.5269 | 116 | 148 | TCTACAATGCCA | A | 0.5483 |
| YER188W | *---* | ACAATAATGATG | A | 0.5271 | 170 | 181 | CTAGTAATGGAT | G | 0.5282 |
| YOR305W | *---* | TAAAACATGATA | A | 0.5517 | 27 | 38 | ACGAAGATGGCT | A | 0.575 |
| YDR291W | *---* | GGAAAAATGGAG | A | 0.5539 | 62 | 121 | AGACCGATGCCT | C | 0.3309 |
| YOL108C | *INO4* | GCAATAATGACG | A | 0.5587 | 80 | 85 | TGGCTAATGTGA | C | 0.2221 |
| YFL050C | *ALR2* | TTCGTAATGTCG | G | 0.5646 | 128 | 163 | ATTCTGATGCAC | C | 0.2522 |
| YIR041W | *PAU3* | AATATAATGGTC | A | 0.5647 | 227 | 250 | TCAACTATGGTG | A | 0.4223 |
| YML050W | *---* | AATAAAATGCTA | A | 0.5681 | 98 | 130 | CTCAAAATGATC | A | 0.5879 |
| YGL146C | *---* | GAAAACATGGAA | A | 0.5756 | 75 | 92 | CCGAGGATGGAA | A | 0.2557 |
| YDR265W | *PEX10* | ACCAAAATGAAG | A | 0.5785 | 8 | 55 | TGAAGAATGATA | A | 0.3658 |
| YNL335W | *---* | TCAATCATGTCA | A | 0.5802 | 62 | 145 | TGGTGAATGCAC | T | 0.1615 |
| YEL073C | *---* | AACACGATGGTA | A | 0.5822 | 54 | 62 | GAGCGCATGGTC | C | 0.1754 |
| YDR399W | *HPT1* | CCCATAATGTCG | A | 0.5916 | 51 | 68 | TCAACTATGTCA | A | 0.4682 |
| YOR313C | *SPS4* | ACAAACATGCCA | A | 0.5956 | 77 | 187 | ACACGAATGAAC | C | 0.2549 |
| YBR020W | *GAL1* | ACTATAATGACT | A | 0.5971 | 119 | 169 | GCGCTTATGATG | C | 0.144 |
| YOL009C | *MDM12* | ATCCAAATGTCT | C | 0.5978 | 50 | 61 | GACTGAATGATC | T | 0.2819 |
| YGL254W | *FZF1* | AAAACAATGACG | A | 0.6042 | 39 | 50 | TTACAAATGTTC | C | 0.4836 |
| YMR114C | *---* | CCCAAGATGTGT | A | 0.6147 | 116 | 241 | ATCCACATGACG | C | 0.3696 |
| YLL057C | *JLP1* | TAAAATATGTCT | A | 0.6181 | 71 | 79 | TTACAAATGGGT | C | 0.5497 |
| YDR242W | *AMD2* | TATACAATGACT | A | 0.6265 | 75 | 119 | AGACGAATGGAA | C | 0.2562 |
| YNL254C | *---* | ACAGAGATGGTT | G | 0.6273 | 107 | 184 | GAAAACATGACA | A | 0.5753 |
| YEL028W | *---* | TAAAAAATGAAA | A | 0.6275 | 80 | 121 | TTCATAATGGTG | A | 0.6426 |
| YHR015W | *MIP6* | AAAAAGATGCCA | A | 0.6361 | 14 | 28 | ACTCTCATGGTA | C | 0.2981 |
| YOR009W | *TIR4* | AAAAATATGGCT | A | 0.6371 | 95 | 280 | TTTTAGATGATG | T | 0.3368 |
| YDR530C | *APA2* | ACAAAGATGATT | A | 0.6392 | 59 | 70 | AAAAGAATGGGC | A | 0.4829 |
| YOR100C | *CRC1* | TCAGTAATGTCT | G | 0.6582 | 141 | 179 | CGGGGTATGTGC | G | 0.1279 |
| YGR122C-A | *---* | CTCAAAATGGAA | A | 0.6598 | 18 | 23 | GTCCCTATGTAA | C | 0.2413 |
| YPL133C | *RDS2* | GAAATAATGTCA | A | 0.6682 | 48 | 62 | TAAAACATGTTT | A | 0.7038 |
| YOR381W | *FRE3* | AAAACAATGTAT | A | 0.6816 | 36 | 71 | TTTGTTATGCTG | G | 0.2558 |
| YLL055W | *---* | AAAGAAATGTCA | G | 0.6841 | 50 | 61 | CTTCTGATGAAA | C | 0.2298 |
| YER139C | *---* | TAAGAAATGGCG | G | 0.6964 | 71 | 88 | CCATGCATGAAG | T | 0.1984 |
| YMR316C-B | *---* | GAAAAAATGGAA | A | 0.6975 | 11 | 64 | AAACCAATGACT | C | 0.4573 |
| YIL164C | *NIT1* | ACCACAATGGCG | A | 0.7011 | 83 | 181 | TGTCATATGAGA | C | 0.1908 |
| YMR106C | *YKU80* | ATAATAATGTCA | A | 0.7066 | 32 | 49 | TCGTGGATGTTT | T | 0.2297 |
| YDR402C | *DIT2* | TTAAAAATGGAG | A | 0.7166 | 50 | 82 | TTTCCTATGTCG | C | 0.253 |
| YDR403W | *DIT1* | ACAAAAATGACA | A | 0.7217 | 110 | 190 | CCCATAATGGGG | A | 0.531 |
| YJR152W | *DAL5* | TTCAAGATGTCA | A | 0.7314 | 11 | 40 | CAGCGGATGCTA | C | 0.1493 |
| YCR036W | *RBK1* | TCAAAGATGGGT | A | 0.7315 | 32 | 124 | TAAACTATGATT | A | 0.4264 |
| YNL014W | *HEF3* | CACAAAATGTCA | A | 0.7383 | 131 | 223 | TCGAGCATGACG | A | 0.2609 |
| YBL075C | *SSA3* | AGAAAAATGTCT | A | 0.774 | 62 | 73 | TTTCCAATGATA | C | 0.3232 |
| YHR153C | *SPO16* | ATAGAAATGTCT | G | 0.7796 | 20 | 85 | TTTGGGATGTAC | G | 0.291 |
| YER185W | *---* | TCCAAAATGTCC | A | 0.8139 | 119 | 148 | CTTTATATGCTG | T | 0.224 |
| YBR278W | *DPB3* | AAAAAAATGTCC | A | 0.8168 | 66 | 95 | TGCCAAATGCGA | C | 0.3358 |
| YFL034C-A | *RPL22B* | ACCACAATGGCT | A | 0.8281 | 131 | 142 | AAGTAGATGGTG | T | 0.308 |
| YGL096W | *TOS8* | AAAAAAATGGGT | A | 0.8362 | 83 | 157 | GAGAAAATGAAA | A | 0.4033 |
| YKR102W | *FLO10* | TAAAAAATGCCT | A | 0.8382 | 59 | 70 | TAGCTAATGTTG | C | 0.2687 |
| YNL057W | *---* | ATAAAAATGGCA | A | 0.8725 | 71 | 88 | AAATAAATGGGT | T | 0.5804 |
| YFL026W | *STE2* | TCAAAAATGTCT | A | 0.9358 | 8 | 25 | TGTCTGATGCGG | C | 0.2065 |
