# Supplementary material for: Impact of Nonsense-Mediated mRNA Decay on the Global Expression Profile of Budding Yeast
Source: PLoS Genet. 2006 Nov 24;2(11):e203. doi: 10.1371/journal.pgen.0020203 (PMC1657058; doi:10.1371/journal.pgen.0020203)
Supplement: Text S1 — (542 KB DOC) [file pgen.0020203.sd001.doc]

### Use of RNA Decay Models in the Analysis of Array Data

Four theoretical models describing the kinetics of mRNA decay were developed and evaluated for their ability to explain changes in mRNA abundance over a one-hour time course following inhibition of transcription in the presence of 10 $m$g/ml thiolutin (see Materials and Methods). In each model, $X_{1},X_{2},L,X_{n}$ denotes time after transcription inhibition, $Y_{1},Y_{2},L,Y_{n}$ denotes mRNA abundance at *n* time points. $\beta_{1}$, the decay rate parameter, is inversely related to mRNA half life (). Other parameters are defined separately for each model as described below. is the experimental error.

**Model 1: Naïve first-order decay.** If transcripts made prior to transcription inhibition decay after inhibition according to first-order kinetics (exponential decay), the decay is described by:

NMD-sensitive transcripts have the common feature that their levels of abundance are relatively low in wild-type yeast strains [1,2, and this study]. By examining the scatter plot of *Y* *vs.* *X*, it was observed that the decay curves flatten out at later time points following inhibition of transcription. This is also a common phenomenon observed when the half-lives of low-abundance transcripts are examined by Northern hybridization [3]. These decay curves fail to conform to first-order exponential decay. Although departures from first-order decay could have biological causes in some cases (e.g. an RNA population might consist of sub-populations with different decay rates), the most likely global cause of the departure is that transcription inhibition by thiolutin is incomplete. A low level of on-going transcription would give rise to "tails" on the decay curves that would distort the results when fitting the data to an exponential decay model. The distortion would be most evident for non-abundant transcripts with rapid decay rates.

**Model 2: Non-first order decay.** A model developed to account for a departure from first-order decay due to residual transcription is described by:

The reduced rate of transcription in the presence of the transcriptional inhibitor is assumed to be constant. This model was arrived at by solving the ordinary differential equation $m^{'}(X)=c+\beta_{1}m(X)$, where $m(X)$ is the expected amount of RNA at time *X*, $\beta_{1}$ is the decay parameter related to half-life as in Model 1, and $m^{'}(X)$ represents the rate of change of mRNA abundance with respect to time. The decay rate is composed of two parts: *c*, representing residual transcription at a constant rate, and $\beta_{1}Y$, representing the first-order exponential decay of the transcript. This equation has the solution , with . At *X*=0, is the initial mRNA abundance upon the addition of thiolutin. Model 2 captures biphasic decay patterns including the patterns typical of low abundance transcripts that are distorted by incomplete inhibition of transcription. For relatively abundant transcripts where low-level residual transcription is not evident during a one-hour time course ($c_{0}=0$), the decay rate reverts to first-order exponential decay. Therefore, it appears that Model 2 is better suited than Model 1 for the analysis of the array data. However, since Model 2 has one more parameter than Model 1, additional criteria described below were used before making a choice.

**Model 3:** **Truncated first-order decay.** Model 3 provides an alternative explanation to the “flat tail” problem. It assumes that the tail is caused by hitting an experimental threshold of detection, *c*, which prevents the ability to distinguish differences in mRNA abundance at later time points following inhibition of transcription. The model is described as follows:

where *c* is a constant and $x_{t}$, which can vary for each transcript, is the amount of time that has transpired between inhibition of transcription and appearance of the “tail”.

**Model 4:** **Differential decay of two RNA sub-populations.** A model was also considered that describes the decay of two sub-populations of the same RNA each of which decays at a different rate. The model is described by:

where *a* and *b* are the initial mRNA amounts of the two groups respectively, $\beta_{1}$ and $\beta_{2}$ are the decay rates of each RNA sub-population. The difference between this model and model 2 is that both lines describing each decay rate are permitted to have a non-zero slope. To avoid confounding of the two exponential terms, additional constraints have to be added in model fitting process.

###### Performance assessment

Simulated data were used to compare the performance of the models by Akaike and Bayesian Information criteria (AIC*/*BIC) criteria [3,4] (Tables S5 and S6). In each model, . Therefore, the response , where in Model 1, ; in Model 2, ; in Model 3,

and in Model 4, .

If the true distribution of data *Y* is *f*(*Y*), and *g*(*Y*) is the density specified by the model, then their discrepancy $f(x)\text{dx}$ can be estimated by AIC = , where *r* is the dimension of the parameter vector $q$. For Model 1, *r* = 2; for Model 2, *r* = 3; for Model 3, *r* = 4, and for Model 4, *r* = 4. In all four models,

(1)

If the maximum likelihood estimates $\hat{q}$ and $\hat{s}^{2}=\text{RSS}/n$ are known (*RSS* is the residual sum of squares of the fitted model and *n* is the sample size), then AIC can be written as:

(2)

Another commonly used criterion, BIC [17], has a slightly different form:

(3)

In order to assess performance, each non-linear model was fitted using algorithms for profiling $\beta_{1}$ that convert them into linear models, as follows:

Naïve first-order decay (Model 1). (1) For a fixed $\beta_{1}$, a design matrix, $\tilde{X}^{\beta_{1}X}$, was created to generate a linear model $Y=\tilde{X}_{0}$ The least squares estimator was calculated by . $\text{RSS}(\beta_{1})$ was also calculated. (2) $-\text{ln}2/{\beta_{1}}$ was profiled across [0.1, 240] at resolution of 0.24. $\text{RSS}(\beta_{1})$ was calculated and compared at each possible value of $\beta_{1}$. (3) The value of $\hat{\beta}_{1}$ that minimizes $\text{RSS}(\beta_{1})$ was identified. is the least squares estimator for Model 1. is the *RSS* of the fitted model.

Non-first order decay (Model 2). (1) For a fixed $\beta_{1}$, a design matrix was created to generate a linear model $Y=\tilde{X}_{1}$, where The least squares estimator $\hat{\theta}(\beta_{1})$ was calculated by . The sum of squares error of the fitted model $\text{RSS}(\beta_{1})$ was also calculated. (2) $-\text{ln}2/{\beta_{1}}$ was profiled across [0.1, 240] at resolution of 0.24. $\text{RSS}(\beta_{1})$ was calculated and compared at each possible value of $\beta_{1}$. (3) The value of $\hat{\beta}_{1}$ that minimizes $\text{RSS}(\beta_{1})$ was identified. is the least squares estimator for Model 2. is the *RSS* of the fitted model.

Truncated first-order decay (Model 3). (1) Let *t*=3, and divide the data into two parts: and . (2) For the first *t* observations, Model 1 was fitted by profiling $-\text{ln}2/{\beta_{1}}$. $X=(x_{1},L,x_{t})^{T}$ and $Y=(y_{1},L,y_{t})^{T}$ were used in steps 1-3 of Model 1 described above. The minimal $\text{RSS}(\beta_{1})$ at $\hat{\beta}_{1}$ was calculated and denoted as $\text{RSS}_{1}$. (3) For the other *n*-*t* observations, the model $Y=c+e$ was fitted. and were calculated. (4) For $t=4,L,15$, steps 1-3 were repeated, and the argument $t_{m}$ that minimizes was found. The minimum of is the residual sum of squares for the fitted model. The corresponding parameters are obtained in step 2 of the iteration $t=t_{m}$, and .

Differential decay (Model 4). (1) For a fixed $\beta_{1}$ and a fixed $\beta_{2}$, a design matrix was created to generate a linear model $Y=\tilde{X}_{1}$, where The least squares estimator was calculated by . The sum of squares error of the fitted model was also calculated. (2) Both $-\text{ln}2/{\beta_{1}}$ and $-\text{ln}2/{\beta_{2}}$ were profiled across [0.1, 240] at resolution of 0.24, with the constraint that , which is set up to avoid confounding of the two exponential terms. was calculated and compared at each possible pairs of $\beta_{1}$ and $\beta_{2}$. (3) The value of that minimizes was identified. is the least squares estimator for Model 4. is the *RSS* of the fitted model.

The four models were fitted for *n* observations using the algorithms described above to obtain each *RSS*. By substituting the values for *RSS*, *n* and *r* for each model in equations (2) and (3), the AIC/BIC values were calculated. The model giving the best fit is the one with the smallest AIC/BIC. 48 observations were simulated from models 1 through 4 (3 trials × 16 time points), and the simulated data were fitted and compared using AIC/BIC. This simulation was repeated 1000 times, and the number of times each model had the smallest AIC*/*BIC was determined.

The results indicate that Model 2 (non-first order decay) tolerates data obeying Model 1 (naïve first-order decay) or Model 4 (differential decay) more than Model 1 or 4 tolerates data obeying Model 2. This indicates that when there is uncertainty about whether mRNA decay obeys first-order or non-first order kinetics, the latter should be assumed. In addition, when experimental data for a given mRNA is best fitted by Model 3, the most appropriate assumption is that sufficient sensitivity is lacking to measure decay with only 16 time points across a one-hour time window. Based on our analyses, Model 2 was used to estimate changes in decay rates when wild-type and *upf1^-^* strains were compared. None of the 607 NMD-sensitive probe sets identified by SAM were eliminated from the analysis by virtue of conforming to Model 3.

### Estimation and comparison of decay rates

Model 2 described above shows the relationship between the expected amount of RNA and time. If $e$ is the experimental error, and assuming , we can fit the data with the regression model , with the parameter vector . Three independent experiments were done for the Nmd^-^ strain, denoted as $i=1,2,3$, and three for the wild-type strain, denoted as $i=4,5,6$. For each probe set, six regression lines can be fitted that describe mRNA decay. Each regression line contains data observed from 16 time points: 0, 2, 4, 6, 8, 10, 12, 15, 20, 25, 30, 35, 40, 45, 50, 60, ($j=1,L,16$). The log-likelihood function of $q$ for one group and the total likelihood function for six groups of experiments are derived as follows.

The likelihood function of $q$ for experiment *i* is:

(4)

(5)

(6)

where $q^{(i)}$ is the parameter of interest in experiment *i*, $1\leq i\leq6$. is the residual sum of squares of the *i*th regression line for a probe set. Since there are six regression lines, the total likelihood function is:

(7)

where $L^{(i)}(q^{(i)},s)$ is the likelihood function with *i*th group of data substituted in (4).

The best estimate of parameter vector $q$ is the argument that minimizes $L$ in (4) with respect to the linear constrains , and . They are denoted as and when $i=1,2,3$ and $i=4,5,6$ respectively. Since $y^{(i)}$ values are significantly different when $i=1,2,3$ and $i=4,5,6$, the variance $\sigma^{2}$ of $y^{(i)}$ is allowed to be different between mutant and wild type strains, but to be the same within each group. They are denoted as and . Equation (7) can be further written as:

(8)

(9)

(10)

If (10) is 0, is obtained. Similarly, . By substituting these terms in (8), we obtain:

(11)

(12)

The best estimate of parameter vector $q$ is the argument that maximizes the total likelihood with respect to the linear constraints , and . Since $y^{(i)}$ values representing mRNA abundance are significantly different when $i=1,2,3$ and $i=4,5,6$, the variance $\sigma^{2}$ of $y^{(i)}$ is allowed to be different between mutant and wild type strains, but to be the same within each group. Therefore the best estimate of $q$ is

(13)

where is the residual sum of squares of the *i*th regression line for a probe set. The likelihood ratio $\lambda$ can be used to test the hypothesis , where:

(14)

To implement the estimation of $\hat{q}$ by extensive profiling of $\beta_{1}$, it was assumed that the mRNA half life is 100 minutes or less, $-\text{ln}2/{\beta_{1}}$ was profiled in the interval of (0, 100] at a resolution of 0.1. For each fixed $\beta_{1}$, a unique least squares estimator and were determined. A 2-dimensional profiling of $\beta_{1}^{m}$ (for $i=1,2,3$) and $\beta_{1}^{w}$ (for $i=4,5,6$) was performed to construct a $1000\times1000$ matrix $A=[a_{\text{rs}}]$, where , $1\leq r,s\leq1000$. The best estimate of $\beta_{1}$ is obtained at (*R,S*) where $a_{\text{RS}}$ is the minimum element of *A*, while the best estimate of $\beta_{1}$ under $H_{0}$ is obtained at (*D,D*), where $a_{\text{DD}}$ is the minimum diagonal element of *A*. Since , .

### Reference line

Since equal amounts of cRNA were added to the array hybridization chamber to generate data from each array at each time point and since the transcripts decay at different rates over time, a control was required in order to normalize the expression levels of each transcript at each time point to one or several stable transcripts (the reference line) that undergo negligible decay during the one-hour time-course. Internal spike controls proved inadequate in providing a reference line because the levels of cross hybridization were too high to be used in the analysis of low abundance transcripts.

The database of Wang et al. [5] was screened to identify reference transcripts meeting the criteria of an mRNA half-life greater than 60 minutes and no known role in the heat-shock response since thiolutin reportedly induces transcription of heat-shock genes [6]. The temperature-sensitive mutation *rpb1-1* (large subunit of RNA polymerase II) was used by Wang et al. to inhibit transcription following a temperature-shift. We justified a screen of their database to generate a reference line for use in our database because their global transcript stability profile most closely resembles the profile obtained when thiolutin is used to inhibit transcription as opposed to other chemical inhibitors of transcription [7].

110 candidate probe sets were found that satisfied the criteria described above (Table S4). To obtain the average trend line for these stable reference transcripts, their signal $y_{\text{ij}}$ at time point *j* was divided by , and was calculated. This was done to avoid potential distortion of the average trend line by highly abundant transcripts that have a large $y_{\text{ij}}$. A stable reference line based solely on the average of these 110 probe sets suffers the disadvantage that each probe set in this pool still decays slowly, and their real decay rates vary from 60 minutes to 240 minutes. Therefore, a stable reference line was generated by penalizing the average trend line obtained from the 110 probe sets by a factor of $e^{j\tau}$, where $j=1,\cdots,16$. was used as the *j*th point on the reference line.

To identify the proper penalizing factor $t$, use was made of information derived from a subset of NMD-sensitive transcripts whose accumulation and decay were evaluated by single transcript Northern blotting experiments [3]. The subset consists of 14 probe sets corresponding to 14 transcripts that exhibit differential decay rates in wild-type and *upf1^-^* strains (direct NMD targets) and 11 probe sets corresponding to 10 transcripts that exhibit differential accumulation in wild-type and *upf1^-^* and strains but without a change in decay rate (indirect NMD targets). For each fixed $t$, the normalized raw data for these 25 probe sets was used as input into the program estimating $\beta_{1}$ and , and a likelihood ratio $\lambda$ was obtained. Theoretically, since direct NMD targets should have larger $-2\text{ln}\lambda$ values than indirect NMD targets, we evaluated whether specific $\tau$ values separated these two known groups by the Wilcoxon rank sum test. $\tau$was extensively profiled in the interval of [0, 0.25] at a resolution of 0.005. When $\tau=0.155$, the rank sum of direct NMD targets by sorting $-2\text{ln}\lambda$ was the largest. For this reason, we chose the penalizing factor 0.155 to form the stable reference line, and normalized the data for all 607 probe sets representing the NMD targets by replacing $y_{\text{ij}}$ by .

The following approach was used to visualize the number of targets with FCR values < 1. Since $-2\text{ln}\lambda$ converges to $\chi_{1}^{2}$ in distribution, the hypotheses were tested using the likelihood ratio as described in equation (14), where the *p*-values were derived using . The $-2\text{ln}\lambda$ testing statistic and the corresponding *p*-values were calculated for all 607 SAM-selected probe sets, and the relationship between $-2\text{ln}\lambda$and the estimated fold change in half life was plotted. Larger differences in half-life correlated with larger $-2\text{ln}\lambda$ values (smaller *p*-values).

**Supporting information**

Table S4. List of probe sets used to generate a stable reference line.

Table S5. Performance assessments of decay model using simulated data

Table S6. Performance assessments of decay model using actual data

**Abbreviations.** AIC, Akaike information criterion; BIC, Bayesian information criterion; FCR, fold change ratio; FDR, false discovery rate; MLE, maximum likelihood estimation; NMD: nonsense mediated decay; pdf, probability density function; RSS, residual sum of squares; SAM, significance analysis of microarrays

### References

1. Lelivelt MJ, Culbertson MR (1999) Yeast Upf proteins required for RNA surveillance affect the global expression of the yeast transcriptome. Mol Cell Biol 19: 6710-6719.

2. He F, Li X, Spatrick P, Casillo R, Dong S, et al. (2003) Genome-wide analysis of mRNAs regulated by the nonsense-mediated and 5' to 3' mRNA decay pathways in yeast. Mol Cell 12: 1439-1452.

3. Akaike H (1973) Information theory and an extension of the maximum likelihood principle. In: Petrov BNC, Frigyes, editor. 2nd International Symposium on Information Theory, Tsahkadsor, Armenia, USSR, September 2-8, 1971 : [papers]. Budapest: Akadémiai Kiadó. pp. 267-281.

4. Box GEP, Jenkins GN, Reinsel GC (1994) Time series analysis: forecasting and control. 3rd ed. Englewood Cliffs, N.J.: Prentice Hall. pp. 200-201.

5. Wang Y, Liu CL, Storey JD, Tibshirani RJ, Herschlag D, et al. (2002) Precision and functional specificity in mRNA decay. PNAS 99: 5860-5865.

6. Grigull J, Mnaimneh S, Pootoolal J, Robinson MD, Hughes TR (2004) Genome-Wide Analysis of mRNA Stability Using Transcription Inhibitors and Microarrays Reveals Posttranscriptional Control of Ribosome Biogenesis Factors. Mol Cell Biol 24: 5534-5547.
